# Advances in the pathology and treatment of osteoarthritis

**DOI:** 10.1016/j.jare.2025.01.053

**Published:** 2025-01-30

**Authors:** Xueliang Peng, Xuanning Chen, Yifan Zhang, Zhichao Tian, Meihua Wang, Zhuoyue Chen

**Affiliations:** a Provincial Key Laboratory of Biotechnology of Shaanxi, Key Laboratory of Resource Biology and Modern Biotechnology in Western China, Faculty of Life Science, Northwest University, 229 North Taibai Road, Xi’an, Shaanxi Province 710069, China; b College of Stomatology, Shanghai Jiao Tong University, Shanghai 200215, China

**Keywords:** Osteoarthritis, Articular cartilage, Mechanism, Treatment, Pain

## Abstract

**Background:**

Osteoarthritis (OA), a widespread degenerative joint disease, predominantly affects individuals from middle age onwards, exhibiting non-inflammatory characteristics. OA leads to the gradual deterioration of articular cartilage and subchondral bone, causing pain and reduced mobility. The risk of OA increases with age, making it a critical health concern for seniors. Despite significant research efforts and various therapeutic approaches, the precise causes of OA remain unclear.

**Aim of review:**

This paper provides a thorough examination of OA characteristics, pathogenic mechanisms at various levels, and personalized treatment strategies for different OA stages. The review aims to enhance understanding of disease mechanisms and establish a theoretical framework for developing more effective therapeutic interventions.

**Key scientific concepts of review:**

This review systematically examines OA through multiple perspectives, integrating current knowledge of clinical presentation, pathological mechanisms, and associated signaling pathways. It assesses diagnostic methods and reviews both pharmacological and surgical treatments for OA, as well as emerging tissue engineering approaches to manage the disease. While therapeutic strategies such as exercise, anti-inflammatory drugs, and surgical interventions are employed to manage symptoms and modify joint structure, none have been able to effectively halt OA’s advancement or achieve long-lasting symptom relief. Tissue engineering strategies, such as cell-seeded scaffolds, supportive matrices, and growth factor delivery, have emerged as promising approaches for cartilage repair and OA treatment. To combat the debilitating effects of OA, it is crucial to investigate the molecular basis of its pathogenesis and seek out innovative therapeutic targets for more potent preventive and treatment strategies.

## Highlights

• Pathological characteristics and Pathogenesis of OA, gene expression pattern are included.
• Almost all signaling pathways and histological assessment systems associated with osteoarthritis have been summarized.
• Different treatments used for patients with osteoarthritis of various severities are reviewed.
• The role of vascularization in OA and the importance of reducing vascularization in the treatment of OA are discussed.

## Graphical abstract

**Figure:**
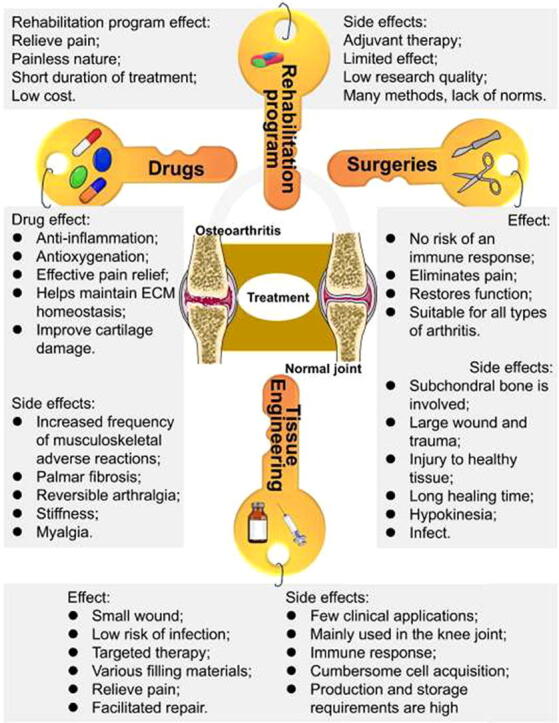

## Introduction

Osteoarthritis (OA) is a synovial joint disease characterized by cartilage degradation, synovial hyperplasia, enhanced vascularity, osteophyte growth, and subchondral bone alterations [1]. Globally, osteoarthritis affected 595 million people in 2020, accounting for 7.6 % of the global population and making it the most prevalent form of arthritis [2]. The disease progression is characterized by articular cartilage degeneration due to aging, overuse, and mechanical stress. Additionally, conditions like obesity, inflammation, and diabetes significantly contribute to OA development [3], [4], [5], [6]. Currently, OA represents a major public health challenge, impacting both individual health and healthcare systems globally. The disease poses substantial health, economic, and social challenges [7]. In response to this growing burden, considerable effort and financial resources have been expended to develop a variety of therapeutic approaches aimed at improving care, quality of life, mobility, mental health, and pain relief for patients with OA [7], [8], [9].

Current therapies for OA primarily aim to alleviate symptoms and modify structural features of the joint. However, no treatment has yet been able to effectively halt or slow the disease's progression or offer enduring symptom relief. Despite efforts, a definitive cure remains elusive. Current treatment approaches can be categorized into three main strategies: basic treatment, medication and surgery. Basic treatment, which encompasses conservative management such as aerobic exercises, weight reduction, strengthening of periarticular muscles, and reduction of joint loading, is indicated for patients with mild lesions and symptoms. However, it offers limited pain relief for patients with stress-line offset OA. Pharmacological interventions, particularly non-steroidal drugs (NSAIDs), possess anti-inflammatory and pain-relieving properties. Both oral and intra-articular injections of NSAIDs can alleviate clinical symptoms, but oral administration may cause adverse reactions, including gastrointestinal issues and liver or kidney damage. Similarly, intra-articular injections of NSAIDs can cause cartilage damage and should not be used repeatedly. Intra-articular injections of sodium hyaluronate and platelet-rich plasma (PRP) enhance joint function and cartilage health through lubricating, yet they provide inadequate pain relief [10], [11]. For structural correction, high osteotomy is primarily used to correct stress line deviations in patients, while those with late-stage OA require joint replacement. Joint replacement surgery is highly effective in alleviating pain and enhancing patients' quality of life [12]. However, it is associated with risks such as infection and prosthesis failure.

The complexity of OA treatment stems from several key factors: (i) the substantial variability in joint structural and clinical presentations of patients, and (ii) the multifactorial nature of the disease, which complicates the search for a universal treatment approach [7], [13], [14]. To address these challenges and provide a comprehensive understanding of OA, this review examines the clinical characteristics, pathogenesis, signaling pathways, evaluation methods, current status of drug therapy, surgical treatment, and novel tissue engineering therapies. The ultimate goal is to advance therapeutic strategies and improve patient outcomes.

## Pathological characteristics and pathogenesis of OA

To advance OA treatment research, a thorough understanding of its pathology and etiology is essential. We examine the pathological characteristics of OA through four key perspectives: patient phenotype, histological score, gene phenotype and protein level changes. This integrated approach offers a comprehensive understanding of disease mechanisms underlying OA.

### Macroscopic characteristics of OA

#### PAIN

OA is characterized by the complex interplay of multiple pathological processes, including oxidative stress, synovial membrane inflammation (synovitis), and immune system dysregulation [15]. Pain, the most prominent symptom, manifests in distinct patterns. Individuals with hip or knee OA typically report two types of pain: intermittent but intense pain, and continuous, milder discomfort [16], [17]. Pain progression typically follows a characteristic pattern. In early-stage OA, pain is often activity-related and predictable, while middle-stage OA is marked by persistent pain, particularly at night. Advanced OA is characterized by more persistent and unpredictable pain episodes. [18]. Furthermore, OA is marked by two distinct types of pain: pain at rest and pain triggered by movement. The temporal distribution of pain shows distinct patterns: while pain is predominantly experienced during physical activities during the day, a significant number of patients also report nocturnal pain, indicative of advanced disease stages [19]. Research indicates that individuals with OA exhibit specific alterations in their somatosensory system, notably heightened sensitivity to mechanical skin stimuli, a phenomenon associated with sensitization due to pathological changes [20].

#### Other characteristics

OA manifests through multiple clinical features beyond pain, including reduced joint mobility, instability, swelling, muscle weakness, and pain-induced psychological distress and chronic fatigue [19]. Previous research has highlighted psychological factors in OA, such as ‘losing face’ due to knee OA, pain anticipation and avoidance, and a vicious cycle of negative emotional experiences. Additionally, social factors, including social and family support, workplace and employment uncertainty, and the built environment, play a significant role in disease experience [21]. Clinical studies have demonstrated that many individuals with knee OA exhibit joint instability, highlighting its role in disease progression [22]. This instability represents a potential therapeutic target for knee OA, as addressing it may improve joint function and slow disease progression. The knee joint is inherently anatomically unstable between the tibial and femoral surfaces, relying on surrounding ligaments and menisci for stability [23]. In addition to cartilage degeneration and mitochondrial dysfunction, genetic background can influence OA characteristics, such as disease incidence and severity [24].

#### Histological scoring system

**Fig. 1:**
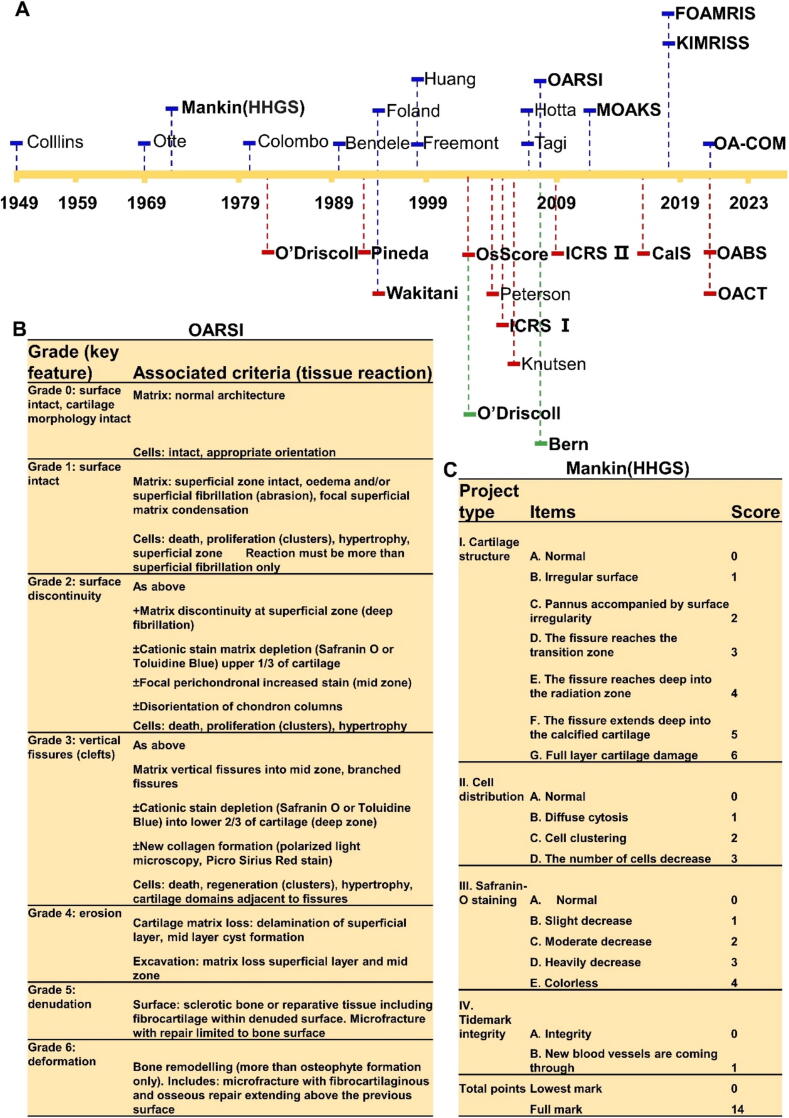
Chronological development of cartilage histological scores after the first macroscopical score in 1949 (Collins). A. Scores for OA are depicted in blue, while those for *in vivo* cartilage repair are in red, and scores for in vitro tissue engineering are shown in green(modified from [49]). B. The Histological-Histochemical Grading System (HHGS) or a score derived from it is predominantly utilized for assessing osteoarthritic cartilage. C. Osteoarthritis Research Society International (OARSI) grading system for osteoarthritis cartilage histopathology is a recognized and valid alternative for assessment. (For interpretation of the references to colour in this figure legend, the reader is referred to the web version of this article.)

The evolution of cartilage histological scoring systems represents a significant advancement in OA assessment. Since Collins and McElligott first scored OA cartilage histology in 1949 [25], the evaluation methods have undergone substantial development (Fig. 1A). The early systems for OA grading were relatively simple, for example, in 1969, the Aitken system only graded the cartilage surface structure [26]. A significant advance occurred in 1971 with the emergence of the Histological-Histochemical Grading System (HHGS), which became a foundation for subsequent scoring systems. Various systems have since adopted and modified HHGS parameters, including the Colombo scoring system [27], which is similar to HHGS, the Benedele scoring system [28] that applies an overall severity score directly to cartilage sections, the Foland scoring system [29] that measures fibrillation, chondrocyte necrosis, cartilage formation, and local cell loss, and the Yagi scoring system [30] that quantitatively assesses matrix consumption and cell numbers. The HHGS system (Fig. 1B) introduced comprehensive evaluation criteria, including ‘cartilage structure’, ‘cell distribution’, ‘safranin O staining’ and ‘tidemark integrity’ as distinct sub-items [31], The sum of the individual scores ranges from 0 (normal) to 14 (severe OA). Despite ongoing debates regarding its reproducibility and validity, the score remains in use due to its overall comprehensive properties, making it a valuable tool for analyzing the characteristics of degenerative cartilage [32], [33], [34], [35].

An important alternative emerged with the Osteoarthritis Research Society International (OARSI) system (Fig. 1C) [36], which seems to be more suitable for inexperienced raters, providing additional information on the surface of the cartilage and the different stages in the scoring [34], [36]. The OARSI system has gained prominence because it appears more robust and reliable than HHGS, so it is preferred in most cases.

Significant technological advances have enhanced scoring capabilities. In 2011, the development of the Magnetic Resonance Imaging (MRI) Osteoarthritis Knee Score (MOAKS) improved the assessment of bone marrow lesions (BML) by providing regional division and cross-regional scoring. It also enhanced cartilage scoring with subregional assessment and refined meniscal morphology scoring by including meniscal hypertrophy, partial maceration, and progressive partial maceration [37], [38], [39]. The reliability for most of the assessed functions was very good to excellent [37]. However, the use of MRI made it costly and not widely applicable. Subsequently, the Knee Inflammation MRI Score (KIMRISS) [40] and the preliminary Foot Osteoarthritis MRI Score (FOAMRIS) [41] were both modifications based on MOAKS. In 2021, the OA-COM (0–54 range) was introduced as a comprehensive, unidimensional, irreversible, and fine-grained MRI-based OA severity score. It integrates regional scores for cartilage, osteophytes, and menisci to provide a holistic assessment of joint pathology [42].

Several specialized scoring systems have emerged for specific applications. Among the numerous scoring systems available for assessing *in vivo* cartilage repair, the International Cartilage Research Society (ICRS II) stands out as the most suitable option. This is due to its validation, comprehensive nature, and the detailed evaluation of each cartilage feature individually [43]. When assessing engineered cartilage outside the body, the Bern score [44] is often the go-to choice. This preference is due to its validation and its ability to accurately differentiate the unique features of tissue-engineered cartilage [45], [46], [47], [48].

After undergoing development and changes, the cartilage histological scoring system has finally formed a pattern in which different tissue types apply specific scoring systems to focus on their tissue specificity. For instance, the scoring system for OA cartilage emphasizes the degenerative aspects of both healthy and diseased tissues. Meanwhile, the *in vivo* cartilage repair scoring system concentrates on assessing the success of cartilage defect repairs. Additionally, the tissue engineering cartilage scoring system evaluates the quality of newly formed cartilage post *in vitro* tissue engineering. The emergence of the cartilage scoring system has solved the difficulty of quantifying cartilage repair, establishing a standard for the field and promoting its growth.

#### Genetic phenotypic abnormalities

Articular cartilage comprises distinct cellular phenotypes, including proliferative chondrocytes (ProCs), pre-proliferative chondrocytes (PreHTCs), and hypertrophic chondrocytes (HTCs), which play critical roles in maintaining joint function. Each subtype exhibits unique characteristics: (i) ProCs, primarily located in the growth plate's proliferation zone, display a distinct gene expression pattern that influences RNA metabolism and stability. (ii) PreHTCs express genes related to biological adhesion and multicellular processes, enabling them to regulate the initiation of proliferation and differentiation. (iii) HTCs highly express genes involved in catabolism and transmembrane transport activities. The specialized functions of these subtypes contribute to cartilage homeostasis. For example, HTCs regulate the mineralization of the cartilage matrix, while ProCs and PreHTCs maintain and regulate other aspects of cartilage biology [50].

The relationship between chondrocyte phenotype and genetics is evident in how genes regulate chondrocytes responses to stimuli, thereby influencing their phenotype and behavior. Recent advances in genetic research have enhanced our understanding of OA pathogenesis. Genome-wide association studies have identified differentially expressed genes (DEGs) associated with OA, as summarized in Table S1. These genetic insights provide crucial information for developing targeted therapeutic strategies. Initial genetic and genomic linkage studies suggest that variations or mutations in genes related to extracellular matrix (ECM) and signaling molecules may dictate OA susceptibility [51], [52]. Styrkarsdottir *et al*. identified rare missense variants in the SMO and IL-11 genes, as well as a common variant in COL11A1, that are significantly associated with hip OA development [53]. Tachmazidou *et al*. [54] found that single-genotype correlations were primarily associated with developmental bone diseases, collagen metabolism, and ECM remodeling. Additionally, the analysis confirmed that 10 genes, including TGF-β1, FGF18, CTSK, and IL-11, could serve as novel therapeutic targets for OA. Cindy *et al.* [55] identified risk-specific genetic variations across all OA phenotypes, including sex-specific risk loci and associations in younger patients. The study identified 11 OA phenotypes with 100 independently associated risk variants, including 52 novel associations. Using a recessive genetic model, they identified a strong association between hip OA and Chondroadherin-like 1 (CHADL1) mutations and confirmed that SOX5 mutations contribute to cartilage destruction in OA and are a significant risk factor for pain symptoms. Additionally, recent years have seen significant advances in understanding the role of epigenetics in OA onset and progression. Undoubtedly, studying the interplay of various factors in OA onset and progression enhances our understanding of its etiology. This knowledge is crucial for identifying novel therapeutic targets and enabling early diagnosis and treatment of OA patients.

#### Protein interactions play a role in OA

Several key proteins and their interactions play critical roles in OA development. The *DVWA* gene, also known as *COL6A4P1*, encodes a protein with a double von Willebrand factor α domain (VWA domain [56]), which mediates cell adhesion and protein–protein interactions [57]. Specific genetic variations, including *DVWA* single nucleotide polymorphisms (SNPs): rs11718863, rs7639618, rs7651842, rs7669807 and rs17040821, reduce the interaction between DVWA protein and β-tubulin, thereby influencing disease progression. This protein–protein interaction may protect joints from OA development [57]. Population-specific effects have been observed that rs11718863 and rs7639618 may affect β-tubulin binding in Asian populations and contribute to OA pathogenesis in Japanese and Chinese populations [57].

Additional protein interactions contribute to OA pathogenesis, such as *Fibrin2* (*FBN2*) gene encodes for a glycoprotein which is essential for the assembly of microfibrils in the ECM, significantly contributing to the early stages of morphogenesis [58]. In OA, the genes inhibitor of DNA binding 3(*ID3*), Hairy and enhancer of split 1 (*HES1*), and AP-1 Transcription Factor Subunit Jun (*JUN*) are particularly active, primarily functioning in protein binding and regulating RNA metabolism [50].

Several key proteins play crucial roles in ECM regulation and joint homeostasis. Syndecan 1 (SDC1) protein is part of the Syndecan family of proteoglycans. The SDC1 protein, interacting with ECM receptors, regulates multiple molecular functions, including cell proliferation, migration, and interactions with the cell matrix [59]. During the initial phase of OA, SDC1 expression increases in articular cartilage, playing a role in the repair of cartilage fibrils [59]. The receptor protein of colony stimulating factor 1 （*CSF1R*） gene encodes a receptor for macrophage colony-stimulating factor, responsible for the primary biological responses to this cytokine [60]. *IGF1* translates into an active peptide that fosters cell growth and is crucial for metabolic processes involving glucose, lipids, proteins, and inorganic salts [60].

Protease activity plays a significant role in OA progression. The *ADAMTS* (a disintegrin and metalloproteinase with thrombospondin motifs) gene encodes a protease enzyme (ADAMTS enzyme) that exhibits specific patterns in OA. This enzyme plays a key role in cartilage matrix degradation, particularly in OA and rheumatoid arthritis. ADAMTS8 levels are significantly elevated in late-stage hip OA, while ADAMTS1,4,5 levels are reduced [61].

Structural proteins and cellular components undergo distinct changes in OA. *ACTG1* encodes gamma-actin 1(Actin γ1), a cytoplasmic protein involved in cell movement and cytoskeletal stability [62]. Dipeptidase 1 (DPEP1), located on chromosome 16, encodes dipeptidyl peptidase 1 and exhibits low expression in OA. The missense SNP rs1126464 in DPEP1 is associated with OA risk: the C allele correlates with reduced OA likelihood, while the G allele increases risk [63].

#### Abnormal expression of collagen in joints

*Tenascin-C (TNC)*, Transforming Growth Factor Beta Induced (*TGFBI*), and Cartilage Acidic Protein 1 (*CRTAC1*) genes are upregulated in advanced chondrocytes (stages 3 and 4), primarily affecting the ECM and collagen degradation [50]. Changes in collagen composition changes are a hallmark of OA progression. The COL5A1 gene encodes the collagen type V alpha 1 chain, producing small-diameter collagen fibers found in ligaments and tendons. Its expression is significantly elevated in OA [59]. OA pathogenesis involves a shift characterized by reduced COL2A1 and an upregulation of COL1A1, both of which are associated with fibrosis. This shift contributes to OA progression by disrupting the balance between destructive cytokines and regulatory factors [62]. The collagen ratio influences bone mineralization, for example, an abnormal COL1A1/COL1A2 ratio impairs mineralization in OA osteoblasts [64]. In advanced OA, the expression of COL4A1, COL4A2, COL8A1, COL10A1 and COL11A1 is markedly reduced in bone marrow mesenchymal stem cells (BMSCs) [64]. The COL11A1 gene encodes a collagen fiber predominantly expressed in cartilage. It forms a heterotrimer with the primary cartilage collagen COL2A1 [53], so it is important for maintaining normal collagen ratios. These collagen changes are direct indicators of OA progression stages.

#### Directly affects cartilage differentiation and bone growth

Multiple genes, including Frizzled Related Protein (FRZB), chromosome 2 open reading frame 82 (C2orf82) and transferrin (TF), regulate early joint development and maintenance. These genes are predominantly expressed in chondrocytes of stages 0 and 1 OA and play key roles in skeletal development and cellular stress responses [50]. SRY-box transcription factor 5 (*Sox5),* a novel signaling factor, correlates with conditions such as back pain and lumbar intervertebral disc degeneration. The inactivation of *Sox5* can lead to bone development defects [58]. The low-frequency missense variant of Interleukin-11 (IL11), NP_000632.1: p. Arg112His, is associated with hip OA. This mutant protein fails to support the survival of osteoclast progenitor cells [53]. Nuclear Receptor Binding SET Domain Protein 1 (NSD1) is H3K36 (lysine 36 histone H3) methyltransferase. The functional target gene of *NSD1*, Sox9 (sex-determining region Y box 9), is a key transcription factor for cartilage differentiation [65]. Sox9 mRNA expression gradually decreases during OA progression. Additionally, *NSD1* regulates *Sox9* expression by regulating *H3K36me1* and *H3K36me2* levels in the *Sox9* promoter region and directly activates hypoxia-inducible factor 1α (*HIF1α*) expression [65]. *HIF1α* directly binds to the *Sox9* promoter, activating its expression and influencing early osteogenesis [65]. Most priority genes in hip OA-related loci are related to cartilage and bone development pathways, including Transforming Growth Factor Alpha (*TGFA*), Runt-related transcription factor 2 (*RUNX2*), Fibroblast growth factor receptor 3 (*FGFR3*), Parathyroid Hormone Like Hormone (*PTHLH*) and *COL12A1*. Additionally, Phosphoinositide-3-Kinase Regulatory Subunit 1 (*PIK3R1*) and Lysine Methyltransferase 2A (*KMT2A*) influence bone and/or cartilage development [66]. Growth Differentiation Factor 5 (*GDF5*) is one of the earliest genes expressed in the fetal interarticular region, contributing to the development of joint tissues such as articular cartilage, synovium, meniscus and ligaments. *GDF5* promotes proliferation through the activity of transcription cofactor Yes-associated protein (Y-ap) following joint surface injury. It also facilitates synovial hyperplasia and migrates to injury sites to aid cartilage repair. Low expression of Matrix Gla protein (MGP) in articular cartilage increases OA burden, potentially leading to increased cartilage calcification [67]. TNF Superfamily Member 11 (*TNFSF11*) encodes the Receptor Activator for Nuclear Factor-κB Ligand (RANKL). TNFSF11-RANKL interaction induces osteoclast differentiation and activation. Elevated TNFSF11 levels are associated with worsening arthritis severity [58]. *RANKL* expression increases significantly in the pericyte zones of the intermediate and deep cartilage layers, as well as in grade 2 cartilage of the superficial zone ECM [68].

#### Factors associated with OA

Multiple transcription factors influence joint development and disease progression. Cut like homeobox (*CUX*), a transcription factor involved in brain neuron differentiation and synapse formation, also regulates joint development [58]. Fos Proto-oncogene (*FOS*)*,* a nuclear protein transcription factor, regulates cell growth, division, differentiation, and apoptosis [60]. *RUNX2*, a runt-related transcription factor, enhances chondrocyte development and is strongly correlated with OA severity [69]. The family of Matrix Metalloproteinase (MMP) inhibitors, known as Tissue Inhibitors of Metalloproteinases (TIMPs), including TIMP1, TIMP2, TIMP3, and TIMP4 [70], exhibit strong binding affinity for MMP13. TIMPs are crucial modulators of MMP13-mediated chondrocyte senescence and cartilage matrix alterations [59]. The expression of *MMP13* [69], [71] and *TIMP1* [59] is significantly increased in OA. The Epidermal Growth Factor Receptor (*EGFR*, also known as Erb-B2 Receptor Tyrosine Kinase 2 *(ERBB2)*) belongs to the family of receptor tyrosine kinases (*RTKs*). *EGFR* possesses inherent tyrosine kinase activity and can be activated by binding to peptide growth factors such as Epidermal Growth Factor (EGF). In advanced-stage of OA, there is a notable increase in *EGFR* activity in human OA samples. *ERBB2*, which lacks a ligand-binding domain, is markedly expressed in synovial cells of rheumatoid arthritis patients [72]. Signal Transducer and Activator of Transcription 1 (*STAT1*) is a key transcription factor in joint inflammation and destruction [72].

#### Genes play a role in OA through multiple pathways

OA pathogenesis involves complex pathway interactions. In OA tissues, the GO pathway, which is significantly up-regulated and linked to the ECM, plays a key role. Conversely, the most notably down-regulated pathways are associated with nutritional and hormonal responses. Within the KEGG pathway analysis, the most significantly up-regulated pathways include those involved in local adhesion, cell adhesion molecules (CAMs), phagosomes, complement systems, and coagulation cascades. Down-regulated pathways include the mitogen-activated protein kinase (MAPK), Forkhead box O (FOXO), hypoxia-inducible factor 1 (HIF-1), tumor necrosis factor (TNF) signaling pathways, and circadian rhythms [73]. Development-related pathways influence OA progression, such as Patched 1 (PTCH1) gene encodes a receptor for Hedgehog (HH) ligands, initiating the HH signaling pathway, which is crucial for regulating chondrocyte proliferation and osteogenesis during endochondral ossification and bone lengthening [58].

Multiple genes participate in inflammatory responses of OA synovium, including Fibronectin 1 (FN1), COL1A1, IGF1, Secreted Phosphoprotein 1 (SPP1), TIMP1, Biglycan (BGN), COL5A1, MMP13, Clusterin (CLU), and SDC1. These genes contribute to various pathways, such as the AMP-activated protein kinase (AMPK) signaling, osteoclast differentiation, insulin signaling, autophagy, ECM-receptor interactions, and the HIF-1 signaling pathway. These processes play a crucial role in the pathogenesis of OA [59]. CD14, functioning as a co-receptor, engages in the signaling pathway initiated by lipopolysaccharide binding, potentially inciting synovial cell inflammation through toll-like receptor activation [60]. COL6A1 and COL6A3 contribute to OA pathogenesis via focal adhesion and ECM receptor interactions [62].

#### By promoting inflammation, the immune system contributes to the onset and progression of OA

IL-6 and IL-8 predominantly orchestrate immune and inflammatory reactions [60]. Transmembrane Immune Signaling Adaptor (TYROBP) plays a role in modulating immune and inflammatory reactions [60]. CD163 acts as a marker for cells of the monocyte/macrophage lineage and functions as a receptor for hemoglobin clearance, exhibiting properties that can both promote and suppress inflammation [63]. In OA-affected cartilage, the expression levels of S100 Calcium Binding Protein A4 (S100A4) and Galectin 1 (LGALS1) are elevated, likely due to the enhanced expression of Pituitary Tumor-Transforming Gene 1 (PTTG1). These proteins, S100A4 and LGALS1 induce inflammatory responses and activate catabolic pathways [73]. Several genes critical for immune system function exhibit reduced expression in advanced OA. These include: POU Class 2 Homeobox Associating Factor 1 (POU2AF1), Immunoglobulin Lambda Locus (IGL), Immunoglobulin J Polypeptide (IGJ), Paired Box5 (PAX5), TNF Receptor Superfamily Member 17 (TNFRSF17), Immunoglobulin Heavy Constant Mu (IGHM), macrophage migration inhibitory factor (MIF), Immunoglobulin Kappa Variable 1–5 (IGKV1-5), TNFSF11 (RANKL), Interleukin Enhancer Binding Factor 2 (ILF2/NFAT), Lymphocyte Transmembrane Adaptor 1 (LAX1), Immunoglobulin Heavy Constant Delta (IGHD), Membrane Spanning 4-Domains A1(MS4A1 (CD20)), Immunoglobulin Kappa Variable 4–1 (IGKV4-1), Fas Apoptotic Inhibitory Molecule 3 (FAIM3), immunoglobulin kappa variable 3–20 (IGKV-3–20), CD79A, CD19, and Immunoglobulin Kappa Constant (IGKC), exhibit reduced expression in the advanced stages of OA [74]. The expression of MCF.2 Cell Line Derived Transforming Sequence Like (MCF2L) in synovial macrophages increases significantly in the presence of the inflammatory cytokine TNF. TNF, along with other pro-inflammatory factors, plays a pivotal role in the degradation of cartilage and the synovitis associated with OA. This suggests that MCF2L is implicated in the inflammatory response pathways of synovial macrophages [75].

#### Induced angiogenesis leads to cartilage structure damage and OA pain

Genes related to angiogenesis and cell viability, such as Kelch Like Family Member 21 (*KLHL21*), Sphingomyelin Synthase 2 (*SGMS2*), Integrin Subunit Alpha 5 (*ITGA5*) and *ID3*, exhibit high expression in OA [50]. Neuropilin 1 (*NRP1*) and Cytoskeleton Regulator RNA (*CYTOR*) are co-expressed as up-regulated genes in OA cartilage. CYTOR modulates NRP1 expression via miRNA-206, which is markedly elevated in human OA chondrocytes. NRP1, functioning as a vascular endothelial growth factor (VEGF) co-receptor and signal transducer, promotes angiogenesis [73].

#### Signaling pathway and pathogenesis of OA

OA is a complex degenerative condition impacting the entire joint structure. Currently, no effective treatments exist to reverse the disease, with surgery being the only option for severe cases. Consequently, comprehending the pathogenesis of OA and its associated signaling pathways is crucial for identifying potential therapeutic strategies.

**Fig. 2:**
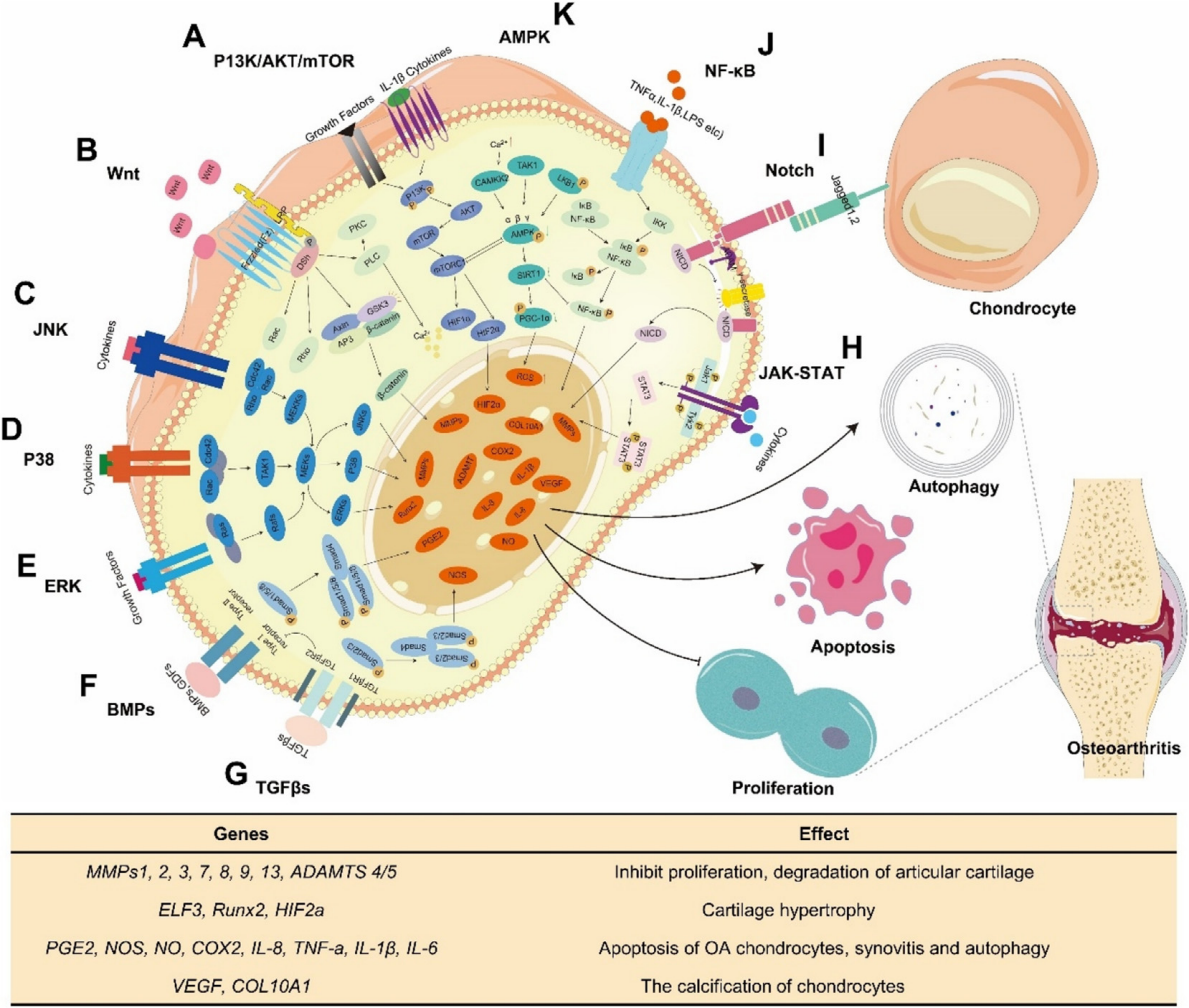
**Signaling pathways associated with osteoarthritis development.** A. P13K/AKT/mTOR pathway. B. Wnt pathway. C. JNK pathway. D. P38 pathway. E. ERK pathway. F. BMPs pathway. G. TGF-β pathway. H. JAK-STAT pathway. I. Notch pathway. J. NF-kB pathway. K. AMPK pathway.

OA is a whole-joint disease characterized by cartilage degeneration, synovitis, subchondral bone sclerosis, and osteophyte formation. Researchers have identified multiple signaling pathways, including PI3K/AKT/mTOR, Notch, NF-kB, Wnt/β-catenin, and MAPK, that contribute to OA progression and are associated with distinct OA phenotypes (Fig. 2).

#### PI3K/AKT/mTOR

PI3K/AKT/mTOR signaling pathway plays an important role in OA, as it is involved in the synthesis of cartilage ECM and is of great significance in maintaining cartilage homeostasis (Fig. 2A). PI3K/AKT/mTOR signaling pathway can negatively regulate chondrocyte apoptosis under various pathological conditions. Activation of this pathway can reduce chondrocyte apoptosis and prevent OA [76]. It can also reduce the inflammatory response by inhibiting protein phosphorylation and promoting autophagy [77].

#### Notch

The Notch signaling pathway plays a multifaceted role in chondrocyte differentiation and the process of endochondral ossification (Fig. 2I). It functions as an inhibitor at the initial stage but becomes stimulatory at the final stage [78], [79]. It can maintain the mesenchymal progenitor cell pool in bone marrow by inhibiting osteoblast differentiation and also participate in the maintenance of cartilage homeostasis [79], [80].

#### NF-κB

The nuclear factor kappa B (NF-κB) signaling pathway is extensively implicated in the pathophysiology of OA, with its activation in chondrocytes occurring during aging and in response to OA-related inflammation, through a range of mechanisms [80], [81] (Fig. 2J). The NF-κB signaling pathway is pivotal in the pro-inflammatory responses of chondrocytes to both extracellular and intracellular injuries. Activation of NF-κB initiates the production of chemokines, cytokines, prostaglandin E2 (PGE2), MMPs 1–13 [82], [83], ADAMTS 4 and 5, as well as angiogenic factors. Consequently, this activation fuels a signaling cascade that results in cartilage degeneration, neovascularization, and synovitis [84]; the NF-κB signaling pathway also plays a central role in regulating the differentiation process of chondrocytes [85]. Sustained activation of NF-κB can drive articular chondrocytes from a prehypertrophic phase to terminal hypertrophy [86]. Furthermore, the NF-κB signaling pathway can induce chondrocyte apoptosis and enhance MMP-2 expression and activation through PGE2, which is involved in IL-1β-induced chondrocyte death [86]. Ultimately, the NF-κB pathway may operate autonomously or engage with BMP, Wnt, and other signaling networks [84], such as NF-κB pathway involved in IL-1b-induced chondrocyte Wnt-5A [87].

The NF-κB signaling pathway is crucial in OA, influencing chondrocyte differentiation, pro-inflammatory stress responses, catabolism, and apoptosis. Sustained activation of NF-κB can drive articular chondrocytes from a prehypertrophic phase to terminal hypertrophy [86]. The pathway in question intensifies cartilage degradation by stimulating the production of catabolic elements and inflammatory mediators. This is achieved through the activation of nitric oxide (NO), cyclooxygenase-2 (COX-2), nitric oxide synthase (NOS), and PGE2, which collectively contribute to joint damage [84]. Impairments in the development of the NF-κB p65 subunit led to growth retardation due to chondrocyte apoptosis, thereby worsening OA [88]. Additionally, NF-κB influences the accumulation and remodeling of ECM proteins, further impacting the disease's progression [85].

#### Wnt/β-catenin

The Wnt/β-catenin signaling pathway is instrumental in cartilage and bone development(Fig. 2B). β-catenin modulates chondrocyte phenotype, maturation, and function through both canonical and non-canonical Wnt pathways, exerting multifaceted regulatory effects on cartilage development, growth, and maintenance [89]. It also influences the lineage specification of bone-derived stem cells/progenitor cells and the differentiation of chondrocytes and osteoblasts [89]. The level of β-catenin signaling dictates whether it promotes or inhibits differentiation; in embryos, low levels encourage chondrogenic differentiation of stem cells, while high levels inhibit this process [90]. Furthermore, the Wnt/β-catenin pathway contributes to inflammation, leading to joint inflammation by upregulating COX-2 expression in articular chondrocytes [91] and fostering the development of OA-like phenotypes [92]. The activity of the Wnt signaling pathway is modulated by various factors, including the active BMP signaling pathway, which suppresses Wnt signaling in developing chondrocytes. This intricate regulation underscores the importance of Wnt/β-catenin signaling in the complex interplay between cartilage, bone, and inflammation in the context of OA [93].

#### MAPK

**Fig. 3:**
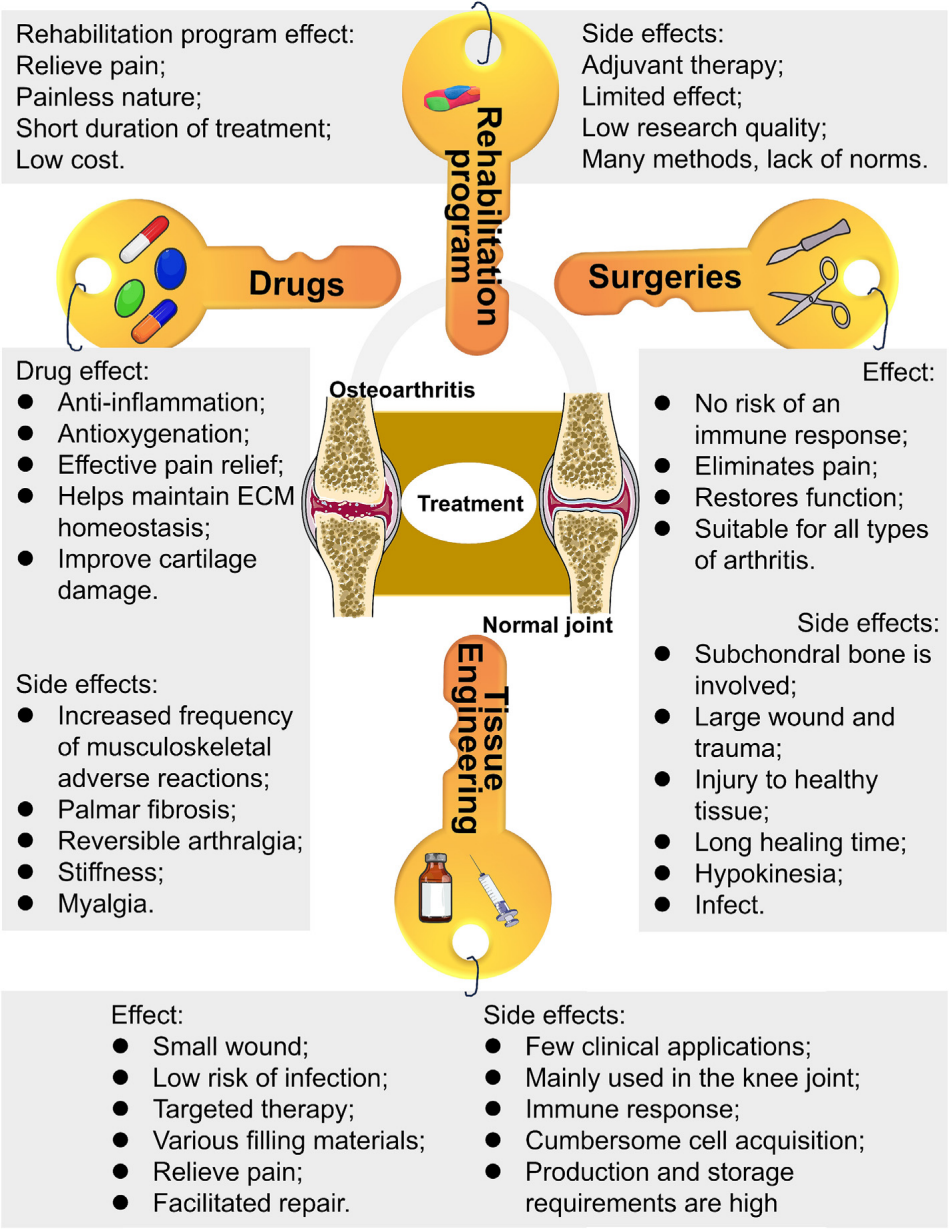
Multiple options for OA treatment.

The MAPK pathway, encompassing ERK, JNK, and p38, is central to regulating inflammation and matrix degradation, processes that lead to the destruction of joint tissues in OA [90](Fig. 3E). Specifically, the p38 MAPK [94] is phosphorylated during cartilage formation, acting as a positive regulator for this process and for chondrocyte differentiation [90]. Moreover, articular cartilage chondrocytes can stimulate the differentiation of subchondral bone osteoblasts via the ERK1/2 pathway, potentially contributing to the abnormal metabolism of these osteoblasts in OA [94]. This highlights the multifaceted role of MAPKs in the pathogenesis of OA, influencing both cartilage and bone metabolism.

The complexity of OA progression, driven by numerous classical signaling pathways, underscores the challenges in its treatment [95], [96], [76] (Fig. 2). However, advances in mechanistic research have provided clearer insights and potential therapeutic strategies for OA (Fig. 3). Clarifying all the signaling pathways involved in OA remains a long-term challenge.

## Advances in the treatment of OA

Current OA treatment strategies include basic therapy (e.g., lifestyle modifications and physical therapy), pharmacological interventions (Table S2), and surgical options (Fig. 3). Emerging tissue engineering approaches, such as stem cell therapy and biomaterials, represent a promising strategy for alleviating and treating OA (Fig. 3).

### Conservative treatment with drugs

#### Painkillers of OA

Pain is a prevalent symptom in OA patients, necessitating the development of effective analgesic medications. Previous studies have found that capsaicin can relieve pain caused by OA. It is an effective agonist of TRPV1 channel, which is a transduction channel found in the nociceptive fibers of the entire peripheral nervous system. Capsaicin alleviates pain by disrupting the peripheral ends of nociceptive fibers, affecting all potential activation pathways of the TRPV1 channel [97]. CRx-102, a novel drug candidate, mitigates OA pain by combining dipyridamole with prednisolone to enhance anti-inflammatory and immunomodulatory effects while avoiding steroid-related side effects [98]. Topical diclofenac significantly reduces pain and morning stiffness in knee OA patients, improves physical performance, and provides relief for sports-related and soft tissue injuries in the ankles, knees, and shoulders [99]. Local administration of cannabidiol can prevent the late-stage development of OA joint pain and nerve injury [100]. Other drugs, such as Boiogito and celecoxib to control the pain of OA. Boiogito is an extract of an herbal mixture to improve knee joint effusion in OA and reduce joint pain [101], while celecoxib is widely used for OA pain control [102].

Common anti-inflammatory analgesics in clinical practice, such as paracetamol (acetaminophen/Tylenol) and aspirin, provide effective pain relief. Non-steroidal anti-inflammatory drugs (NSAID) like diclofenac, loxoprofen sodium, and celecoxib alleviate pain and inflammation by inhibiting COX-1 and COX-2 receptors. However, while inhibiting COX-2 reduces prostaglandin synthesis, alleviating pain and swelling, it can also irritate the gastric mucosa, leading to nausea, vomiting, diarrhea, and other gastrointestinal symptoms. Severe cases may result in gastrointestinal bleeding or elevated transaminases. Prolonged or excessive use can cause liver cell necrosis, fibrosis, cirrhosis, or even liver cancer. Additionally, long-term medication may affect glomerular filtration function, cause renal function damage, or inhibit platelet aggregation, and have a certain impact on bone marrow hematopoietic stem cells. It results in reduced white blood cell counts, increased infection risk, potentially anemia and other severe complications. Muscle relaxants, such as eperisone hydrochloride, relieve pain by relaxing muscles. For more serious arthritis, central analgesics, such as tramadol and oxycodone, can alleviate pain by acting on central nervous system receptors. However, these drugs may cause central nervous system side effects, such as dizziness or dependency. Topical ointments, such as Musk Zhuanggu Ointment and Babu Ointment, also provide therapeutic benefits for arthritis pain. Topical anti-inflammatory and analgesic drugs, such as Voltaren Ointment, Qingpeng Ointment, Shangkeling Spray, and Xueshan Jinluohan, are also effective for localized pain relief.

However, pain is merely a symptomatic manifestation of OA progression. Although pain relief provides the most immediate and direct solution, it cannot address the root cause of OA if implemented without diagnosing joint inflammation or other pathological changes. So these drugs for joint pain management still have several limitations.

#### Anti-inflammatory drugs of OA

Research indicates that highly refined chondroitin sulfate may curb the production of pro-inflammatory cytokines like IL-1 and TNF, along with inflammatory enzymes including Phospholipase A2 (PLA2), COX-2, and nitric oxide synthase 2 (NOS-2). This effect is achieved by limiting the nuclear translocation of NF-κB, which aids in the recovery from OA and lessens inflammation [103]. In a previous study, scientists injected ketamine into the joint, which improved the pathological features of OA and inhibited the inflammatory response [104]. Previous reports have shown that curcumin down-regulates inflammatory factors and improves inflammation [105], while oxcillin A inhibits cell inflammation and hypertrophy to slow down OA [106]. Phospholipase A2 inhibitors combined with biomaterials can reduce inflammation and reduce the occurrence of OA [107]. Glucosamine has a direct anti-inflammatory role, which can alleviate the pain symptoms of OA, improve joint function, and prevent the development of OA [108], [109]. Indomethacin, a frequently utilized NSAID, treats OA [110].

Researchers have investigated anti-inflammatory botanical compounds as potential treatments for OA. For example, gypenosides, key active compounds, inhibit NO and PGE2 synthesis in chondrocytes, reducing inflammation in a dose-dependent manner [111]. Moreover, Kalagana root extract significantly inhibits IL-1β-induced degradation of MMPs, ADAMTS, and ECM in chondrocytes [112], thereby reducing OA severity [113]. Harpagoside, derived from Harpagophytum procumbens, significantly suppresses c-FOS/AP-1 activity in OA chondrocytes, thereby reducing inflammation under pathological conditions [114]. These findings suggest that plant-derived anti-inflammatory compounds may offer promising therapeutic options for OA.

#### Drugs for articular cavity injection

Metabolic imbalances are common in OA, prompting researchers to develop drugs that maintain ECM homeostasis and slow bone and joint degeneration. Currently, there is growing interest in developing intra-articular injectable drugs. Hyaluronic acid enhances proteoglycan synthesis, lubricates joints, improves synovial fluid viscoelasticity, retains water in the joint, and increases cartilage matrix hydration, thereby enhancing resistance to pressure [115]. Betulin protects against OA by inhibiting cartilage ECM degradation and promoting chondrocyte proliferation [116]. Xanthan gum supports the production of sodium hyaluronate and enhances synovial fluid properties under pathological conditions [117]. So Xanthan gum can increase joint mobility and reduce pain [117]. Intra-articular administration of N-acetylphenylalanine glucosamine derivatives slows OA progression and preserves cartilage structural integrity. This treatment reduces abnormalities in the lateral and medial femoral condyles and the tibial plateau [118].

In patients afflicted with OA and rheumatoid arthritis, the articular cartilage exhibits a reduction in proteoglycan and glycosaminoglycan (GAG) content [119]. Iminocyclol promotes the buildup of GAGs in human articular chondrocytes, cartilage, and chondrosarcoma cell cultures. This enhancement could potentially prevent or reverse the degeneration of cartilage in individuals suffering from OA [119]. Phosphate citrate exerts its disease-modifying effect on OA and stimulates the expression of genes related to cartilage protection and ECM production, thereby improving the progression of OA [120]. Progranulin inhibits the degradation of cartilage matrix and reduces inflammation [121]. The medication prevents OA by modulating the TNF-α [122] and β-catenin signaling pathways [121]. Scientists have discovered that microRNA-140 (miRNA-140) is uniquely present in cartilage and controls the enzymes that break down the ECM [123], [124], [125]. Injecting miRNA-140 into the joint can slow the progression of OA by managing ECM balance [123]. Morroniside protects the cartilage matrix from degradation by increasing collagen type II levels and inhibiting chondrocyte pyroptosis [126].

#### Cartilage protective agents

Cartilage damage is a primary therapeutic target in OA, and many drugs are being developed to address it through diverse mechanisms. For example, mitromycin A can alleviate the catabolism of chondrocytes by inhibiting the expression of HIF-2α [127]. Engeletin exerts its protective effect by activating the Nrf2 pathway and suppressing the NF-κB and MAPK pathways [128]. Dihydroartemisinin reduces sclerostin inhibition by reducing the expression of MMP-13 and VEGF in articular cartilage and reducing Leukemia inhibitory factor (LIF) secretion of osteoclasts [129]. Strontium ranelate can improve the quality of cartilage matrix and the chondrocytes viability, thereby reducing articular cartilage degradation [130]. Metformin not only alleviates pain but also safeguards cartilage health. Furthermore, its combination with celecoxib demonstrates superior efficacy in mitigating cartilage damage compared to metformin used in isolation [102]. TD-198946, a derivative of thiophene indazole, promotes cartilage differentiation without inducing hypertrophy. When administered directly to the joint space, this compound is effective in both preventing and repairing the degeneration of articular cartilage [131]. Isoimperatorin mitigates pathological changes associated with OA by retarding the degeneration of chondrocytes, stimulating autophagy, and suppressing mTORC1 activity. This compound holds promise as a therapeutic agent for decelerating the degradation of articular cartilage and treating OA [132]. Eggplant yellow fruit extract restores collagen-proteoglycan and significantly inhibits cartilage destruction by regulating the expression of related genes, which can enhance chondrocyte proliferation and prevent articular cartilage damage, thereby improving OA [133]. The Mongolian medicinal herb known as blue thorn head has been found to enhance cartilage repair in OA and to stimulate chondrocyte proliferation, thus playing a beneficial role in both the prevention and treatment of OA. Additionally, chitosan oligosaccharides have shown the ability to ameliorate cartilage damage, positioning them as a novel biological agent for OA management. Their efficacy is believed to be associated with the modulation of osteoprotegerin and the receptor activator of NF-κB ligand expression. These substances may offer innovative therapeutic strategies for OA, targeting key molecular pathways involved in OA progression [134].

#### Antioxidant drugs

OA development is influenced by various elements, such as oxidative stress and an overabundance of reactive oxygen species. Targeting oxidative stress represents a promising new direction in OA treatment. Rebamipide, Danshen and C-phycocyanin can reduce cartilage degeneration by inhibiting oxidative damage [135], [136], [137]. Ginkgolides possess anti-inflammatory and antioxidant properties, which can notably mitigate inflammation and protect against ECM degradation [138]. Additionally, ginkgolides modulate the AMPK/SIRT1 pathway, reduce mTOR levels, and promote autophagy, thereby protecting chondrocytes from damage [138].

#### Other drugs

In OA treatment, drugs with multiple therapeutic functions can reduce dosage requirements and minimize side effects associated with polypharmacy. Previous studies have found that paeonol has anti-osteoarthritis effect and anti-inflammatory and anti-degradation effects on OA chondrocytes. Paeonol can antagonize the degradation and degeneration of OA articular cartilage *in vivo* and delay the progress of OA. The ERRγ inhibitor GSK5182 mitigates inflammation, cartilage erosion, osteophyte formation, and subchondral bone sclerosis [139]. Danzikangxi granule is effective in slowing down the degeneration of knee joint cartilage in OA and in curbing the abnormal reconstruction of subchondral bone. Additionally, it lessens inflammation. Meanwhile, Tributyryl N-acetyl-D-galactosamine analogues (3,4,6-O-Bu3GalNAc) stimulate chondrogenesis and mitigate inflammation by modulating the Wnt/β-catenin signaling pathway [140]. Chlorophosphonates shield cartilage from harm, curb synovial overgrowth and proteoglycan depletion, and alleviate joint inflammation, swelling, and osteophyte development [141]. The sustained-release thermal gel formulation of Flurbiprofen provides ongoing anti-inflammatory and pain-relieving benefits within the joint space, effectively alleviating symptoms of OA [142].

In addition, some traditional Chinese medicines also have a certain therapeutic effect on OA. For example, Ganjiang Lingzhu Decoction can treat knee cartilage injury to a certain extent through its ability to reduce the expression levels of MMP-3 and TGF-β1 in knee joint effusion The extract has shown to improve OA.

Studies have found that some drugs treat OA by inhibiting chondrocyte hypertrophy. For instance, KAG-308, an oral EP4 selective agonist, curbs the progression of OA by preventing chondrocyte hypertrophy and synovitis [143]. Phloretin curbs the synthesis of pro-inflammatory cytokines like TNF-α, IL-6, IL-1β, and IL-17, while boosting aggrecan and collagen-II levels. This action prevents cartilage breakdown and bone erosion, significantly diminishes joint inflammation, and mitigates synovitis [144].

#### Drug side effects

**Fig. 4:**
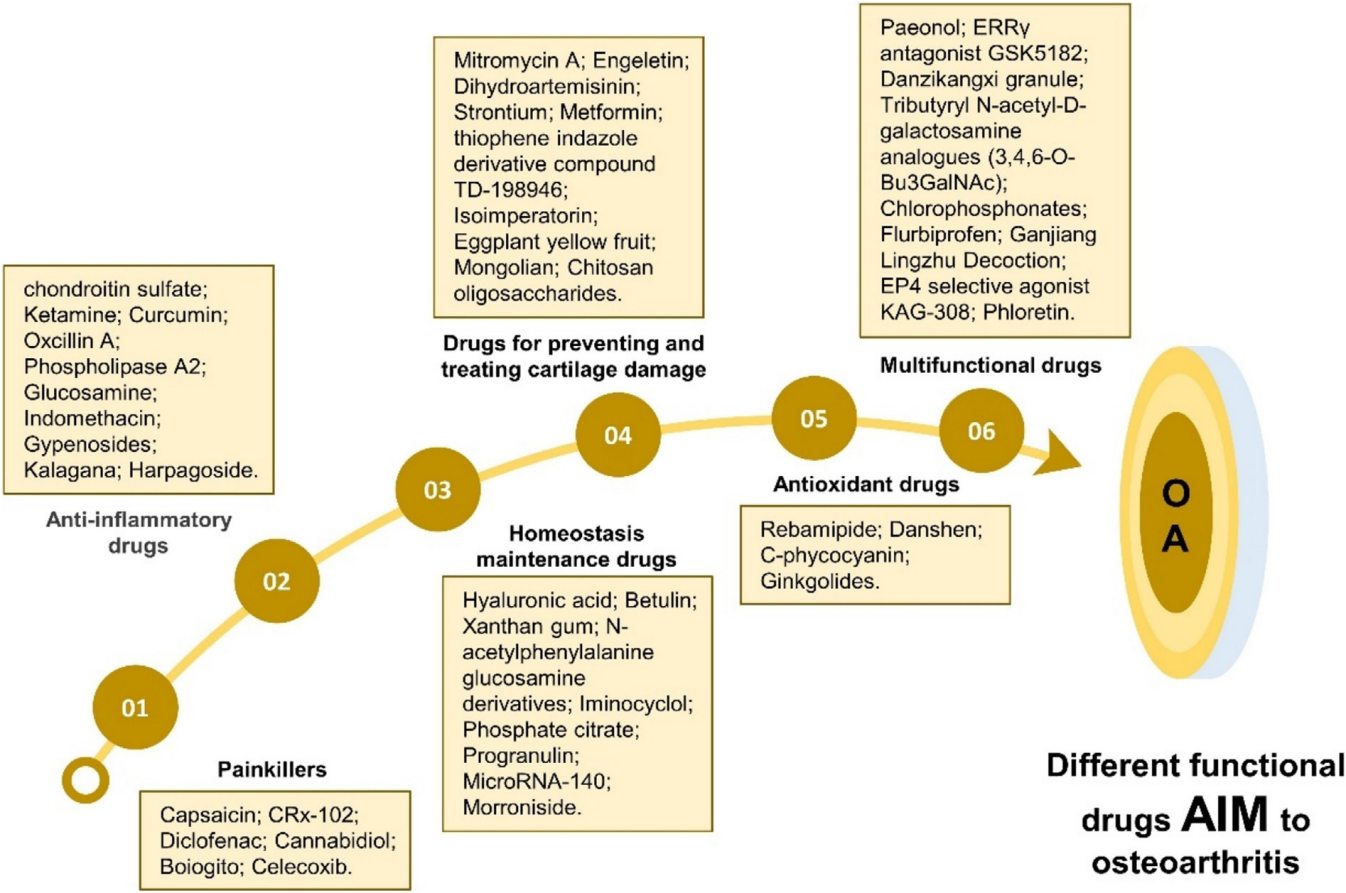
**Drugs to treat osteoarthritis.** The single function of drugs may be the biggest limitation of curing OA.

The matrix metalloproteinase inhibitor PG-116800 has been associated with significant side effects, including musculoskeletal adverse reactions, palm fibrosis, reversible joint pain, stiffness, and myalgia [97]. Gefitinib inhibits EGFR phosphorylation and activates the p38 MAPK pathway, increasing MMP-13 expression, degrading COL-II, and elevating inflammatory cytokine levels in cartilage, thereby exacerbating cartilage damage and inflammation. MicroRNA-34a-5p contributes to joint destruction during OA [137]. These adverse effects limit the suitability of many drugs for OA treatment. Despite the availability of numerous OA drugs, their side effects restrict their clinical utility (Fig. 3, Fig. 4).

### Surgeries

#### Arthroscopic debridement surgery

**Fig. 5:**
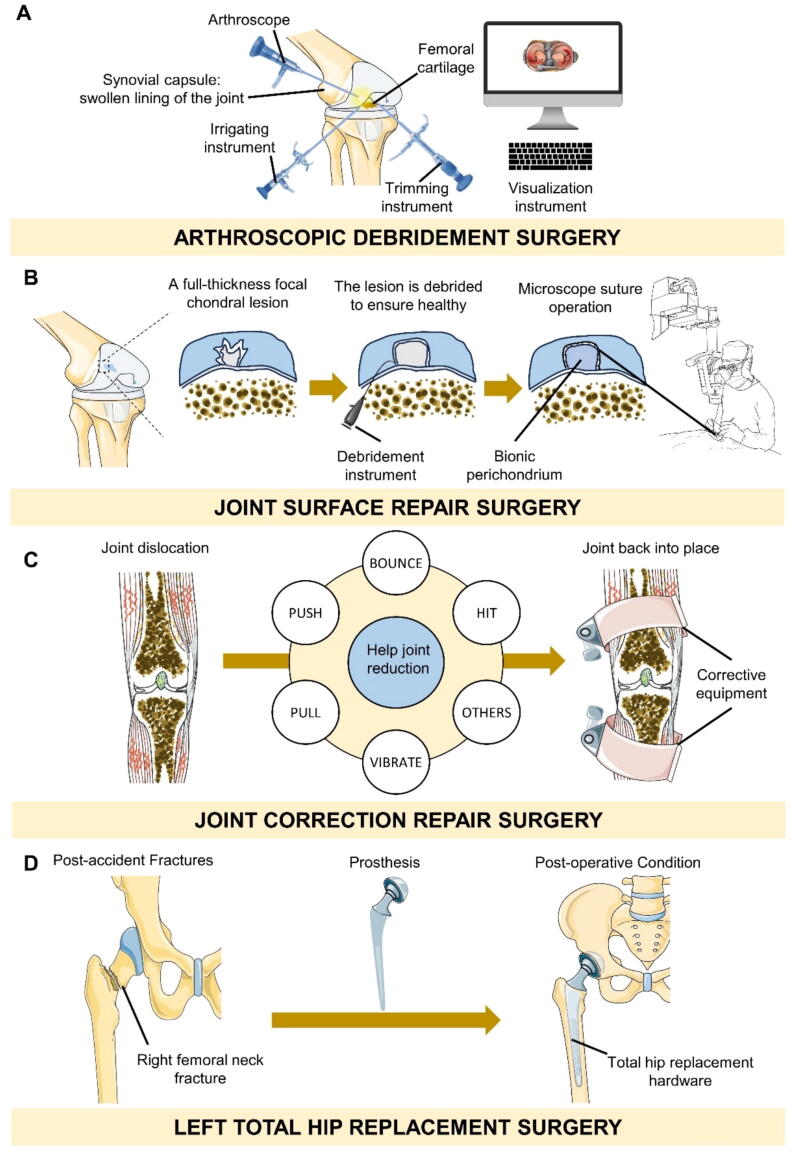
Surgery to treat osteoarthritis. A. Arthroscopic debridement surgery; B. Joint surface repair surgery. C. Joint correction repair surgery. D. Replacement surgery.

Arthroscopic debridement is the most common orthopedic surgery [145], The knee is typically the joint most often targeted for treatment, as depicted in Fig. 5A. In cases where pharmaceutical interventions prove insufficient for knee OA pain, arthroscopic lavage or bridging is commonly suggested [145]. During the surgery, at least 10 L of liquid are used to rinse the joint. The procedure includes the abrasion of uneven articular cartilage for chondroplasty, the removal of any loose fragments, the trimming of cartilage pieces, and the smoothing of the residual meniscus. The goal is to create a stable and robust edge without the need for abrasive plasty or microfracture techniques [145]. Usually, the tibial spine area will be blocked to completely stretch the bone spurs shaved. Although arthroscopic debridement of knee arthritis is controversial, this surgery is still one of the most used treatments to correct the patient 's mechanical abnormalities. Arthroscopic knee debridement [146] can effectively alleviate symptoms and achieve sustained benefits for patients with early degenerative knee joints, particularly those with obvious symptoms who have failed conservative treatment. This treatment shows promise for patients experiencing mechanical symptoms due to degenerative meniscus tears or cartilage flaps, as well as those with symptomatic patellofemoral OA.

#### Autologous transplant for joint surface repair

In bone and cartilage defects, spontaneous repair is limited to the formation of fibrocartilage scars and occurs only when the defect reaches the subchondral bone. However, spontaneous cartilage repair is sluggish and insufficient, attributed to the absence of molecular components, intricate structure, rigidity, and resilience found in natural articular cartilage [147]. Due to the limitations of spontaneous repair, a therapeutic approach known as joint preservation has been developed [148]. Developed techniques in this field include chondrocytes and mesenchymal stem cells (MSCs) transplantation, utilizing grafts from the periosteum and perichondrium, synthetic materials, and growth factors to encourage the development of new joint surfaces [149]. Examples of such procedures are osteochondral autograft transplantation (OAT) and autologous chondrocyte transplantation (ACT), as shown in Fig. 5B [150].

#### Osteochondral autograft transplantation (OAT)

OAT has proven to be an effective strategy for halting the progression of isolated patellofemoral osteoarthritis (PFOA) [151], [152]. While a universal agreement on the optimal treatment of patellofemoral lesions with OAT has not been reached, the procedure is still considered a feasible choice for treating smaller lesions that extend to the underlying subchondral bone [151]. OAT is capable of addressing focal cartilage damage that is characteristic of OA. A significant benefit of employing OAT is the absence of the risk for an immune response. Studies have indicated that OAT tends to offer longer-lasting results and enhanced durability in patients with high demand on their joints, as well as better overall outcomes [153], [151]. This suggests that OAT is a valuable consideration for managing cartilage defects in the context of patellofemoral OA.

#### Autologous chondrocyte transplantation (ACT)

ACT postpones the necessity for total joint arthroplasty, particularly for younger individuals with early-stage OA. These patients, who often have high functional expectations and anticipate a long, active life, can benefit from ACT to maintain their activity levels. ACT may result in an improved quality of life [154]. Messina *et al*. [155] initially performed autologous chondrocell transplantation at the carpometacarpal joint of the thumb, and implanted autologous chondrocells by open or arthroscopic technique for carpometacarpal joint replacement to achieve pain relief, restore function, delay more aggressive treatment, and ultimately slow down the development of OA.

In addition to joint-sparing surgical treatments, adjunctive therapies such as physiotherapy and brace correction play a supportive role in OA management.

### OA's rehabilitation program

#### Physiotherapy for OA

Physical therapy plays a crucial role in the management of OA, has its unique features in alleviating pain, relieving OA symptoms, correcting deformity and promoting functional recovery. Physical therapy is increasingly used in clinical settings due to its few side effects, painless nature, short duration of treatment, and low cost. Common physical therapy methods currently used for OA include exercise therapy, ultrasound therapy [156], [157], extracorporeal shockwave therapy (ESW) [158], [159], [160], electrotherapy [161], [162], [163], pulsed electromagnetic field therapy (PEMF) [164], [165], [166], and whole-body vibration therapy (WBV) [167], [168]. Exercise therapy is a major component of the OA rehabilitation treatment plan, playing a primary role in enhancing muscle strength and overall endurance, maintaining or restoring the range of joint motion, improving joint function, and preventing and alleviating osteoporosis [169], [170], [171], [172]. Other therapies aim to promote local blood circulation and reduce inflammatory responses, thereby achieving the goal of alleviating joint pain.

### OA rehabilitation aid

#### Orthopedic insoles of OA rehabilitation assistive devices

Orthopedic insole, particularly wedge-shaped ones, can reduce the internal adduction angle and external torque of the knee joint, providing a certain corrective effect on foot valgus. To a certain extent, they can alleviate patients' pain by reducing the stress on the medial side of the knee joint, unstable medial compartment alignment or increased pressure is an important indication for the use of orthotic shoe inserts in patients with knee osteoarthritis (KOA) [173], [174]. From the OARSI guidelines, we find that conditional recommendations are given for the use of orthotic shoe inserts [175].

#### OA rehabilitation aids are used for joint correction repair

Knee joint deformity cannot be restored by natural recovery and must be corrected by surgery [176]. There are many reasons for knee joint deformation. OA and rheumatoid arthritis are the primary clinical causes [177]. Once the knee cartilage is damaged, the joint space will narrow and the line of force of the lower limb will change [178]. Changes in the lower limb alignment will aggravate the knee joint deformity, which cannot be restored by drugs and rehabilitation therapy, necessitating surgery correction [179] (Fig. 5C). OA of the medial compartment of the knee can be effectively addressed through high tibial osteotomy (HTO), a well-established surgical procedure [180], including open wedge, closed wedge, oblique osteotomy, and ball and fossa osteotomy, which tends to be used in more active patients, for which Tomofix plates are the most common fixation plates [181]. Omori *et al*. [182] conducted a closed wedge osteotomy to address medial compartment knee OA with preoperative Kellgren-Lawrence (KL) grades ranging from II to IV. The osteotomy site was then stabilized using two screw pins and the eight-line technique, which is a method used for the treatment of knee OA [180].

Wearing knee orthoses can alleviate patient pain to a certain extent and improve the overall level of functional activity by adjusting biomechanical risk factors [183]. Each type of orthosis targets different alignments or deformities, and it is recommended that patients customize their knee orthoses based on their preferences and assessment results [184]. The main types currently applied to KOA patients include varus-valgus orthoses, patellofemoral orthoses, tibiofemoral orthoses, and general knee joint supports, *etc.* [185]. The use of assistive devices can also relieve KOA patient pain and enhance overall functional activity levels, significantly increasing the walking distance in the 6-minute walk test [186], [187], [188]. Previous guidelines have strongly recommended both orthoses and assistive devices [175], [189].

### OA regeneration rehabilitation therapy

Multiple lines of evidence indicate that PRP maintains satisfactory analgesic and functional activity levels in follow-ups at 6 months and 1 year after treatment, with effects superior to other intra-articular injections [190], [191], [192]. Although the specific components at play and their mechanisms are not yet elucidated, leukocyte-poor PRP has been shown to be more effective [190]. This may suggest that we need to consider patient preferences in clinical applications. In addition to PRP, stem cell intra-articular injections can alleviate patient pain, increase the overall thickness of OA cartilage, and enhance overall functional activity levels [193], [194]. However, due to the lack of definitive evidence supporting the safety and efficacy of this regenerative rehabilitation technology, and the inability to standardize stem cell sources, mechanisms of action, and preparation processes, clinical studies need to consider the safety and reliability of its clinical application, promoting its standardized use in clinical settings [194], [195].

#### Surgeries-Joint replacement

Knee arthroplasty [196], [197] involves the removal of damaged joint surfaces that the body is unable to repair, and their partial replacement with artificial joints. The objective of this surgical intervention is to address joint deformities, alleviate pain in the knee, preserve the stability of the joint, and enhance the functionality of the knee joint (Fig. 5D). Total joint replacement surgery effectively treats severe joint conditions., including a variety of knee joint inflammatory arthritis such as rheumatoid arthritis [198], osteoarthritis [199], [200], hemophilia arthritis [201], [202], traumatic arthritis [203], [204], OA after failed high femoral osteotomy [205], [206], patellar arthritis in the elderly [207], [208], [209], resting infectious arthritis (including tuberculosis) [210], [211], a small number of primary or secondary osteochondral necrotic diseases [212], [213], [214], at present, the joints that can be replaced clinically include hip joint [215], knee joint [197], [216], [217], humeral head [218], [219], [220], elbow joint [221], [222], vertebral body [223], [224], [225], [226], pelvis [227], [228], [229], scaphoid [230], [231], [232], [233], lunate [234], [235] and distal radius [236], [237], [238], which are commonly used in hip joint and knee joint. The most common sequelae of arthroplasty are inflammatory infection, which can lead to inflammatory diseases such as osteomyelitis or suppurative arthritis in severe cases [239], [240], [241], [242].

### Patellofemoral arthroplasty (PFA)

Anterior knee pain is frequently attributed to isolated patellofemoral arthritis. Among individuals aged 40 and above, the incidence is approximately 9 %. This index escalates to over 30 % for those aged 60 and older [243]. Avon prosthesis is a patellar femoral prosthesis. Avon patellofemoral joint replacement [244] provides predictable good results and good medium-term survival rate for isolated patellofemoral arthritis. It is better to perform the operation before or even in the early stage of lateral ventricular arthritis. It is relatively easier for patellofemoral arthroplasty (PFA) to be followed by revision to total knee arthroplasty (TKA). The indications of PFA include advanced patellofemoral arthritis, post-traumatic arthritis (PTA), advanced patellar or trochlear chondromalacia or both, tibial femoral Ahlbäck score ≤ 1 and severe symptoms that significantly impact daily activities [245]. PFA is contraindicated in cases of tibiofemoral OA, severe chondromalacia, calcinosis, systemic inflammatory conditions, complex regional pain syndrome, and in the presence of infection [245].

### Total knee arthroplasty (TKA)

TKA is a predictable and lasting method for the treatment of end-stage patellofemoral arthritis [246]. TKA is an effective method for different degrees of preoperative deformity, patellar subluxation and patellar size [246]. Previous studies have shown that the success rate of TKA in patients under 45 years old is comparable to that of TKA in the elderly [247]. Although TKA is still insufficient for all knee joint disease, it has been clinically considered as a successful and long-lasting treatment option. Meanwhile, TKA surgical alternatives for patients with end-stage degenerative joint disease of patellofemoral joint include arthroscopic debridement, tibial tubercle elevation and translation [248].

### Total hip arthroplasty (THA)

Total hip arthroplasty (THA) [249], [250] stands out as a highly cost-effective intervention in orthopedic surgery, regularly yielding successful outcomes. For individuals with advanced degenerative hip OA, THA consistently offers substantial benefits, including effective pain alleviation, improved functionality, and enhanced overall quality of life. Despite the short-term efficacy of THA and TKA in pain relief and functional restoration, there are certain enduring constraints that can impact the long-term quality of life for patients. It is crucial to acknowledge these potential limitations in the context of THA and TKA procedures.

When conventional methods are ineffective or unsuitable for OA, researchers have explored innovative approaches, such as tissue engineering, to treat the condition. Tissue engineering strategies, including stem cell therapy, 3D bioprinting, and biomaterials, aim to regenerate damaged cartilage and restore joint function. These methods address the root causes of OA, offering the potential for long-term solutions rather than symptomatic relief. As research advances, tissue engineering holds promise for revolutionizing OA treatment and significantly improving patient outcomes.

### Tissue engineering for OA treatment

Cartilage tissue engineering is a dynamic field focused on repairing damaged cartilage through the integration of autologous cells with biomaterials [251], [252]. This approach leverages the selection of suitable seed cells, such as chondrocytes, and the design of biomaterial scaffolds to facilitate cell growth and differentiation [253]. The scaffolds provide a 3D environment that is crucial for cell survival, nutrient acquisition, waste removal, and material exchange, which are essential for cartilage regeneration [254], [255], [256] (Table S3). As the biological materials degrade, the implanted cells proliferate, contributing to the repair of tissue defects. This process harnesses the body's innate regenerative potential, offering a promising alternative to traditional cartilage repair methods [257], [258]. Previous studies highlight the importance of biomaterial scaffolds in providing a conducive environment for cell growth and the potential of autologous cell therapies in repairing cartilage defects [259].

### Stem cells therapy for OA

Current novel approaches for treating OA caused by cartilage damage include cell implantation, cell injection, and scaffold-based therapies.

Researchers implanted the cells into cartilage injuries, demonstrating effective repair outcomes. Shimomura *et al*. employed a scaffold-free tissue engineering construct (TEC), originating from synovial MSCs, for repairing cartilage [260]. The TEC comprises undifferentiated MSCs from synovial sources, enveloped by an ECM exclusively produced by these cells [261]. TEC is abundant in fibrous collagen types I and III, along with adhesion molecules like fibronectin and vitreous connexion [260]. Subchondral bone edema was observed around the TEC implant site at 6 and 24 weeks postoperatively, but these abnormalities resolved by 48 weeks [260]. All patients had a defect filling rate of 100 % within 48 weeks, and no repair tissue hypertrophy was found [260]. Chahal *et al*. injected BMSCs into arthritis knee joint, resulting in reduced macrophage levels and inflammatory markers, thereby alleviating synovial inflammation [262]. Bastos *et al*. conducted a comparison study, evaluating the efficacy of intra-articular injections of cultured MSCs with and without the addition of PRP against corticosteroid injections for the management of OA [263]. The study included 47 patients divided into three groups receiving intra-articular injections. Group 1 (n = 16) received autologous bone marrow-derived MSCs, Group 2 (n = 14) received MSCs combined with PRP, and Group 3 (n = 17) received corticosteroids. Outcome measures included the Knee injury and Osteoarthritis Outcome Score (KOOS), range of motion (ROM), and cytokine levels. The study found that MSC-treated groups showed the most significant improvements in KOOS scores. The data demonstrated that intra-articular injections of culture-expanded MSCs derived from bone marrow can significantly enhance functional outcomes and alleviate OA symptoms [263].

**Fig. 6:**
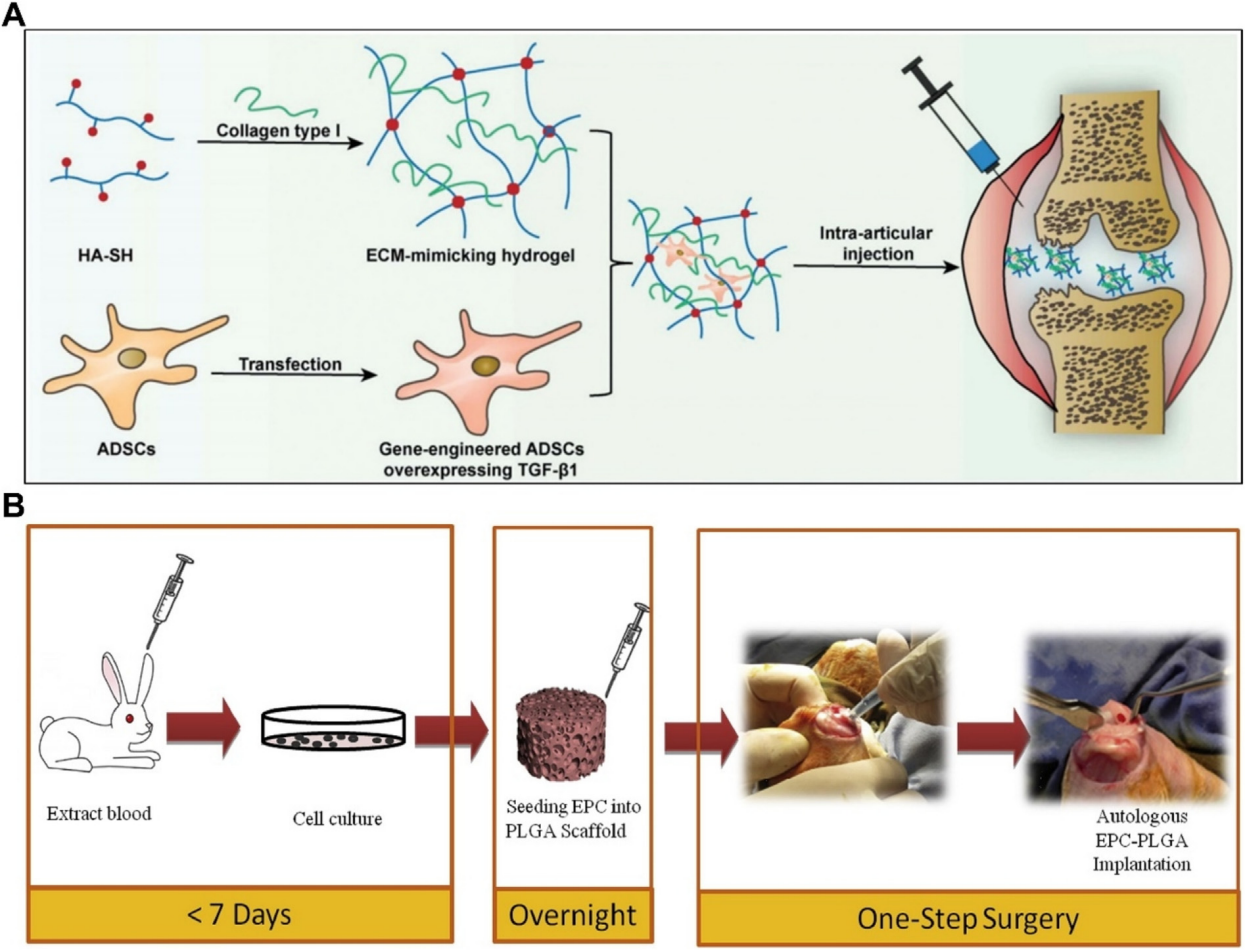
Scaffold loaded cell strategies of tissue engineering in cartilage repair and OA treatment.

Research has shown that various types of stem cells can effectively treat arthritis resulting from cartilage damage in joints. Among these, MSCs are multifunctional cells with robust self-renewal capabilities. Currently, MSCs used in research are primarily derived from bone marrow and adipose tissue (AT), but can also be sourced from other tissues, including endothelium, meniscus, and amniotic membrane [264], [265]. MSCs leverage their proliferative and differentiation capabilities to form new chondrocytes and repair damaged joint tissues [264], [265], [266], [267], [268]. Several studies have indicated that implanting differentiating stem cells onto scaffolds can enhance the repair of bone and cartilage injuries (Fig. 6). Kim *et al.* implanted MSCs with fibrin glue scaffolds in OA knee patients. Clinical examination and secondary arthroscopy revealed that 58 % of the fibrin glue scaffold group showed normal cartilage, significantly improved compared to 23 % in the scaffold-free group. This study is among the first to use fibrin glue scaffolds for BMSC implantation in OA knees [269]. Grigolo *et al*. implanted BMSCs into a hyAF-11 scaffold (HYAF-11) at the knee joint of OA model rabbits. 3/6 months after surgery, HYAF-11 (HA) group showed significant therapeutic potential for OA [270]. Yu *et al*. developed a hydrogel-based method to deliver genetically engineered ADSCs into joints (Fig. 6A). At 4 weeks post-treatment, the Gel + T-ADSC group exhibited 28.6 % and 42.1 % more bone trabeculae than the PBS group, outperforming other treatment groups [271]. This approach offers a promising biomaterial strategy for improving OA therapy [271]. Bhattacharjee *et al*. developed a minimally invasive injection system to deliver ADSCs on amniotic membrane (AM) for OA treatment. At 14 and 21 days post-treatment, the AM-ADSC group showed reduced knee swelling and joint diameter compared to other groups [272]. Li *et al*. injected allogeneic ADSC and xanthan gum (XG) into the joint for arthritis treatment [273]. At 8 weeks post-surgery, the XG-ADSC-S group showed significantly improved weight-bearing capacity and relatively normal articular cartilage, while the ADSC and XG groups exhibited moderate cartilage degeneration [273]. Desando *et al.* compared BMC and MSC transplantation into a Hyaff-11 (HA) scaffold for meniscal repair in a sheep OA model. At 12 weeks post-surgery, the MSC-HA group exhibited high levels of collagen type I and II in the posterior meniscus, indicating effective meniscal tissue regeneration and supporting cartilage, meniscus, and synovium repair [274].

### Other cells therapy for OA

In addition to the above studies, there are many researchers load stem cells on the scaffold for OA treatment, which is widely used because of its own excellent characteristics, that is, it can differentiate into chondrocytes at the joint. Of course, in addition to stem cells, there are many other types of cells used in the process of cartilage repair. Such as Chang *et al.* conducted a study where they implanted a polylactic acid glycolate (PLGA) scaffold seeded with autologous endothelial progenitor cells (EPCs) into the osteochondral defect of the medial femoral condyle in rabbits [275](Fig. 6B). The PLGA scaffold, when loaded with EPCs, was able to create a microenvironment conducive to osteochondral regeneration without the need for additional growth factors [276]. Hollander *et al*. implanted the esterified Hyalograft C at the patient's knee injury after 14 days of chondrocyte culture. The study results showed that cartilage regeneration was observed in 10 of the 23 patients for 11 months after implantation. The repair tissue of knee biopsy was mature hyaline cartilage [277]. Haghighi *et al*. designed a scaffold with a weight to volume ratio of gelatin, chitosan and fibroin of 2:2:3 (w/v). *In vivo* verification by rabbits showed that 65 ± 9.1 % cartilage tissue was regenerated in the defect of CGS 2:2:3 scaffold with seed chondrocytes, and most of the cartilage tissue was hyaline cartilage [278]. Only 1.6 ± 0.5 % cartilage tissue was regenerated in the defect of CGS scaffold without cells, while no new cartilage tissue was found in the control group [278]. Gobbi *et al*. implanted autologous chondrocytes into a hyaluronic acid scaffold to repair the damaged joint surface of the patellofemoral joint [279]. 24 months post-operative follow-up showed that International Knee Documentation Committee (IKDC) scores of all patients improved compared with baseline. There were 14 cases of grade A (43.8 %), 15 cases of grade B (46.9 %), and 3 cases of grade C (9.4 %). The results indicated that autologous chondrocyte implantation of biodegradable scaffolds was a feasible method for the treatment of cartilage lesions [280].

As tissue engineering technology becomes more and more mature, the stent-supported cell strategy is increasingly favored by clinical researchers. However, cell culture and the survival rate of cells in scaffolds remain challenges. In addition, transportation, storage, cost and other problems of composite scaffolds after stent-supported cells are also obstacles to the clinical promotion of this strategy.

### Materials of tissue engineering for OA treatment

**Fig. 7:**
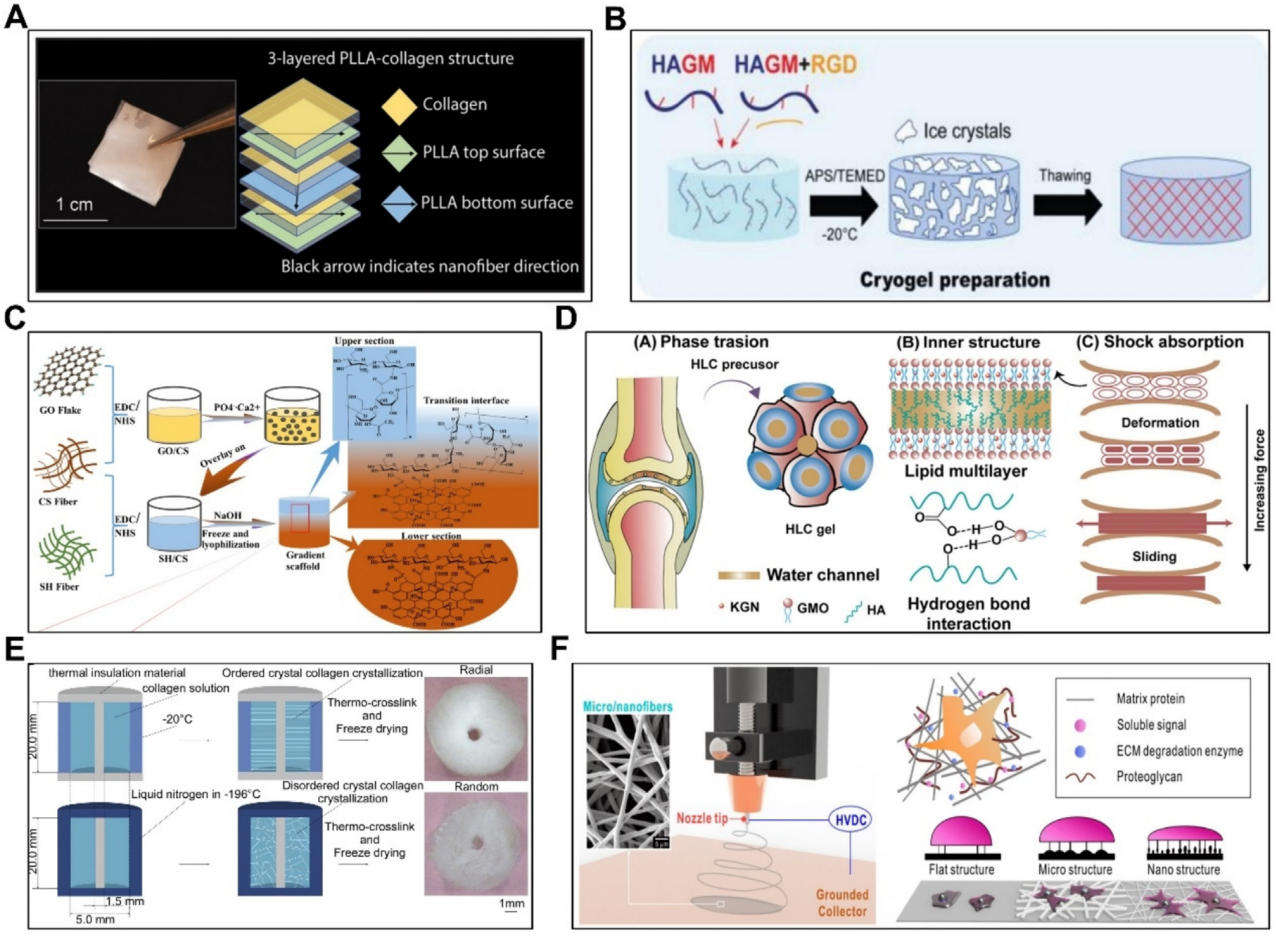
Support material and design strategies of tissue engineering in cartilage repair and OA treatment.

The selection of materials is critical in tissue regeneration and repair, as different tissues require materials with specific properties. For example, materials used in skin tissue should exhibit excellent biocompatibility, mechanical strength, and antibacterial properties. Therefore, the development of multifunctional materials has become a prominent focus in clinical research. In recent years, materials such as polyesters, hyaluronic acid, collagen, and extracellular matrix (ECM) have been widely utilized in OA treatment (Fig. 7).

Polyester materials such as polylactic acid and polycaprolactone are commonly used in OA treatment due to their slow biodegradation and hydrophobicity properties. Liu *et al.* fabricated a biodegradable PLLA nanofiber scaffold for repairing bilateral osteochondral lesions in a rabbit model (Fig. 7A). The piezoelectric scaffold combined with exercise demonstrated a markedly higher ICRS macroscopic evaluation score compared to both the sham operation and control groups. These results suggest that combining a biodegradable piezoelectric scaffold with exercise-induced joint loading effectively promotes stem cell differentiation and cartilage regeneration in a rabbit knee OA model [281]. Duan *et al.* developed a dual-layer PLGA porous scaffold for implantation in a rabbit model with knee osteochondral defects to evaluate its therapeutic efficacy. The findings showed that the cell-seeded dual-layer scaffold promoted superior surface remodeling compared to the acellular scaffold. This demonstrates that the dual-layer PLGA porous scaffold supports long-term osteochondral restoration through tissue engineering and *in vivo* regeneration [282].

Hyaluronic acid, with its distinctive molecular structure and physicochemical properties, plays several critical physiological roles. These roles include joint lubrication, regulation of vascular permeability, protein, water, and electrolyte transport, and wound healing [283]. In joint repair, hyaluronic acid has gained significant attention from researchers due to its remarkable therapeutic effects. For example, Xu *et al*. designed a continuous single-phase gradient scaffold with gradient distribution of chitosan (CS), sodium hyaluronate (SH) and graphene oxide (GO) modified nano-hydroxyapatite (nHAP) in the scaffold [284] (Fig. 7C). After 12 weeks of implantation, significant calcium accumulation and blood vessel formation were observed, effectively simulating the cartilage-subchondral bone interface and promoting BMSC proliferation for joint repair [284]. Wang *et al.* engineered a double-layered scaffold composed of collagen (COL), CS, and sodium hyaluronate. The transition layer, featuring a microtubule array, was crafted from a blend of COL, CS, and silk fibroin (SF). This innovative double-layer bionic cartilage scaffold was then implanted into a rabbit model of KOA to evaluate its reparative capabilities on cartilage defects [285], [286]. He *et al*. designed a biomimetic low-temperature gel scaffold based on hyaluronic acid (Fig. 7B). Compared to hydrogels, HAGM cryogels offer a superior microenvironment for chondrocytes, higher cell proliferation rates, and potential for non-surgical cartilage repair [287]. Wang *et al.* developed a biomimetic scaffold using an in-situ self-assembled gel composed of glyceryl monooleate (GMO) and hyaluronic acid (Fig. 7D) [288], [289].

Collagen and extracellular matrix (ECM) have attracted considerable attention in OA therapy. This is likely due to collagen type II being a major component of articular cartilage. The diversity of ECM components also increases its functional diversity. Chitosan, a representative product of marine collagen, has been widely used in tissue engineering and regenerative medicine, with many products already available on the market. Li *et al*. used extracellular vesicles-chitosan oligosaccharides conjugate (EVs-COS conjugate) to repair cartilage injuries in a rat model [290]. Peng *et al*. developed a novel porous hydrogel composed of polyvinyl alcohol (PVA) and chitosan, which was implanted into a rabbit model with articular cartilage defects to evaluate its repair potential [291]. The findings indicate that the composite scaffold facilitates cartilage repair and enhances OA outcomes. The composite scaffold is expected to be used as a cartilage repair tissue engineering material for tissue repair. Huang *et al.* used freeze-dried type II collagen sponge to cross-link with acellular normal porcine subchondral bone to prepare calcified cartilage zone (CCZ) scaffolds [292]. Bolaños *et al.* developed acellular cartilage-derived matrix (CDM) scaffolds, both standalone and composite versions with a calcium phosphate (CaP) base. These scaffolds were then implanted into the femoral cartilage defects of horses to study their reparative capabilities [293]. Chen *et al*. designed collagen scaffolds with radial orientation and random orientation of stromal cells derived factor-1 (SDF-1) and implanted them into rabbit osteochondral defect models for repair verification (Fig. 7E). In the radial and SDF-1 radial orientation groups, the defects were firm and smooth. In contrast, the randomly oriented group formed only soft and fragile tissues [294]. Das *et al*. developed goat auricular cartilage ECM-derived scaffolds and implanted them into a thoracolumbar defect model in New Zealand rabbits to evaluate repair efficacy. No inflammation was observed around the acellular cartilage scaffold, which exhibited excellent adaptability, promoting stem cell proliferation, chondrogenic differentiation in vitro, and osteochondral regeneration [295].

Cartilage repair and OA treatment inevitably drive an increased demand for collagen. The use of collagen and matrix proteins for cartilage repair and OA treatment has become a major research focus. Effective scaffolds can reduce treatment time and minimize side effects in OA therapy. Researchers are increasingly using synthetic biology, physical, and chemical methods to functionalize scaffolds, including fusion proteins with functional factors, ECM hydrogels, exosome-loaded macromolecular compounds, and adhesive polymer materials. Recent advances in biomaterials have enabled better control and guidance of seed cell-mediated tissue regeneration. Advances in material preparation technology now allow for better simulation of the physiological cell microenvironment, enabling precise regulation of cell behavior and functional phenotypes at molecular and cellular levels, and enhancing engineered tissue regeneration [296] (Fig. 7F).

### Scaffolds loaded with OA-related factors

**Fig. 8:**
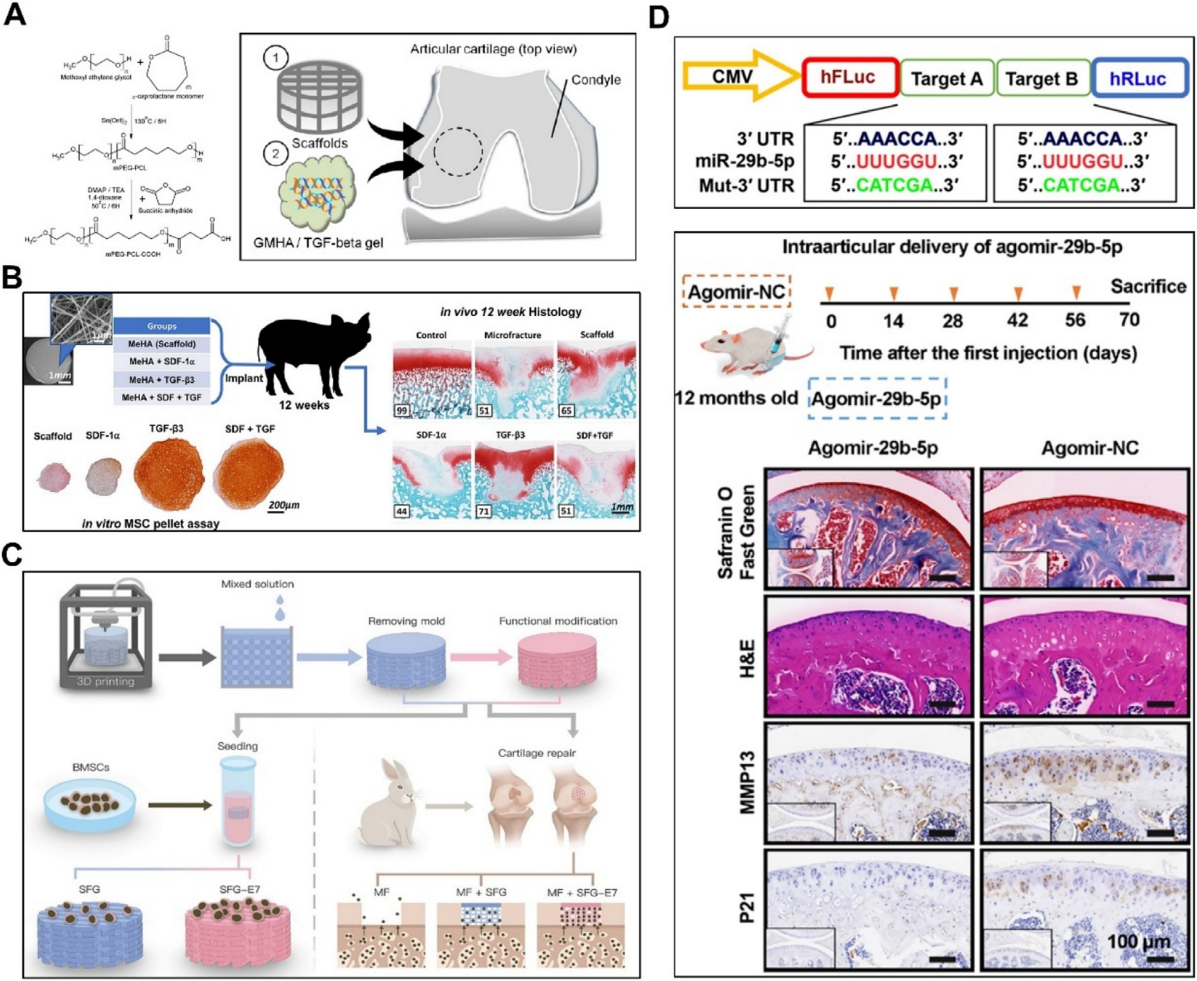
Stent load stimulating factor strategies of tissue engineering in cartilage repair and OA treatment.

The addition of stimulators has an important effect on cell differentiation and function, accurately regulates cell fate, and promotes tissue and organ regeneration (Fig. 8). Previous studies have demonstrated that scaffolds can be loaded with OA-related factors. These factors include tissue inhibitor of metalloproteinase 3 (TIMP3) [267], Fibroblast growth factor-18 (FGF-18) [297], Growth factor Nell-1 [298], SDF-1 [299], Transforming growth factor-β1 (TGF-β1) [300] (Fig. 8A), SDF-1α/ TGF-β3 [301] (Fig. 8B), BMSC specific affinity peptide (E7) [302] (Fig. 8C). These factors effectively recruit chondrocytes and stem cells. They also create a conducive microenvironment for cell growth and differentiation, enhance cartilage tissue formation, and effectively treat OA.

In recent years, immune regulation has also become a new strategy for the treatment of OA, which has attracted the attention of researchers. Glass *et al*. designed and engineered cartilage with immunomodulatory properties, and induced IL-1 expression for cartilage repair through scaffold-mediated lentivirus gene delivery. The findings indicated that the scaffold generated therapeutic Interleukin-1 Receptor Antagonist (IL-1Ra) levels in response to IL-1 during *in vitro* inflammation challenges [303], and had a tendency to reduce MMP activity, which proved that the use of gene therapy to design functional implants for cartilage repair has great potential [303]. Zhu *et al*. introduced a miRNA delivery system based on hydrogels, depicted in Fig. 8D, aimed at diminishing chondrocyte aging. This approach was designed to foster a regenerative microenvironment conducive to the revitalization of impaired cartilage. The study's findings demonstrated that this strategy could significantly ameliorate the condition of articular cartilage, postpone senescence, and curb the accelerated progression of OA [304]. This injectable approach holds significant potential for clinical translation. Growth factors are loaded in the scaffold and form a dynamic microenvironment with a concentration gradient and a local reaction microenvironment, which can change the fate and function of cells. However, the side effects, high cost and immunogenicity of high-dose growth factors still plague researchers.

### Cells, biomaterials, and growth factors combination in tissue engineering for OA treatment

Tissue engineering is fundamentally based on the triad of seed cells, biomaterials, and growth factors [305]. Biomaterials emulate the natural ECM, offering an optimal environment for cell growth and adhesion while preserving cell viability and functionality. The surface's physical and chemical attributes, along with signaling molecules, profoundly influence cellular activities like adhesion, proliferation, migration, and differentiation. These interactions are crucial as they directly impact cellular function and the overall process of tissue regeneration. Zhang *et al*. developed a double-layer bionic scaffold. The upper layer of the construct was created by incorporating Kartogenin and extracted MSCs into the mPEG-block-poly (L-valine) thermal gel, while the lower layer consisted of a bone morphogenetic protein-2 embedded poly (lactide-co-glycolide)/hydroxyapatite porous scaffold [306]. The findings indicated that the fabricated bionic biphasic scaffold demonstrated effective therapeutic outcomes for the comprehensive repair of cartilage and subchondral bone, highlighting its significant application potential [306]. Yejun Hu and colleagues investigated the therapeutic impact of a dual approach: collagen-filament (CSF) scaffolds releasing SDF-1 and intra-articular injections of ligament-derived stem cells progenitor cells (LSPCs) [307]. The rabbit model with knee joint osteochondral defects was established. The study's findings indicated that the simultaneous administration of LSPCs injections and SDF-1 releasing silk scaffolds constituted an effective treatment strategy for OA [307], [308].

There are more and more studies on the combination of seed cells, biomaterials and growth factors in the treatment of OA. However, scientists have started from the selection of materials, scaffold structure and surface conformation, cell selection, and the function of stimulating factors to achieve the greatest OA treatment effect in the simplest combination. This move is to reduce side effects and save costs, and better benefit OA patients.

## Future research avenues and limitations

Oral medications [309], including the cartilage matrix components chondroitin sulfate and glucosamine have short-term effects to delay the onset of OA. Of these, whether glucosamine is an effective treatment for OA remains controversial [310]. The anthraquinone derivative diacerein is a new generation of OA therapeutic drug, exhibits anti-inflammatory and pain-relieving properties and also enhances cartilage matrix synthesis, aiding in cartilage repair. However, its poor water solubility and bioavailability, as well as gastrointestinal adverse reactions associated with long-term use, limit its clinical application [83]. Normally, MMPs and the matrix metalloproteinase inhibitor TIMP maintain a relative balance *in vivo* and have a controlling effect on MMP-mediated cartilage ECM degradation. Under abnormal conditions, in response to MMPs-mediated cartilage degradation, tetracycline, doxycycline and pentosan polysulfate sodium can inhibit the hydrolysis of ECM by MMPs, thereby slowing down the progression of OA [82].

Sodium hyaluronate [11], chitosan, PRP, platelet lysate are commonly used for intra-articular injections [311], which lubricate the joint cavity, relieve pain, and reduce cartilage damage and joint adhesions, but the long-term effects are unsatisfactory, and they are almost ineffective in patients with severe OA [312]. In addition, there are no standardized criteria for the formulation and appropriate concentration of these injectable drugs.

In recent years, biomaterials, especially polymers, polysaccharides, proteins and peptide hydrogels, have received increasing attention for the repair of OA cartilage damage [313]. For example, Ge *et al*. [314] added TGF-β1 affinity peptide to chitosan, Shi *et al.* [315] prepared injectable hydrogel scaffolds containing Kartogenin nanoparticles, Ouyang *et al.* [316] engineered a biomimetic joint 'coating' with a chondroitin sulfate bridge layer and a gelatin methacrylate top layer enriched with hyaluronic acid, facilitating swift and secure curing when adhered to cartilage lesions. Recent research indicates that collagen-based hydrogels stimulate ECM formation and enhance cartilage repair, while collagen type II helps prevent chondrocyte dedifferentiation [317]. Zhang *et al.* [318] reported that collagen type I hydrogel sustained release SDF-1 to treat cartilage injury and found that it could significantly promote defect repair. Meidrix biomedicals GmbH has developed a mouse tail-derived cartilage regeneration collagen (ChondroFiller®, German), which can efficiently repair cartilage damage by arthroscopic injection. Medical tissue engineering has changed the traditional way of treating OA, but whether it can completely block the occurrence of OA or cure OA is still to be expected before reaching the clinical stage. In addition, these methods are mostly used for joint protection or for early-stage treatment of OA and have not been shown to repair serious cartilage defects. For major cartilage defects, cell transplantation is required for cartilage regeneration.

ACT [319] and MSC transplantation [320] have been clinically used to repair articular cartilage defects. ACT effectively alleviates pain and enhances joint mobility; however, it may also lead to complications like graft hypertrophy and fibrosis of the articular cartilage [321], [322]. It is worth noting that MSCs exhibit both osteogenic and chondrogenic properties, and they can actively migrate to damaged joint areas under the influence of the body's microenvironment to repair and regenerate cartilage tissue [323]. Furthermore, MSCs can secrete various cytokines that can suppress inflammatory cytokines [324], reduce the destruction of bone and synovial joints, and effectively control the progression of OA [325]. MSC products for the treatment of OA have been approved for marketing, such as CARTISTEM® approved in South Korea for the treatment of degenerative arthritis and cartilage defects, and StemOne® approved by the Drug Controller General of India for allogeneic cell therapy for knee OA. However, in clinical applications, caution is needed, and further research and clinical trials may be required to overcome these challenges and risks, including immunogenicity, high costs, fever and allergic adverse reactions, technical and regulatory issues and the clinical mechanisms of action are still limited [326].

In addition to the above research methods for the treatment of OA, molecular mechanism analysis of the deterioration process of OA has led to the conclusion that inhibition of vascularization may slow or block the progression of OA. It has been found that erosive lesions of OA begin at the junction of synovium and cartilage. A range of inflammatory agents and cytokines contribute to the degradation of articular cartilage following the invasion of synovial blood vessels into it. The destruction of cartilage and underlying bone, marked by synovial hyperplasia and hypertrophy, is angiogenesis-dependent within the synovium [327]. In the early stage of OA, synovial inflammation is evident and is mainly characterized by macrophage infiltration. Synovial macrophages can secrete VEGF, which has been shown to induce OA in mice by intra-articular injection [328]. Macrophage-derived IL-1β triggers NO and PGE2 expression, facilitating synovial blood vessel expansion and proliferation [329]. TNF-α activates the expression of VEGF, basic fibroblast growth factor (bFGF), and platelet-derived growth factor (PDGF) in tissues, thereby stimulating vascular endothelial cell (EC) proliferation and the growth of OA synovium and subchondral bone [330]. Chen *et al.* [331] found that the level of SDF-1 in knee specimens from OA patients was significantly higher than that of normal subjects. High levels of SDF-1 can not only stimulate the release of MMPs, but also accelerate synovial angiogenesis and promote the release of inflammatory factors by inducing VEGF expression.

**Fig. 9:**
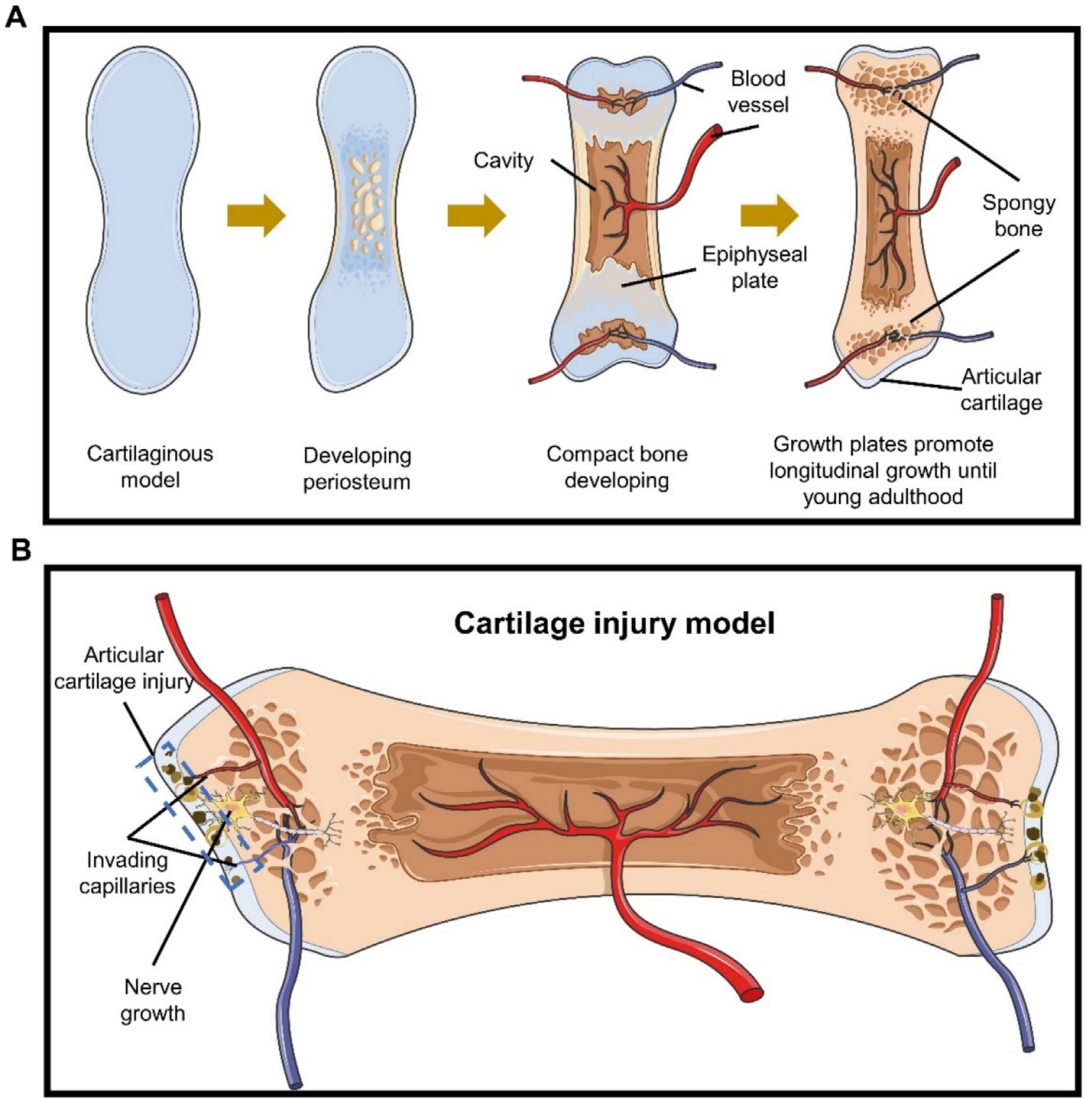
**Process of bone development and articular cartilage injury.** A. Process of bone development; B. Blood vessels invade the cartilage area, exacerbating the injury and resulting nerve growth that causes severe pain in the patient.

In the pathogenesis of OA, inflammatory stimulation and angiogenesis are closely interrelated and mutually reinforcing, resulting in a vicious circle. On the one hand, inflammatory stimulation induces angiogenesis; on the other hand, the infiltration of new blood vessels to macromolecular substances, aggravating local edema, while also transporting inflammatory cells and supplying oxygen, thereby sustaining and perpetuating inflammation. In summary, vascular hyperplasia of joints leads to increased synovial hypertrophy, cartilage destruction, ossification and osteophyte formation (Fig. 9A). In addition, vascular hyperplasia is often accompanied by nerve ingrowth, which will further aggravate pain symptoms [332] (Fig. 9B). There is a direct correlation between the severity of OA and the extent of vascular invasion, indicating that anti-angiogenic therapies may offer a novel strategy for managing this condition.

## Concluding remarks

This review provides a comprehensive overview of the epigenetic mechanisms, pathogenic processes, and therapeutic approaches in OA. It addresses the side effects associated with oral and injectable medications, evaluates the current landscape of surgical interventions, explores rehabilitative physical therapy approaches, and discusses the limitations of emerging cell-free biomaterials and cell-based therapies. By analyzing key signaling pathways, the review identifies potential drug targets, offering new perspectives for the development of more effective OA therapies.

## CRediT authorship contribution statement

**Xueliang Peng**: Collected the primary literature and critically reviewed this manuscript. **Xuanning Chen**: Collected the primary literature and critically reviewed this manuscript. **Yifan Zhang:** Reviewed a part of the literature and wrote a part of this manuscript. **Zhichao Tian:** Participated in the discussion. **Meihua Wang:** Participated in the discussion. **Zhuoyue Chen:** Designed the concept, reviewed the literature and wrote this manuscript. All authors read and approved the final manuscript.

## Ethical approval and consent to participate

There was no involvement of humans or animals in this study.

## Compliance with ethics requirements

This article does not contain any studies with human or animal subjects.

## Funding

This work was supported by the National Natural Science Foundation of P. R. China (No. 32171329 to Z.Y.C). Special Support Plan for High-level Talents (No. 334042000022), and Innovation Team Program in Shaanxi Province (No. 2019TD-032). Major special project of Qin Chuang Yuan (23LLRHZDZX0010).

## Declaration of competing interest

The authors declare that they have no known competing financial interests or personal relationships that could have appeared to influence the work reported in this paper.

## Data Availability

Data will be made available on request.
