# Supplementary material for: Advances in the pathology and treatment of osteoarthritis
Source: J Adv Res. 2025 Jan 30;78:257–83. doi: 10.1016/j.jare.2025.01.053 (PMC12684957; doi:10.1016/j.jare.2025.01.053)
Supplement: Supplementary Data 1 [file mmc1.docx]

**Advances in the pathology and treatment of osteoarthritis**

**S Table 1: Expression of related genes in osteoarthritis.**

| **OA classification** | **Cells** | **Genes** | **Gene expression pattern** | **Time** | **Refs.** |
| --- | --- | --- | --- | --- | --- |
|  |  | DVWA | DVWA gene specifically expressed in cartilage, encodes for a protein showing double von Willebrand factor A domains (VWA domain). | 2015 | **[1]** |
|  |  | ID3, HES1, JUN | Highly expressed genes (ID3, HES1, JUN and others) which are mainly involved in protein binding and RNA metabolic process. | 2018 | **[2]** |
|  |  | KLHL21, SGMS2, ITGA5ID3 | Highly expressed genes (KLHL21, SGMS2, ITGA5ID3 and others) which are associated with angiogenesis and cell motility. |  |  |
| Early stages | Ecs (effector chondrocytes) | C2orf82, CLEC3A, CYTL1 | The signature which includes related to alcohol biosynthesis, skeletal system development and cholesterol biosynthesis. |  |  |
| Stages 0 and 1 | Chondrocytes | FRZB, C2orf82, TF | Highly expressed genes (FRZB, C2orf82, TF and others) which are mainly involved in skeletal system development and cellular responses to stress, suggesting the early changes that occur during OA pathogenesis. |  |  |
| Stage 3 and 4 | Chondrocytes | TNC, TGFBI, CRTAC1 | Highly expressed genes (TNC, TGFBI, CRTAC1 and others) which are mainly involved in extracellular matrix organization and collagen catabolism. |  |  |
| Early stages | RegCs (regulatory chondrocytes) | CHI3L, CRTAC1, CYTL1, CHI3L1 | Related to responses to endogenous stimuli and cellular responses to oxygen-containing compounds. |  |  |
| Early stages | RegCs (regulatory chondrocytes) | CD74, CD80, CD86, HCADPA1 | Possess gene expression related to antigen-processing and antigen-presenting and detected the expression of the major histocompatibility complex class of genes. The genes were expressed at higher levels in a small proportion of RegCs. |  |  |
| Each stage | HomCS(homeostatic chondrocytes） | PER1, SIRT2 | High expression, regulation of cell homeostasis: regulation of cell cycle, development and RNA metabolism and biosynthesis. |  |  |
| Early stages | Aged hMSCs | CBX4 | CBX4 protein is downregulated in aged hMSCs. | 2019 | **[3]** |
| Early stages | Chondrocyte | EZH2 | Elevated EZH2 expression was disclosed in the pathological groups by both immunofluorescence staining and Western blot. Interestingly, the 6-week post-surgery group showed higher expression of EZH2 than the groups at the other time points, which suggested that EZH2 might play a more important role in the early stages of the OA process. | 2020 | **[4]** |
|  |  | S100A4, LGALS1 | Transcriptional PTTG1 and its downstream target gene S100A4, LGALS1 were both upregulated in osteoarthritic cartilage. S100A4 and LGALS1 both could promote inflammation and elicit catabolic signaling pathway. | 2021 | **[5]** |
|  |  | NRP1, CYTOR | CYTOR functioned as a competing endogenous RNA (ceRNA) to positively regulate NRP1 expression by sponging with miRNA-206, which could positively affect epithelial to mesenchymal transition, cancer progression, and cell growth. NRP1 can induce vasculogenesis and angiogenesis, which contribute to structural damage of cartilage and pain in OA. |  |  |
|  | Bone articular cartilage superficial cells | FOXO1, FOXO3 | With the joint aging, the expression of FOXO1 and FOXO3 was reduced in the superficial zone of cartilage and the FOXO protein was deactivated in abnormal chondrocytes of OA cartilage. It has been reported that the overexpression of FOXO1 protected the chondrocytes from OA damage. |  |  |
| Early stages | Cartilage progenitor cell | GDF5rs143384 | Gdf5-expressing progenitors are continuously recruited into joint interzones throughout development and their progeny retain skeletal joint stem/progenitor activity in adulthood. Following injury to the joint surface, Gdf5-lineage mesenchymal stromal/stem cells (MSCs) proliferate to underpin synovial hyperplasia and migrate to the site of injury, through the activity of the transcriptional co-factor Yes-associated protein (Yap), where they repair cartilage. | 2020 | **[6]** |
|  |  | ASPN | Asporin suppresses TGF-beta-mediated expression of the genes aggrecan (AGC1) and type II collagen (COL2A1) and reduced proteoglycan accumulation in an in vitro model of chondrogenesis. | 2005 | **[7]** |
|  |  | SMO (rs143083812) | The SMO gene is central to the Hedgehog (HH) signaling pathway, which is essential for embryonic development and in the regulation of stem-cell homeostasis, including the regulation of osteogenesis and chondrocyte differentiation. In human osteoarthritic cartilage, expression of members of the HH pathway has been shown to be upregulated, and mice harboring a Smo mutation conferring constitutive activity that is specifically expressed in chondrocytes develop severe osteoarthritis. | 2018 | **[8]** |
|  |  | IL11(rs4252548) | IL-11 is a member of the gp130 family of cytokines that stimulates osteoclast differentiation and bone turnover, among other functions. |  |  |
|  |  | COL11A1 | The *COL11A*1 gene encodes a fibril-forming collagen of the extracellular matrix; it is expressed mainly in cartilage, where it forms a heterotrimer with the major cartilage collagen, COL2A1. The gene is also expressed in intervertebral discs, the ocular vitreous and the inner ear. |  |  |
|  |  | SOX5 | SOX5 expression is upregulated and is associated with back pain and lumbar disc degeneration. SOX5 inactivation leads to defects in bone development, such as chondrodevelopment, notochords, and intervertebral discs in mice. | 2021 | **[9]** |
|  |  | COL2A1 | Collagen type II alpha 1 chain (COL2A1) codes for an essential structural component of cartilage and is important for joint formation and bone growth. |  |  |
| Early stages |  | FBN2 | Fibrillin 2 (FBN2) encodes a glycoprotein that forms microfibrils in the extracellular matrix and has a major role during early morphogenesis. Fibrillin’s potently regulate pathways of the immune response, inflammation, and tissue homeostasis, are important in bone remodeling, and regulate local availability of BMP and TGF-β. |  |  |
|  |  | PTCH1 | Patched 1 (*PTCH1*) codes for a receptor for Hh ligands and regulates the activity of smoothened, frizzled class receptor (*SMO*, another effector gene associated with a known lead SNV). When bound, PTCH1 relinquishes its inhibitory effect on SMO and activates the Hh signaling cascade, which plays an important role in controlling the proliferation of chondrocytes and also in stimulating osteogenesis during endochondral bone formation and longitudinal growth. |  |  |
|  |  | TRIOBP | TRIOBP-1 is ubiquitously expressed and interacts with TRIO and f-actin binding protein that together play crucial roles in neuronal morphogenesis. |  |  |
|  |  | MTMR2 | Myotubularin related protein 2 (*MTMR2*) has an important role in membrane targeting, vesicular trafficking, and regulation of signal transduction pathways. |  |  |
|  |  | TLR4 | Receptor 4 (TLR4) plays a fundamental role in pathogen recognition and activation of the innate immune response. TLR4 is also activated by host-derived molecules generated by damaged tissues related to different musculoskeletal pathologies. |  |  |
|  |  | NR3C1 | Nuclear receptor subfamily 3 group C member 1 (NR3C1) encodes the glucocorticoid receptor (GR) which circulates in the cytoplasm and is involved in the inflammatory response. In osteoarthritis, endogenous glucocorticoid signaling in osteoblasts and chondrocytes is detrimental. |  |  |
|  |  | PFKM | It encodes a muscle isozyme that catalyzes the phosphorylation of fructose-6-phosphate during glycolysis. |  |  |
|  |  | CTSK | Cathepsin K (encoded by the CTSK gene) is an enzyme that plays a critical role in collagen degradation within osteoclasts. |  |  |
|  |  | NOG | Noggin (NOG) as an osteoarthritis effector gene. Mutations in NOG cause a whole range of bone and cartilage phenotype. |  |  |
|  |  | NOS3 | Nitric oxide synthase 3 (NOS3) encodes the vascular endothelium isoform of nitric oxide synthase (eNOS). NOS3 is associated with sporadic limb defects in mice. |  |  |
|  |  | WNT9A | WNT9A expression was significantly increased in OA lesioned cartilage compared with preserved OA cartilage. | 2020 | **[10]** |
|  |  | IL6, IL8 | Previous studies demonstrated that IL6 and IL8 expression levels were increased in OA synovial tissues ,IL6 and IL8 primarily regulate immune responses and inflammatory responses. | 2018 | **[11]** |
|  |  | MMP3, MMP9 | Which demonstrated high expression of MMP9 and MMP3 in OA synovial tissues,The role of MMP9 is primarily to degrade and remodel the extracellular matrix, and the role of MMP3 is to degrade extracellular matrix proteins. |  |  |
|  | Macrophage-CSF and macrophage-CSF and granulocyte-CSF | CSF1R | CSF1R encodes a receptor protein for colony stimulating factor 1, which mediates the majority of the biological effects of this cytokine. |  |  |
|  | Cartilage of OA | IGF1 | IGF1 encodes an active protein peptide substance that promotes cell growth and is essential for sugar, lipid, protein metabolism and inorganic salt metabolism,the IGF1 content in OA synovial fluid was twice that in normal fluid. |  |  |
|  | OA synovial tissues | TYROBP | TYROBP is involved in immune and inflammatory reactions. TYROBP was more highly expressed in OA synovial tissues compared with healthy synovial tissues. |  |  |
|  | Chondrocyte | MGP | The hand OA risk allele is associated with lower expression of MGP in articular cartilage and suggested the variant causes increased OA burden by decreasing levels of MGP which could lead to increased cartilage calcification. | 2020 | **[12]** |
|  | OA cartilage | CRY2 | CRY2 levels are significantly depressed in OA cartilage. |  |  |
| Terminal stage |  | POU2AF1, IGL@, IGJ, PAX5 | A cluster of genes is related with immune response, such as POU2AF1, IGL@, IGJ, PAX5, TNFRSF17, IGHM, MIF, IGKV1-5, TNFSF11 (RANKL), ILF2 (NFAT), LAX1, IGHD, MS4A1 (CD20), IGKV4-1, FAIM3, IGKV3-20, CD79A, CD19 and IGKC. And we found that these genes related with immune response were down-regulated in end-stage of OA samples. | 2014 | **[13]** |
|  |  | TNFRSF17, IGHM, MIF, IGKV1-5 |  |  |  |
|  |  | TNFSF11（RANKL）,ILF2（NFAT）,LAX1 |  |  |  |
|  |  | IGHD, MS4A1(CD20),  IGKV4-1 |  |  |  |
|  |  | FAIM3, IGKV-3-20, CD79A |  |  |  |
|  |  | CD19, IGKC |  |  |  |
| End stage |  | HOXA11, RUNX2 | Some genes were found to be related with cartilage development, such as HOXA11, RUNX2 (runt-related transcription factor 2) which were down-regulated in end stage of OA. |  |  |
| End stage |  | B3GNT9, MAN2A1, ALG8, SERP1 | A cluster of genes related with protein glycosylation, such as B3GNT9, MAN2A1, ALG8, SERP1 was down-regulated in end-stage OA which indicated that the decrease of these genes’ expression may contribute to the degradation of cartilage. |  |  |
| Terminal stage | Articular chondrocyte | ADAMTS1, ADAMTS4, ADAMTS5 | ADAMTS8 levels increased and ADAMTS1,4,5 levels decreased in the end stage of hip osteoarthritis. | 2019 | **[14]** |
| Terminal stage | Articular chondrocyte | ADAMTS8 | ADAMTS8 levels increased and ADAMTS1,4,5 levels decreased in the end stage of hip osteoarthritis. |  |  |
| Early and middle stage |  | ADAMTS9, ADAMTS15 | There was no significant difference between the femoral neck fractures group and the hip degenerative arthritis group in terms of ADAMTS9 and ADAMTS15 mRNA expression. |  |  |
| More advanced |  | DPEP1 | Expression of the DPEP1 gene was found to be lower in OA samples than in non-OA samples in rodents by combining transcriptomics studies of murine OA models | 2021 | **[15]** |
| Grade 2 | Extracellular matrix in the middle region, deep region and shallow region | RANKL | An increase in RANKL mRNA expression in grade 2 OA cartilage and increased RANKL protein staining,it may promote osteoclast genesis and increase subchondral bone turnover. | 2012 | **[16]** |
| Grade 3 |  | OPG | Up-regulated expression. |  |  |
|  |  | SOX9, ACAN, COL2A1, DKK1, FRZB | A gradual decrease in mRNA expression of SOX9, ACAN and COL2A1. | 2016 | **[17]** |
|  |  | RUNX2, COL10A1, COL1A2, IHH, AXIN2 | Hypertrophic markers (RUNX2, COL10A1 and IHH) increased during OA progression. |  |  |
| Late stage | Bone marrow mesenchymal stem cells | Col4a1, Col4a2, Col8a1, Col10a1, Col11a1 | Several collagens (i.e., Col4a1, Col4a2, Col8a1, Col10a1, and Col11a1) were clearly down-regulated in bone marrow mesenchymal stem cells from patients with advanced OA | 2012 | **[18]** |
| Early stages | Fibroblastic-like chondrocyte | COL6A1, COL6A3 | The expression of both COLA6A1 and COLA6A3 was observed up-regulated in both chondrocytes and fibroblasts, COL6, one of Collagen Type VI family, which was an essential component collagens. , COLA6A1 and COLA6A3 participated in the pathogenesis of OA through the focal adhesions and ECM-receptor interaction pathway. | 2020 | **[19]** |
|  | Chondrocytes | RHOA | High expression. There was no difference in expression of both COL6A1 and RHOA. |  |  |
|  | Fibroblastic-like chondrocyte | ACTG1 | Actin gamma 1, encoded by ACTG1, is a cytoplasmic actin found in all cell types and is involved in various types of cell motility and the maintenance of the cytoskeleton. Up-regulated ACTG1 might participate in the pathological process of OA through the focal adhesion pathway in chondrocytes and fibroblasts. |  |  |
| Early stages |  | CCL3, CXCL8, IL1B | CCL3, CXCL8, and IL1B were mainly upregulated at the start of the pseudo time, COL1A1, COL1A2, and PRG4 were upregulated near the end, and FGF1, KRT17, and NGF were upregulated during the middle. | 2021 | **[5]** |
| Late stage |  | COL1A1, COL1A2, PRG4 |  |  |  |
| Middle stage |  | FGF1, KRT17, NGF |  |  |  |
|  |  | S100A4, LGALS1 | Target gene S100A4, LGALS1 were both upregulated in osteoarthritic cartilage. There is a chance that the activated expression of S100A4 and LGALS1 is caused by the activated expression of PTTG1. S100A4 and LGALS1 both could promote inflammation and elicit catabolic signaling pathways. |  |  |
|  |  | NRP1, CYTOR | NRP1 co-expressed with CYTOR as upregulated genes in osteoarthritic cartilage. |  |  |
| Late stage |  | EGFR | In the late stage of OA, human OA samples contain significantly more activated EGFR than controls. | 2019 | **[20]** |
|  |  | CDC42 | CDC42, a member of Rho family small GTPases, is highly expressed in both articular cartilage and subchondral bone in a mouse OA model; knockdown of Cdc42 expression or inhibition of Cdc42 activity robustly attenuates knee joint destruction in mouse. |  |  |
|  | Fibroblast-like synoviocytes (FLS) and macrophages | MCF2L | Increased MCF2L expression in OA synovium tissue. | 2015 | **[21]** |
| Late stage | Fibroblasts | COL1A1, COL5A1, TIMP1, MMP13, SDC1 | The qRT-PCR results confirmed that the expression levels of COL1A1, COL5A1, TIMP1, MMP13, and SDC1 were obviously increased in OA samples. | 2020 | **[22]** |

**S Table 2: Treatment of osteoarthritis related drugs and their mechanism of action.**

| **Drug name** | **Effects** | **Action mechanism** | **Time** | **Refs.** |
| --- | --- | --- | --- | --- |
| Matrix metalloproteinase (MMP) inhibitor, PG-116800 | Musculoskeletal adverse. | Inhibit matrix degrading enzymes and inhibit cartilage damage | 2007 | [23] |
| Pharmaceutical-grade chondroitin sulfate (CS) | Proved to be safe and effective in hip, knee and hand OA, acting on signs, symptoms and structural changes. The use of hpCS reduced the use of NSAIDs and their side effects. | hpCS antagonizes interleukin-1(IL-1)-induced increases in p38 mitogen-activated protein kinase (p38MAPK) and signal-regulated kinase 1/2 (Erk1/2) phosphorylation and decreases in nuclear factor-B (NFB) nuclear translocation and as a consequence reduced formation of pro-inflammatory cytokines, IL-1 and TNF, and pro-inflammatory enzymes, such as phospholipase A2 (PLA2), cyclooxygenase 2 (COX-2) and nitric oxide synthase-2 (NOS-2). | 2020 | [24] |
| Hyaluronic acid (HA) | In synovial joints, it provides viscoelasticity to synovial fluid. Helps to trap water within the joints, providing resistance to the cartilage against pressure by increasing the amount of water in the cartilage matrix. HA provides better proteoglycan synthesis, joint lubrication, and viscoelasticity. | The amount and weight of HA in synovial fluid in osteoarthritic knee joints were lower than in non-arthritic joints. One of the mechanisms of HA action is to restore the mean median weight of HA at the knee joint. HA also treats osteoarthritis in several interrelated ways: increasing the viscosity of the joint fluid, reducing oxidative stress, and inhibiting phagocytosis. HA can reduce pain by blocking stretch-activated mechanoreceptors that inhibit joint nociceptors. | 2020 | [25] |
| Chitosan oligosaccharides（COS） | Improved the cartilage damage. | Previous study has shown that carboxymethyl-chitosan could protect cartilage through multiple ways, including nitric oxide production inhibition, mitochondrial function modulation and scavenge reactive oxygen species. As a derivative of chitosan, COS may share these key properties. | 2017 | [26] |
| EP4-selective agonist KAG-308 | KAG-308 inhibited OA development through suppression of chondrocyte hypertrophy, catabolism, or inflammation. | Chondrocyte hypertrophy and expression of tumor necrosis factor alpha (TNF) and matrix metalloproteinase 13 (Mmp13) in the synovium were suppressed in the KAG-308-treated mice. In cultured chondrocytes, hypertrophic differentiation was inhibited by KAG-308 and intranuclear translocation of histone deacetylase 4 (Hdac4) was enhanced. In cultured synoviocytes, lipopolysaccharide (LPS)-induced expression of TNF and Mmp13 was also suppressed by KAG-308. | 2019 | [27] |
| N-acetyl phenylalanine glucosamine derivative（NAPA） | Exert anti-inflammatory and chondroprotective activity.The intra-articular injection of NAPA delayed OA progression and preserved cartilage integrity, reducing fibrillation, cell cluster number and the amount of fibrous tissue, in lateral and medial femoral condyles and in the tibial plateau of knee joints. | The addition of NAPA decreased the chondrocyte expression of most of these genes at 24 hours: ADAMTS5, the pivotal matrix-degrading enzymes in osteoarthritis and IL-6 and MCP-1. | 2019 | [28] |
| Capsaicin | Topical therapy addresses the pain that stems from abnormal nociceptive fiber (pain fiber) activity at the level of the skin. | Capsaicin is a potent agonist of the TRPV1 channel, a transduction channel that is highly expressed in nociceptive fibers (pain fibers) throughout the peripheral nervous system. Capsaicin overcomes both liabilities by disrupting peripheral terminals of nociceptive fibers that express TRPV1, and thereby affects all of the potential means of activating that pain fiber (not just TRPV1 function). | 2021 | [29] |
| Phospholipase A2 inhibitor–loaded micellar nanoparticles | Penetrate deep into the cartilage matrix, prolong retention in the joint space, and mitigate OA progression. | In this work, we show that the amount of secretory phospholipase A2 (sPLA2) enzyme increases in the articular cartilage in human and mouse OA cartilage tissues. We hypothesize that the inhibition of sPLA2 activity may be an effective treatment strategy for OA. To develop an sPLA2-responsive and nanoparticle (NP)–based interventional platform for OA management, we incorporated an sPLA2 inhibitor (sPLA2i) into the phospholipid membrane of micelles. The engineered sPLA2i-loaded micellar NPs (sPLA2i-NPs) were able to penetrate deep into the cartilage matrix, prolong retention in the joint space, and mitigate OA progression. These findings suggest that sPLA2i-NPs can be promising therapeutic agents for OA treatment. | 2021 | [30] |
| Novel synergistic drug candidate, CRx-102 | The novel synergistic drug candidate CRx-102 demonstrated efficacy by statistically reducing pain compared to placebo in HOA and was generally well tolerated. |  | 2008 | [31] |
| The topical diclofenac formulations available | Provided significant pain relief in patients with sports and soft tissue injuries involving the ankle, knee or shoulder. |  | 2008 | [32] |
| Iminocyclic alcohol | Be capable of enhancing GAG accumulation in cultured human articular chondrocytes, cartilage tissue and chondrosarcoma cells. | Hexosaminidase participates in the degradation of extracellular matrix in human cartilage; inhibition of hexosaminidase activity with the aid of chemical inhibitors delays the catabolism of cartilage matrix GAGs. | 2001 | [33] |
| Glucosamine | Glucosamine plays a direct anti-inflammatory role, alleviates the painful symptoms of osteoarthritis, improves joint function, and can stop the progression of osteoarthritis. However, in patients with radiographically diagnosed OA, the use of glucosamine does not appear to relieve symptoms or alter disease progression. | Glucosamine fully protected the chondrocytes from IL-1-induced expression of inflammatory cytokines, chemokines, and growth factors as well as proteins involved in prostaglandin E2 and nitric oxide synthesis. It also blocked the IL-1-induced expression of matrix-specific proteases. | 2006 2014 | [34, 35] |
| Xanthan gum (XG) | IA injection of XG once every 2 weeks for 5 weeks decreased the severity of swelling of the knee joint, reduced the damage of cartilage surfaces, inhibited the cells changes, structural changes and loss of Safranin O staining intensity of femoral condyle and tibial plateau and also inhibited cells hyperplasia and infiltration of mononuclear cells in the synovium. |  | 2012 | [36] |
| Low Molecular Weight Xanthan Gum（LM-XG） | An injection preparation with LW-XG (once every 2 weeks for 5 weeks) has been shown to outperform SH (once weekly for 5 weeks) for treating the cartilage damage, owing to its advantages of being biomimetic and being long-acting. | To protect cartilage from damage, reduce the concentration of NO in synovial fluid and reverse the enlargement of knee width. At the cellular level, these effects may be due in part to the fact that LW-XG promotes proliferation while reducing apoptosis of chondrocytes. At the molecular level, these effects are triggered by down-regulation of protein levels of caspase -3 and bax and up-regulation of bcl-2 protein levels in vivo and in vitro cartilage. | 2017 | [37] |
| Phosphocitrate (PC) | Phosphocitrate (PC) inhibits osteoarthritis (OA) in Hartley guinea pigs,PC exert its OA disease modifying effect | PC downregulated the expression of numerous genes classified in proliferation and apoptosis while upregulating the expression of many genes classified in transforming growth factor-β (TGF-β) receptor signaling pathway and ossification. PC also downregulated the expressions of many genes classified in inflammatory response and Wnt receptor signaling pathways. Consistent with its effect on the expression of genes classified in proliferation, ossification, and skeletal development, PC inhibited the proliferation of OA chondrocytes and chondrocyte-mediated calcification while stimulating the production of extracellular matrices. | 2017 | [38] |
| Strontium ranelate (SrRan) | Suggest a positive effect of the use of SrRan in patients with OA, through changes in functional capacity and reduction of progression of morphological parameters and joint degradation. | The inhibition of osteoclastic activity by SrRan has been demonstrated to be related to the reduction in matrix metalloproteinase (MMP) synthesis and modulation of the osteoprotegerin-RANKL pathway. | 2018 | [39] |
| Thermosensitive hybrid hyaluronan/p (HPMAm-lac)-PEG hydrogels | The hydrogel matrix could act in synergy with the drug, in reversing phenomena of inflammation, cartilage disruption, and bone demineralization associated with OA.the developed hybrid hydrogels, not only inhibited the release of inflammatory molecules but were also able to induce MSCs maturation into chondroblasts and new cartilage formation. | Hyaluronic acid was controllably released during network degradation with a zero-order release kinetics, and the release rate depended on cross-link density and degradation kinetics of the hydrogels. When locally administered in vivo in an OA mouse model, the hydrogels demonstrated the ability to restore, to some extent, bone remineralization, proteoglycan production, levels of Sox-9 and Runx-2. | 2019 | [40] |
| Caragana sinica root extracts（CSR） | The severity of OA was improved, and CSR reduced inflammatory mediators, catabolic enzymes, and ECM degradation in MIA induced OA rats. Furthermore, CSR treatment also decreased the OARSI scores in addition to the extent of synovitis. | CSR significantly inhibited the expression of MMPs, ADAMTSs and the degradation of ECM in IL-1β-stimulated chondrocytes. Furthermore, CSR significantly suppressed IL-1β-stimulated of MAPKs, NF-κB signaling pathway. In vivo, CSR and Indomethacin inhibited the production of inflammatory mediators, MMPs and degradation of ECM in MIA-induced model of OA. | 2021 | [41] |
| Solanum xanthocarpum fruit extract（SXF） | Promotes chondrocyte proliferation and improves inflammation. | SXF benefits and ameliorates OA by enhancing the chondrocytes proliferation and preventing the articular cartilage damage through the restoration of their structural molecules, arthritic score reduction, suppression of MMP-3 and COX-2 expression level and up regulation of COL-2 genes expression. | 2021 | [42] |
| Boiogito | Improve the increased knee effusion in OA rats and reduce joint pain. | Boiogito ameliorates the increased knee joint effusion in rats with OA by suppressing pro-inflammatory cytokine IL-1β production in the articular cavity and regulating function of water transport in the synovium. The improvement of hydrarthrosis by boiogito results in the increased HA concentration in synovial fluid, thus reducing joint pain. | 2015 | [43] |
| Indomethacin, GANT-61 | Indomethacin：Indomethacin is a commonly used nonsteroidal anti-inflammatory drug in the treatment of OA. However, over-dose and long-term use of indomethcin may cause more adverse reactions, and damage to liver function. Therefore, a low-dose of indomethacin combined with other drugs that inhibit the development of OA may achieve a better effect on attentuating OA condition. Combined application of indomethacin andGANT-61 significantly promoted tissue integrity in cartilage tissue, and significantly reduced the serum inflammatory factors in OA micecompared. Data suggested that CANT-61 together with low dose of indomethacin can be used in the treatment of OA to slow down the cartilage damage. | Hedgehog signaling pathway is closely related to the physiological and pathological processes of chondrocytes. It plays a key role in chondrogenic differentiation. In osteoarthritis and joint degeneration, activation of the Hedgehog signaling pathway in chondrocytes is involved in joint cartilage degeneration and heterotopic bone formation. Thus, blocking Hedgehog signaling may prevent or delay the progression of OA. GLI, a type of transcription factor with zinc finger structure, is the downstream gene of Hedgehog signaling pathway. With the activation of Hedgehog, GLI is activated and enters into the nucleus to play the function of transcription factor and affect the transcription initiation of downstream genes. For example, GLI promotes the expression of Runx2, leading to hypertrophy of chondrocytes. Moreover, GLI gene knockout can block Hedgehog signaling pathway, which in turn can prevent or delay the development of OA. GANT-61 is a well-known inhibitor of Hedgehog/GLI pathway. Inhibition of Hedgehog signaling significantly suppresses cartilage degradation and osteoarthritis condition. | 2019 | [44] |
| Licofelone | Licofelone is well tolerated and has a GI safety profile similar to placebo and significantly better than naproxen. | Likerone reduces the production of pro-inflammatory leukotrienes and prostaglandins.5-LOX, COX-1 and COX-2 with licofelone—and therefore decreased production of LTs and PGs—has the potential to offer clinically relevant advantages over COX inhibition alone with NSAIDs and selective COX-2 inhibitors, in terms of overall safety. | 2004 | [45] |
| Metformin | Metformin reduced the progression of experimental OA and showed both antinociceptive properties and cartilage protection. The combined administration of metformin and celecoxib-controlled cartilage damage more effectively than metformin alone. | In chondrocytes from OA patients, metformin reduced catabolic factor gene expression and inflammatory cell death factor expression, increased LC3Ⅱb, p62, and LAMP1 expression, and induced an autophagy–lysosome fusion phenotype. | 2021 | [46] |
| Bilobalide | It has anti-inflammatory and antioxidant pharmacological properties. Bilobalide exerted significant anti-inflammatory and anti-extracellular matrix (ECM) degradation effects in a rat model of post-traumatic OA (PTOA) induced by anterior cruciate ligament transection (ACLT). | Bilobalide significantly inhibited the production of inducible nitric oxide synthase (iNOS), cyclooxygenase-2 (COX-2), and matrix metalloproteinase 13 (MMP13) in ATDC5 chondrocytes induced by Interleukin-1β (IL-1β). At the molecular level, bilobalide induced chondrocyte autophagy by activating the AMPK/SIRT1/mTOR signaling pathway, which increased the expression of autophagy-related Atg genes, up-regulated the expression of LC3 protein, and reduced the expression of the p62 protein. | 2022 | [47] |
| Charged chitosan nanoparticles | Exceptional lubrication and continuous drug release ability. | In this paper, one kind of novel biomimetic nanoparticles (NPs) lubricant, named CS-PS, is synthesized through chemically grafting hydrophilic sulfonic acid (SO3-) groups onto the surface of biocompatible and biodegradable chitosan (CS) NPs. Compared with control CS NPs, the as-synthesized CS-PS NPs exhibits excellent hydration and stability because of negatively charged surface zeta potential, along with extraordinary lubrication performance in water for realizing a super-low friction coefficient (COF) as ∼0.01 at the sliding interface of PDMS elastomer-Ti6Al4V disk. | 2021 | [48] |
| Thienoindazole derivative compoundTD-198946 | TD-198946 strongly induced chondrogenic differentiation without promoting hypertrophy in cell and metatarsal organ cultures. When administered directly into the joint space, TD-198946 successfully prevented and repaired degeneration of the articular cartilage. | TD-198946 exerted its effect through the regulation of Runx1 expression, which was downregulated in both mouse and human OA cartilage compared with normal tissue. | 2013 | [49] |
| ERRγ inverse agonist, GSK5182 | Treatment with GSK5182 reduced DMM-induced bone remodeling including cartilage destruction, osteophyte development, and subchondral bone sclerosis. | Estrogen-related receptors (ERRs) are the first identified orphan nuclear receptors. The ERR family consists of ERRα, ERRβ, and ERRγ, regulating diverse isoform-specific functions. ERRγ expression increased following chondrocyte exposure to various pro-inflammatory cytokines, including interleukin (IL)-1β, IL-6, and tumor necrosis factor (TNF)-α. Pro-inflammatory cytokines dose-dependently increased ERRγ protein levels. In mouse articular chondrocytes, adenovirus-mediated ERRγ overexpression upregulated matrix metalloproteinase (MMP)-3 and MMP-13, which participate in cartilage destruction during OA. Adenovirus-mediated ERRγ overexpression in mouse knee joints or ERRγ transgenic mice resulted in OA.In mouse joint tissues, genetic ablation of Esrrg obscured experimental OA. These results indicate that ERRγ is involved in OA pathogenesis.In mouse articular chondrocytes, GSK5182 inhibited pro-inflammatory cytokine-induced catabolic factors. | 2020 | [50] |
| Quercitrin | Alleviates cartilage extracellular matrix degradation and delays ACLT rat osteoarthritis development. | Significantly decrease MMP13 gene expression and increase collagen Ⅱ gene expression in IL-1β-induced rat chondrocytes and human chondrosarcoma (SW1353) cells.activated the Phosphatidylinositol 3-kinase p110α (p110α)/AKT/mTOR signaling pathway by targeting p110α. | 2020 | [51] |
| Engeletin | In vivo, ACLT-induced knee OA in rats was alleviated by intraarticular injection of engeletin. | Pretreatment with engeletin alleviated TNF-α-induced inhibition of ECM components (collagen II and aggrecan) and upregulation of matrix catabolic enzymes (MMP9 and MMP3). Engeletin ameliorated chondrocyte apoptosis by inhibiting Bax expression and upregulating Bcl-2 expression. Engeletin maintained the mitochondrial membrane potential of chondrocytes and scavenged intracellular ROS by activating the Nrf2 pathway. The NF-κB and MAPK pathways were inhibited by treatment with engeletin. | 2021 | [52] |
| Mithramycin A （MitA） | Intra-articular injection of MitA into mouse knee joint alleviated OA cartilage destruction induced by surgical destabilization of the medial meniscus (DMM). | MitA markedly inhibited expressions of matrix-degrading enzymes induced by pro-inflammatory cytokine interleukin-1&beta; (IL-1&beta;) in mouse primary chondrocytes.MitA significantly impaired the expression of HIF-2α known to be critical for OA pathogenesis. Such reduction in expression of HIF-2α by MitA was caused by inhibition of NF-κB activation, at least in part. | 2018 | [53] |
| Rebamipide | Rebamipide mediates inhibitory effects on cartilage degradation and osteoclastogenesis in TMJ-OA. | Treatment with rebamipide was also found to activate, mitogen-activated protein kinases such as p38, ERK, and JNK to reduce osteoclast differentiation.In the hyperplasia of condylar cartilage, TMJ-OA joints treated with Repapide experienced significant cartilage degradation, excessive apoptosis of chondrocytes, and increased expression of MMP-13 by chondrocytes in a dose-dependent manner compared to carrier-treated TMJ-OA joints. | 2018 | [54] |
| Harpagoside | Anti-inflammatory | Harpagoside did not inhibit the IL-1β-induced activation of NF-κB and C/EBPβ transcription factors but suppressed the IL-1β-triggered induction, phosphorylation, and DNA binding activity of c-FOS, one of the main components of AP-1 transcription factors. Further, harpagoside significantly inhibited the expression of MMP-13 in OA chondrocytes under pathological conditions. siRNA-mediated knockdown of IL-6 resulted in suppressed expression and secretion of MMP-13 directly linking the role of IL-6 with MMP-13 expression. | 2017 | [55] |
| Dihydroartemisinin | DHA decreased OARSI scores and reduced articular cartilage degeneration. | DHA decreased MMP‑13 and VEGF expression in the articular cartilage,DHA decreased the inhibition of sclerostin through reduction of LIF secretion by osteoclasts and, hence, attenuated aberrant bone remodeling and inhibited angiogenesis in subchondral bone, further reducing the progression of OA. | 2021 | [56] |
| Phlomis umbrosa extract (PUE) | PUE treatment improved OA based on decreased joint diameter, increased joint morphological parameters, and histopathological features. | Many genes involved in multiple signal transduction pathway and collagen activation in OA were differentially regulated by PUE. These included genes related to Wnt/β-catenin, OA pathway, and sonic hedgehog signaling activity. Furthermore, PUE treatment downregulated cartilage damage factors (MMP-9, MMP-13, ADAMTs4, and ADMATs5) and upregulated chondrogenesis (COL2A1 and SOX-9) by regulating the transcription factors SOX-9, Ctnnb1, and Epas1. | 2021 | [57] |
| Danshen | Prevention of articular cartilage degeneration in OA. | Danshen can prevent articular cartilage degeneration in OA through the defense against oxidative stress. | 2015 | [58] |
| Kartogenin-conjugated polyurethane nanoparticles (PN-KGN) | IA injection of PN-KGN showed less cartilage degeneration with significant lower OARSI scores even at 12 weeks, indicating PN-KGN could further arrest the development of OA. Immunohistochemistry also validated that IA injection of PN-KGN retained the normal compositions of cartilage matrix, with much stronger Col II staining and less Col I staining. | A small molecular, kartogenin (KGN), was reported to show both regenerative effects and protective effects on cartilage by mediating the CBF-β/Runx1 signaling pathway, Polyurethanes are important amphiphilic polymers and carry urethane or carbamate bonds (–NH–COO–) in their main chains, having been widely used in regenerative medicine and drug-controlled release due to good biocompatibilities and tailorable molecule structures. | 2018 | [59] |
| MicroRNA-140 | Alleviates osteoarthritis (OA) progression by modulating extracellular matrix (ECM) homeostasis | Overexpressing miRNA-140 in primary human chondrocytes promoted Collagen II expression and inhibited MMP-13 and ADAMTS-5 expression. miRNA-140 levels in rat cartilage were significantly higher in the miRNA-140 agomir group than in the control group. Moreover, behavioural scores, chondrocyte numbers, cartilage thickness and Collagen II expression levels in cartilage were significantly higher, while pathological scores and MMP-13 and ADAMTS-5 expression levels were significantly lower in the miRNA-140 agomir group than in the control group. | 2017 | [60] |
| Morroniside | Attenuated the progression of OA in mice, resulting in substantially reduced osteophyte formation and subchondral sclerosis and lower OARSI scores,moronidine has a protective effect against the degradation of cartilage matrix | Morroniside significantly promoted cartilage matrix synthesis by increasing collagen type II expression and suppressing chondrocyte pyroptosis. Morroniside administration led to inhibition of matrix metalloproteinase-13 (MMP13), Caspase-1 and nod-like receptor protein-3 (NLRP3) expression in DMM mice and IL-1β-stimulated chondrocytes. In addition, morroniside attenuated the progression of OA by enhancing chondrocyte proliferation and inhibiting chondrocyte apoptosis. Morroniside also attenuated the progression of OA by inhibiting nuclear factor-κB (NF-κB) signaling. | 2020 | [61] |
| Flurbiprofen sustained release thermoge | This sustained drug release system exerted comparable short-term analgesic effects and distinctly improved long-term analgesic efficacy in terms of the increased percentage of the total ipsilateral paw print intensity and the reduced Knee-Bend scores of OA rats. | The inflammatory response was attenuated in the samples of flurbiprofen gel treated group by showing decreased IL-1, IL-6, and IL-11 levels in the joint fluid and down-regulated IL-1, IL-6, IL-11, COX-2, TNF-α, and NF-κB/p65 expression in the articular cartilages. | 2020 | [62] |
| Tri-butanoylated N-acetyl-D-galactosamine analog (3,4,6-O-Bu3GalNAc) | The dual effects promoted chondrogenesis of human MSC and reduced inflammation of human OA chondrocytes in in vitro cultures. | Induced cartilage tissue production by human mesenchymal stem cells (hMSCs) and human OA chondrocytes by modulating Wnt/β-catenin signaling activity. Wnt signaling pathway has been shown to not only improve chondrogenesis but also impair the osteogenic capacity of MSCs | 2015 | [63] |
| Phloretin | Treatment with phloretin not only prevented the destruction of cartilage and the thickening of subchondral bone but also relieved synovitis in a mouse model of OA. | Phloretin significantly inhibited the IL-1β-induced production of NO, PGE2, TNF-α, and IL-6, the expression of COX-2, iNOS, MMP-3, MMP-13, and ADAMTS-5, and the degradation of aggrecan and collagen-II in human OA chondrocytes. Furthermore, phloretin dramatically suppressed the IL-1β-stimulated phosphorylation of PI3K/Akt and activation of NF-κB in human OA chondrocytes.Moreover, immunohistochemical results showed that phloretin significantly decreased the expression of MMP-13 and increased the expression of collagen-II in OA in mice. | 2018 | [64] |
| Cannabidiol (CBD) | Topical administration of CBD prevented OA pain.Prophylactic CBD treatment prevented the later development of pain and nerve damage in these OA joints. | Osteoarthritis was induced in male Wistar rats (150-175 g) by intra-articular injection of sodium monoiodoacetate (MIA; 3 mg). On day 14 (end-stage OA), joint afferent mechanosensitivity was assessed using in vivo electrophysiology, whereas pain behaviour was measured by von Frey hair algesiometry and dynamic incapacitance. To investigate acute joint inflammation, blood flow and leukocyte trafficking were measured on day 1 after MIA. Joint nerve myelination was calculated by G-ratio analysis. The therapeutic and prophylactic effects of peripheral CBD (100-300 μg) were assessed. In end-stage OA, CBD dose-dependently decreased joint afferent firing rate, and increased withdrawal threshold and weight bearing (P < 0.0001; n = 8). | 2017 | [65] |
| Clodronate | Prevention of cartilage damage, synovial hyperplasia, and proteoglycans loss; reduction in joint inflammation, joint swelling, and osteophyte formation. | Several effects of clodronate have been observed in animal models of OA, including depletion of synovial lining cells that results in reduced production of chemokines (IL-1, TNF- α), growth factors (TGF-β, BMP 2/4), and metalloproteases (MMP 2/3/9); | 2021 | [66] |
| Gypenoside (GP) | Anti-inflammatory | GP dose-dependently inhibited IL-1β-induced NO and PGE2 production in human OA chondrocytes. In addition, treatment of GP inhibited the expression of MMP3 and MMP13, which was increased by IL-1β. Finally, we found that pretreatment of GP obviously suppressed NF-κB activation in IL-1β-stimulated human OA chondrocytes. Taken together, the results demonstrated that GP has chondro-protective effects, at least in part, through inhibiting the activation of NF-κB signaling pathway in human OA chondrocytes. | 2017 | [67] |
| C-phycocyanin (c-PC) | Protect cellular components against oxidative stress, along with its anti-inflammation and anti-apoptosis effects | C-PC had the ability to inhibit ROS production, reverse caspase-3 activity, and reduce apoptosis cell population. C-PC also reversed aggrecan and type II collagen gene expressions after stimulation with 1mM H2O2 or 60psi of compression. Inhibition of IL-6 and MMP-13 genes was observed in compression-stimulated chondrocytes but not in H2O2-treated cells. In dimethylmethylene blue assay and alcian blue staining, C-PC maintained the sulfated-glycosaminoglycan (sGAG) content after stimulation with compression. | 2016 | [68] |
| MicroRNA-34a-5p (miR-34a-5p) | Increased levels of miR-34a-5p have joint destructive effects and can be targeted using ASO technology to attenuate cartilage degeneration during OA. | MiR-34a-5p contributes to synovial pathology by promoting the expression of key inflammatory, ECM, profibrotic, and autophagy markers in OA FLS. In OA, miR-34a-5p may play a role in synovitis by increasing autophagy gene expression. | 2021 | [69] |
| Progranulin (PGRN) | It weakened the degradation of cartilage matrix and inhibited inflammation | PGRN activated extracellular signal-regulated kinases (ERK) 1/2 signaling and elevated the levels of anabolic biomarkers in human chondrocyte, and the protective function of PGRN was mediated mainly through TNF receptor 2. Additionally, PGRN suppressed inflammatory action of TNF-α and inhibited the activation of β-Catenin signaling in cartilage and chondrocytes. | 2021 | [70] |
| Isoimperatorin (Iso) | Iso significantly ameliorated the severity of articular cartilage degradation in mice with experimental OA. | The expression levels of MMP13, Runx2, Col X and VEGF were reduced in Iso‑treated mice. In murine primary chondrocytes, Iso also reduced MMP13, Runx2, Col X and VEGF expression, and activated autophagy by downregulating the mTOR complex 1 (mTORC1) signaling pathway. Therefore, the results of the present study demonstrated that Iso ameliorates OA‑induced pathological alterations by delaying chondrocyte deterioration, activating autophagy and inhibiting mTORC1, which suggests that Iso may have therapeutic potential for attenuating articular cartilage degradation and treating OA. | 2017 | [71] |
| Ketamine | Intra‑articular injection of ketamine ameliorated the pathological characteristics of OA,suppresses the inflammatory response | Decreased TNF‑α and NF‑κB p65 expression levels, and increased the level of IL‑10 expression in a dose-dependent manner. | 2016 | [72] |
| Curcumin | Administration of curcumin significantly reduced osteoarthritis disease progression in DMM model of osteoarthritis. | Curcumin suppressed mRNA expression of proinflammatory mediators in arthrodial cartilage of mice subjected to surgery. In LPS- and ATP-induced THP-1 macrophage cells, curcumin significantly suppressed the expression of interleukin 1 beta (IL-1β) and tumor necrosis factor alpha (TNF-α) at both RNA and protein levels. Compared to vehicle-treated controls, curcumin also showed remarkably increased pro-caspase-1 and decreased cleaved caspase-1. | 2017 | [73] |
| Oroxylin A (ORA) | Inhibits cellular inflammation and hypertrophy of human chondrocytes | Maintained ECM hemostasis of chondrocytes by Interleukin-1β (IL-1β) stimulation. IL-1β induced over-production of inflammatory mediators was attenuated by ORA treatment. Moreover, ORA could rescue IL-1β mediated hypertrophic alterations of chondrocytes. Mechanistically, ORA's protective effects were found to be associated with both NF-κB and Wnt/β-catenin signaling inhibition. Meanwhile, molecular docking analysis revealed that ORA could strongly bind to the inhibitor kappa B kinaseβ (IKKβ) and dishevelled, Dsh Homolog 2 (Dvl2), the upstream molecules of the NF-κB axis and β-catenin axis, respectively. | 2021 | [74] |

**S Table 3: Application of different cells, different materials and different factors in cartilage tissue engineering.**

| **Cell** | **Factor** | **Material** | **Animal** | **Application location** | **Application effect** | **Time** | **Refs.** |
| --- | --- | --- | --- | --- | --- | --- | --- |
| BMSC |  |  | Human | Knee joint | Anti-inflammatory, antifibrotic, improved clinical efficacy, BM-MSCs reduce synovial inflammation in OA. | 2019 | [75] |
| BMSC |  | MSCs with or without platelet-rich plasma (PRP); corticosteroid | Human | Knee joint | MSCs group showed significant differences; The MSCs and MSCs + PRP groups showed the highest percentage of improvement; a significant reduction in intra-articular levels of human interleukin-10 cytokine | 2019 | [76] |
|  |  | Bonelike^®^（HA) | Human | Medial compartment of the knee | New bone; effective vascularization and bone ingrowth; | 2008 | [77] |
| Rabbit adipose-derived mesenchymal stem cells (ADSCs) |  | Biodegradable piezoelectric poly (l-lactic acid) (PLLA) nanofiber scaffold | New Zealand white rabbits | Articular cartilage surface at the medial femoral condyle | Promote chondrogenesis and cartilage regeneration; promoted extracellular protein adsorption; facilitated cell migration or recruitment; induced endogenous TGF-β via calcium signaling pathway; improved chondrogenesis and cartilage regeneration; cartilage regeneration and completely healed cartilage | 2022 | [78] |
| Chondrocytes, infrapatellar fat pad-derived mesenchymal stromal cells (IFP-MSCs） |  | A sulfated carboxymethylcellulose-based scaffold mediated delivery of tissue inhibitor of metalloprotease 3 (Timp3) | Goat, human | Human Osteoarthritis (OA) explants | This scaffold-mediated delivery of Timp3 demonstrated a reduction in matrix degradation, protease expression and inflammatory markers in the human ex vivo OA model leading to enhanced retention of cartilage ECM markers when compared to OA control. | 2022 | [79] |
| BM-MSCs |  | Sodium hyaluronate (SH)/ graphene oxide (GO)/Chitosan (CS)/Nano-hydroxyapatite(nHAP) scaffolds | New Zealand Rabbit | Knee patellar | As can be seen from the 16-week repair diagram, the defect area of the control and experimental group has been completely covered by the new tissue. Compared with the 12-week repair effect, the surface of the experimental group has a good fusion between the new tissue and the host boundary. The contour line has basically disappeared and the surface is relatively smooth and flat. | 2021 | [80] |
| Rat chondrocytes, synovium-derived mesenchymal stem cells (SMSCs) | TET1 | An injectable bioactive self-assembling peptide nanofiber hydrogel | SD rat | Knee joints | The total synovitis scores in knees in the SKP@miR group at 7 and 10 weeks were comparable to those in the sham group, significantly lower than those in the PBS, miR, and SKP groups. The thickening of the synovial lining layer was significantly relieved with SKP@miR relative to PBS. Histological scoring that grades the microscopic structure of the repair cartilage revealed obvious improvements in cell morphology, matrix staining, and cartilage appearance in the SKP@miR group, which was comparable to that in the sham group. | 2022 | [81] |
| Rat adipose mesenchymal stem cell (AMSC) | IL-1β | Chitosan oligosaccharides (COS), extracellular vehicles (Evs), extracellular vesicles-chitosan oligosaccharide conjugates (EVsCOS/EVs-COS conjugates) | Wistar rats | Articular cartilage | IL-1β treatment significantly inhibited the viability and migration of chondrocytes and enhanced cell apoptosis (P < 0.05), while chitosan oligosaccharides and extracellular vesicles-chitosan oligosaccharide conjugates (EVsCOS/EVs-COS conjugates) reversed the changes induced by IL-1β (P < 0.05), and the effects of extracellular vesicleschitosan oligosaccharide conjugates were better than those of chitosan oligosaccharides (P < 0.05). | 2021 | [82] |
| Chondrocytes | Fibroblast growth factor-18(FGF18) | Hyaluronan | SD rat | Meniscus of the knee joint | FGF18-induced dose-dependent increases in cartilage thickness of the tibial plateau,The highest dose of FGF18 also induced an increase in chondrophyte size and increased remodeling of the subchondral bone.Significant tibial cartilage degeneration, where chondrocyte and matrix loss extended through greater than half of the cartilage thickness, was reduced 57% by treatment with the 5.0 μg doses of FGF18. Similarly, treatment with FGF18 produced a 46% dose-dependent reduction in the depth of cartilage lesions. Relative to sham-injected rats, the vehicle alone had little effect on cartilage degeneration scores | 2005 | [83] |
| Chondrocytes | Transforming growth factor-β1 (TGF-β1) and insulin-like growth factor-1 (IGF-1) | Oligo (poly (ethylene glycol) fumarate) (OPF), Gelatin microparticles (MPs) | New Zealand white rabbits | medial femoral condyle | However, when compared to untreated defects, TGF-b1 delivery, as well as TGFb1&IGF-1 co-delivery, only improved scores for the GAG and cell content of the cartilage surrounding the defect.These results suggest the potential of IGF-1 delivery strategies in chondral repair. | 2007 | [84] |
| Chondrocytes |  | Chondrocytes were transferred into bioreactors | Yucatan minipigs | Patellar groove of the femoral condyle | Implant loosening had occurred in 55% of the CD, 20% of the OCS, and 8% of the FT sites. Of these latter 8%, only mild fibrillation of the surface was apparent, mainly because of an infiltration of the surface by inflammatory cells. The control defects had a higher percentage of surface fibrillation, with no inflammatory infiltration of the surface. The thickness of the repair cartilage was 75% that of the normal adjacent cartilage in the case of CD and OCS repairs. | 2001 | [85] |
| Chondrocytes（horse） | TGF-ß1 | A gelatin-methacrylamide  hydrogel |  | Porcine knee joints full-thickness cartilage (Intact), porcine knee joints surface layer (Less STZ) | Similar production of sulphated glycosaminoglycans and collagen II was observed for the novel composite constructs cultured under mechanical conditioning w/o TGF-ß1 supplementation and in static conditions w/TGF-ß1 supplementation, which confirmed the capability of the novel composite construct to support neo-cartilage formation upon mechanical stimulation. | 2019 | [86] |
| BM aspirate concentrate (BMAC), BM-MSC |  | Ultrapurified alginate (UPAL) gel, bone marrow aspirate concentrate (BMAC) | New Zealand white rabbits | Osteochondral of joint | The histologic scores 16 weeks were significantly higher in the UPAL-BMAC group (24.4 6 1.7) than in the Defect group (9.0 6 3.7; P ＜0 .05), the UPAL group (14.2 6 3.9; P＜0 .05), and the UPAL-MSC group (16.3 6 3.6; P＜0 .05).At 4 and 16 weeks, the values of repaired subchondral bone volumes in the UPAL-BMAC group were significantly higher than those in the Defect and UPAL groups. | 2021 | [87] |
| The bone marrow-derived normal human mesenchymal stem cells (BM-MSCs) |  | Elastin–gelatin–hyaluronic acid (EGH) scaffolds | New Zealand white rabbits | Nasal septal cartilage | After a 4-month healing period, computed tomography (CT) and magnetic resonance imaging (MRI) scans were obtained from the nasal septal cartilage, followed by histological evaluations of new tissue formation. Maximum regeneration occurred in Group received EGH scaffolds implanted, according to CT, and Group received EGH scaffolds seeded with autologous auricular chondrocytes implanted, according to both T1 and T2 images with 7.68 ± 1.36, 5.44 ± 2.41, and 8.72 ± 3.02 mm2 defect area respectively after healing. | 2021 | [88] |
| Chondrocytes, M0 macrophages | TNF-α, IL-1β, TGF-β, IL-10, Runx2 | Porcine-derived auricular and costal cartilage | Bama mini pigs,New Zealand rabbits | Costal cartilage | Two types of decellularized cartilage, par- ticularly decellularized auricular cartilage, promoted the tissue regeneration in the cartilage defect area, combined with noticeable cartilage morphology and increased chondrogenic gene expression. | 2021 | [89] |
| MSCs | IGF-1 | Goat conchal cartilaginous ECM derived scaffolding | New Zealand white rabbits | Muscles in the middle of the chest and waist | In the presence of treated cartilage samples, the amount of secreted ECM proteins (collagen and sGAG) increased and MSCs successfully underwent chondrogenic differentiation.Upon implantation of the constructs in rabbits’ osteochondral defects for 3 months, the histological and micro-CT evaluation revealed significant enhancement and regeneration of neocartilage and subchondral bony tissues. The IGF-1 loaded cartilaginous constructs showed comparatively better healing response after 3 months. | 2021 | [90] |
| BMSCs(Autologous） |  | Type II collagen sponge, calcified cartilage zone (CCZ), subchondral bone of pig | Minipig | Cochlear surface | The defects in the blank control and non-CCZ groups were filled with fibrous tissue, while the cartilage layer of the CCZ group was mainly repaired by hyaline cartilage at 24 weeks postoperatively. The superior repair outcome of the CCZ group was confirmed by MOCART and O’Driscoll score. | 2021 | [91] |
| MSCs | TGF-β1 | Adipose-derived MSCs, ear MSCs, bone marrow-derived MSCs | New Zealand white rabbits | Auricle | Histopathologically, defective areas of control positive group, ADMSCs and EMSCs treated groups experienced a small area of immature cartilage. While BMMSCs treated group exhibited typical features of new cartilage formation with mature chondrocytes inside their lacunae and dense extracellular matrix (ECM). BM-MSCs showed the highest chondrogenic potential compared to ADMSCs and EMSCs. | 2022 | [92] |
| Chondrocytes |  | hyaluronic acid (HA)-based cryogel scaffolds,glycidyl methacrylate |  | articular cartilage | Chondrocytes seeded within cryogels and cultured for 15 days exhibited enhanced cell proliferation, metabolism and production of cartilage extracellular matrix glycosaminoglycans compared to in HA-based hydrogels.Furthermore, immunohistochemical staining revealed production of collagen type II from chondrocyteseeded cryogels, indicating the maintenance of cell phenotype. | 2021 | [93] |
|  | NELL-1 | Chitosan nanoparticles,alginate hydrogels | New Zealand white rabbits | Femoral condylar cartilage | Histology of NELL-1-treated defects closely resembled that of native cartilage, including stronger Alcian blue and Safranin-O staining and increased deposition of type II collagen and absence of the bone markers type I collagen and Runt-related transcription factor 2 (Runx2) as demonstrated by immunohistochemistry. | 2011 | [94] |
| MSCs | Blood-derived platelet-rich fibrin releasate (PRFr) |  | New Zealand white rabbits | Femoral condylar cartilage | Relative to the MSCs or PRFr group, histological examination demonstrated that the MSCs 1 PRFr group had thicker hyaline-like cartilaginous tissue with normal glycosaminoglycan production. Grading scores revealed that MSCs 1 PRFr injection had better matrix, cell distribution, and surface indices than other groups. | 2016 | [95] |
|  |  | Decellularized cartilage-derived matrix (CDM), calcium phosphate (CaP) | Horse | Femur | At 6 months, micro-CT and histology showed much more limited filling of the defect, but the CaP component of the þP scaffolds was well integrated with the surrounding bone. The repair tissue was fibrotic with high collagen type I and low type II content and with no differences between the groups. There were also no biochemical differences between the groups and repair tissue was much less stiff than normal tissue (P < 0.0001). | 2016 | [96] |
| Autologous adipose-derived stem cells (ADSCs) |  | Decellularized cartilage ECM scaffolds | New Zealand white rabbits | Patellar groove of the femur | After 6 months, the results showed that in repaired with the chondrogenically induced ADSC–scaffold constructs (group A) were post-surgery most of the repair site was filled with hyaline cartilage. Indeed, histological grading score analysis revealed that an average score in group A was higher than in group only scaffold and controls. GAG and type II collagen content and biomechanical property detection showed that the group A levels approached those of normal cartilage. | 2012 | [97] |
| Chondrocytes,BMSC |  | In situ self-assembling gel based on glyceryl monooleate (GMO)-hyaluronic acid (HA) composite lyotropic liquid crystal (HLC) | Sprague-Dawley (SD) rats | Femur | Compared to the GMO based (LLC) gel, HLC gel with modified lattice structure exhibited improved rheological properties for better joint protection by increasing mechanical strength, elasticity and lubrication.Pharmacodynamic studies demonstrated that HLC gel was the most effective to promote chondrogenesis and protect subchondral bone, making the damaged bone tissue restored to normal in divergent features as evidenced by the MRI, Micro-CT and histological results. | 2021 | [98] |
| autologous endothelial progenitor cells（EPC） | TGF-β2, TGF-β3 | poly(lactide-co-glycolide) (PLGA) scaffold | New Zealand White male rabbits | medial femoral condyle. | The EPC-PLGA group showed the development of new cartilage tissue with a smooth, transparent and integrated articular surface. At week 4, the EPC-PLGA group showed considerably higher TGF-β2 and TGF-β3 expression, a greater amount of synthesized glycosaminoglycan (GAG) content, and a higher degree of osteochondral angiogenesis in repaired tissues. At week 12, the EPC-PLGA group showed enhanced hyaline cartilage regeneration with a normal columnar chondrocyte arrangement, higher SOX9 expression, and greater GAG and collagen type II (COLII) content. Moreover, the EPC-PLGA group showed organized osteochondral integration, the formation of vessel-rich tubercular bone and significantly higher bone volume per tissue volume and trabecular thickness (Tb.Th).EPC-PLGA cell delivery system generates a suitable in situ microenvironment for osteochondral regeneration without the supplement of exogenous growth factors. | 2013 | [99] |
| MSCs |  | fibrin glue as a scaffold | human | OA knees | ①At final follow-up (mean, 28.6 months; range, 24-34 months), the mean IKDC score and Tegner activity scale ： from 36.1 ± 6.2 to 64.4 ± 11.5 (IKDC) and from 2.2 ± 0.8 to 3.8 ± 0.8 (Tegner). ②According to the overall ICRS cartilage repair grades, 12 of the 17 lesions (58%) achieved a grade of I（normal) or II(near normal). | 2015 | [100] |
| Synovial MSCs |  | tissue-engineered construct（scaffold-free ） | human | knee chondra:medial femoral condyle, lateral femoral condyle, or femoral groove | No serious adverse events were observed out to 2 years after TEC implantation.the Lysholm score, the Tegner activity score, and all subcategories of the KOOS were significantly improved by 48 weeks and remained high out to 2 years.Based on MRI assessments, cartilage defects filled with newly generated tissues over time, and the defect filling rate reached 100% coverage without detectable hypertrophy of the repair tissues by 48 weeks for all patients.The repair tissue exhibited good tissue integration with adjacent host cartilage.the autologous TEC was composed of only cells and matrix from the individual patient, suggesting a higher safety profile with possibly lower production costs as compared with other tissue-engineered cartilage approaches with exogenous biomaterials.TECs enable the readily matching to the needed shape for repair of a chondral defect.TEC promotes the safety and effectiveness of cartilage repair. | 2018 | [101] |
| Chondrocytes |  | Esterified hyaluronic acid scaffolds (Hyalograft ®C) | Human | Knee articular cartilage | Hyaline cartilage regeneration can be observed less than 1 year after implantation of Hyalograft®C and that there is progressive maturation of the implants, even in joints showing signs of osteoarthritis.tissue regeneration can occur when cartilage lesions are treated using Hyalograft C tissue engineering.This approach will allow us to improve further the outcome of cartilage repair. | 2006 | [102] |
| Human chondrocytes |  | 2:2:3(w/v) chitosan (C), gelatin (G) and silk fibroin (S) | Rabbits | Knee articular cartilage | After eight weeks of implantation,The result showed the least amount of fibroblast (6.0 ± 1.6). the number of chondrocyte cells was significantly high.made up the most number of cells in the generated tissue (52.7 ± 2.8). the number of osteocyte cells was also significantly high (42.0 ± 6.2) but less than the normal cartilage (46.5 ± 7.0). Rabbits with cell-seeded scaffolds had better locomotion performance. CGS 2:2:3 could be an appropriate scaffold for cartilage tissue engineering. | 2021 | [103] |
| Bone marrow mesenchymal stem cells(BM-MSCs) | TGF-β1、IGF-1 | Platelet-rich fibrin glue (PR-FG) | Human | Full-thickness cartilage defects of femoral condyles | ICRS arthroscopic scores were 8/12 and 11/12 (nearly normal) for the 2 patients who consented to arthroscopy. MRI of 3 patients at 12 mo postoperatively revealed complete defect fill  and complete surface congruity with native cartilage, whereas that of 2 patients showed incomplete congruity. Autologous BM-MSC transplantation on PR-FG as a cell scaffold may be an effective approach to promote the repair of articular cartilage defects of the knee in human patients. | 2010 | [104] |
| Endogenic bone marrow stem cells (BMSCs) |  | Integrating silk fibroin with gelatin | Adult male New Zealand white rabbits | Knee articular cartilage | A superior chondrogenic differentiation ability of the BMSCs cultured within the scaffold. neo-cartilage in the SFG-E7 group was more similar to normal cartilage.It has also shown increased expression of hyaline-cartilage-specific gene collagen type II , while the expression of fibrotic marker gene collagen type I and hypertrophic marker gene collagen type X was low.the level of hydroxyprolin (HYP) (indicating collagen content) and GAG significantly increased over time.It appears to be a promising biomaterial for knee cartilage repair. | 2017 | [105] |
| Mesenchymal stem cells (MSCs) |  | Hyaluronan-based scaffold (Hyaff®11) | New Zealand adult male rabbits | Knee joints | The tissue surfaces treated become thicker during the experimental times evaluated compared to the untreated cartilages. Cartilages show a better matrix organization, a higher presence of proteoglycan component, and anormal distribution of the cells.The MSCs-HA group showed positive immunostaining for collagen type II, which was evident at extracellular and cellular levels after 3 and 6 months of implantation.A cartilage tissue regeneration was evident in the MSC-HA–treated group particularly at 6 months. | 2008 | [106] |
| Bone marrow-derived mesenchymal stem cells |  | Silk fibroin/chitosan scaffold | New Zealand rabbits | Knee joints | The water absorption of the scaffold was 143.15 ± 5.97 %; elastic modulus 28.10 ± 1.58 MPa, and the compressive strength was 0.65 ± 0.02 Mpa.the cells were positive for type II collagen in BMSCs +SF/CS scaffold.At 3 days, a large number of cells had adhered to the surface and pores of the scaffold. Cells grew well and actively proliferated. At 8 weeks post-surgery, defects remained filled with newly generated tissues such as cartilage in the BMSCs + SF/CS scaffold group. The repair surface remained smooth, and the boundary between newly generated tissues and normal cartilage was not obvious.BMSCs +SF/CS scaffold promoted osteochondral starvation repair. | 2013 | [107] |
| bone marrow-derived mesenchymal stem cells (BMSCs) |  | poly(L-lactide-co-Ɛ-caprolactone) | mature New Zealand white rabbits | medial femoral condyles on a rabbit’s knee joint | At 6 month-implantation, the defects were completely filled with neocartilage tissues near the surface and regular subchondral bone formation at the bottom. There was close integration of cartilage and bone, with a calcified cartilage layer at the interface bordered by a tidemark, which was comparable to normal cartilage.In the current study, PLCL scaffold seeded with BMSCs showed significant improvement in cartilage and bone regeneration. | 2009 | [108] |
| adipose derived stem cells(ASCs) |  | fibrous polyglycolic acid (PGA) scaffold stabilized with polylactic acid (PLA) | pig | knee joints | A vivid remolding process with post-operation time was also witnessed in the neo-cartilage as its compressive moduli increased significantly from 50.55% of the normal cartilage at 3 months to 88.05% at 6 months.both COL II and GAG contents underwent a robust increase during the first week and maintained at a high level within the following test duration.a nice interface with excellent healing was achieved between the engineered cartilage with both its adjacent native cartilage and subchondral bone at either 3 or 6 months post-implantation. ASCs are capable of differentiating into chondrocyte-like cells in vitro under specific culture conditions and further maintaining the chondrogenic phenotype in vivo.the successful repair substantiates the potential of using chondrogenic induced ASCs and PGA/PLA scaffold for cartilage regeneration. | 2009 | [109] |
| Cartilage-derived mesenchymal stem cells (C-MSCs), synovial membranederived mesenchymal stem cells (SM-MSCs) | Stromal cell-derived factor-1 (SDF-1) | Type 1 collagen scaffold | Rabbit | Knee joints | The average ICRS macroscopic score of the partial-thickness defects group (4.91 ± 0.38) was statistically lower than that of the full-thickness defects group (14.50 ± 0.87) at 6 weeks post-injury. The histological score for cartilage evaluation showed significantly enhanced cartilage repair in full-thickness defects (1 1.78 ± 0.51) compared with partial-thickness defects (4.22 ± 0.84, p < 0.05). There were much fewer attached MSCs on partial-thickness defects than that on full-thickness defects. Quantitative analysis confirmed this observation (C-MSCs: 24.63 ±2.97 vs. 66.40 ± 7.79, p < 0.05; SM-MSCs:37.00 ± 5.29 vs. 60.60 ± 16.24, p < 0.05). The combination of SDF-1 and col1, which mimicked subchondral bone matrix environment, improved the repairing effect of partial-thickness defects. | 2012 | [110] |
| BMSC | stromal cell-derived factor-1 (SDF-1) | Ultrapurified Alginate Gel Containing (UPAL) | rabbit | knee joints | SDF-1 enhanced the repairing effect of osteochondral defects through increasing migration of host cells (mainly BMSCs) to the defect region, indicating SDF-1 is a potent candidate for cartilage repair. The scores at 16 weeks after operation were significantly higher in the SDF-1 group (UPAL+SDF-1) than in the other groups. | 2012 | [111] |
| Autologous Mesenchymal Stem Cells |  | β-Tricalcium Phosphate | Sheep | Knee joints | Twelve weeks postoperation, the defects that were treated with tissue-engineered cartilage in the experimental group were mostly repaired, resulting in a relatively smooth and consistent joint surface. The mean histological scores were 14 ± 1.70 (SD) 12 weeks postimplantation) and 18 ± 3.38 (SD) (24 weeks postimplantation) in the experimental group, 10 ± 3.40(SD) (12 weeks postimplantation) and 10 ± 1.83 (SD) (24 weeks postimplantation) in control group 1, and 5 ±1.83 (SD) (12 weeks postimplantation) and 5 ± 2.08(SD) (24 weeks postimplantation) in control group 2. | 2004 | [112] |
| Gene-engineered ADSCs | TGF-β1 | An injectable extracellular matrix (ECM)-mimicking hydrogel | Male Sprague-Dawley (SD) rats | Knee joints | The gene-engineered ADSCs overexpressing TGF-β1 (T-ADSCs) had an enhanced paracrine effect on OA-like chondrocytes, which effectively decreased the expression of tumor necrosis factor-alpha and increased the expression of collagen II and aggrecan. In a surgically induced rat OA model, intra-articular injection of the T-ADSC-loaded hydrogel markedly reduced cartilage degeneration, joint inflammation, and the loss of the subchondral bone. Taken together, this study provides a potential biomaterial strategy for enhanced OA treatment by delivering the gene-engineered ADSCs within an ECM-mimicking hydrogel. | 2021 | [113] |
| Adipose-derived stem cells (ADSCs) | MCP-1, ICAM-1, TIMP-1 | Biomimetic injectable hydrogel using amnion membrane (AM) | Male Sprague-Dawley rats | Knee joints | At 28 d posttreatment, ADSC (0.8 ± 0.3, P < 0.05), AM gel (0.5 ± 0.1, P < 0.0001), and AM-ADSC (0.4 ± 0.1, P < 0.0001) treatment groups showed a significant decrease in joint diameter compared to the control group (1.4 ± 0.8). Importantly, the knee diameter in the AM-ADSC group was found to be significantly lower than ADSC (P < 0.001) and AM groups (P < 0.05), indicating decreased synovial inflammation in the combination group AM-Adson day 21 the AM-ADSC group (5,778 pg/mL ± 549) showed a significant decrease (P < 0.05) in ICAM-1 compared to the control group (8,641 pg/mL ± 1,439).A significant decrease in MCP-1 levels was also noted in AM-ADSC group (5,089 pg/mL ± 810) compared to control groups (7,748 pg/mL ± 304, P < 0.0001) .The AM-ADSC group further showed a significant reduction of ICAM- 1, leptin, and MCP-1 levels compared to all other groups on day 28 , indicating a synergistic antiinflammatory effect. | 2021 | [114] |
| Adipose tissueederived stem cells | MMP-3, MMP-13, TNF-a, IL-1b | Xanthan gum | Male, eight-week-old Wistar rats, male, four-week-old Wistar rats | Knee joints | For the rats in the XG-ADSCs groups, the percentages of weight bearing of the right hind limb were increased significantly and lasted for 4 weeks. the positive effect of the XG-ADSCs group was significantly high. In the XG-ADSCs group, the cartilage surface was smooth, and the matrix was well stained by Safranin O. XG-ADSCs treatments significantly lowered the Mankin scores and the density of immuno-localized type II collagen was high.the concentrations of IL-1b, TNF-a, MMP-3 and MMP-13 were significantly decreased in the XG-ADSCs group. | 2017 | [115] |
| matrix-assisted autologous chondrocyte |  | nano-structured porous polycap-rolactone (NSP-PCL) scaffold | mature male New Zealand White Rabbits | femoral intercondylar grooves | The NSP-PCL scaffold demonstrated high in vitro expression of chondrogenic markers and had high in vivo histological scores. The relative expression to the housekeeping genes, of the chondrogenic markers sox9 and aggrecan, was higher in the NSP-PCL scaffold than in the Chondro-Gide scaffold, and the fibrous tissue marker collagen type 1 was lower than in the Chondro-Gide scaffold. the collagen type 2 expressions were higher slightly in the NSP-PCL scaffolds. NSP-PCL scaffold with autologous chondrocytes could provide a less costly and more effective treatment option than Chondro-Gide® scaffold with cells. | 2012 | [116] |
| chondrocytes | SOX9, COL1, COL2, IL-1β | a soluble eggshell membrane/agarose composite scaffold |  |  | a novel composite scaffold with possible application in cartilage tissue regeneration was made by mixing Agr, a well‐known biopolymer used in tissue engineeringand the soluble egg shell membrane powder proved to be biologically active by providing hydrophilic sites to the cells and promoting the chondrocyte differentiation. | 2021 | [117] |
| Elephant chondrocytes | TGF-β1 or IGF-1 | gelatin scaffolds |  |  | This three-dimensional culture model is probably helpful for developing cartilage regeneration in vitro and is further applied in tissue engineering for OA treatment in vivo.TGF-β1 and IGF-1 treatment combined treated scaffolds had ACAN and COL2A1 gene expression higher than those of TGF-β1 or IGF-1 treated alone in some period of time (day 14). mRNA and protein expressions of collagen type II and aggrecan in the stimulation of both TGF-β1 and IGF-1 were higher than TGF-β1 or IGF-1 treated human chondrocytes and mice MSCs in pellet cultures. Moreover, there was greater protein expression of collagen type II and aggrecan, and greater mRNA expression of ACAN and COL2A1 following TGF-β1 and IGF-1 combined treatment, compared with either TGF-β1 or IGF-1 treated human MSCs in monolayer culture. the combined treatment of TGFβ1 and IGF-1 seem to be beneficial in stimulating the activity of cartilage-specific anabolic genes in elephant chondrogenesis. | 2022 | [118] |
| BMSC | TGF-β1 | The surface layer was made from collagen (COL), chitosan (CS) and hyaluronic acid sodium (HAS). The transitional layer with microtubule array structure was prepared with COL, CS and silk fibroin (SF) | male New Zealand white rabbits | knee joints | the optimal ratio of COL/CS/HAS salt porous scaffold was 1:1:0.1. COL/CS/0.5SF scaffold exhibited the highest compressive strength(29.24 ± 0.10 KPa) amongst the four scaffolds.Compared with surface layer scaffolds and transitional layer scaffolds, cells exhibited best proliferation rate in biomimetic cartilage scaffolds on the 7 days. Proliferation results suggested that biomimetic cartilage scaffolds could enhance the cells proliferation.At postoperative 16 weeks, in gross observation, articular surface of biomimetic cartilage scaffold group was smooth. Obviously, compared with the other two groups, the bionic oriented cartilage scaffold group with two factors showed the best regeneration effect. | 2019 | [119] |
| bone marrow concentrate (BMC) and mesenchymal stem cells (MSC) | IL-1β | Hyaff®-11 (hyaluronic acid) | sheep | knee joints | BMC-HA treatment showed a greater repair ability in inhibiting OA progression compared to MSCHA, leading to a reduction of inflammation in cartilage, meniscus, and synovium. Moreover, the treatment with BMC-HA showed the best results in allowing meniscus regeneration. Minor healing effects were noticed at bone level for both cell strategies.The number of nucleated cells counted within the BMC from our samples displayed a high variability ranging from 6.0 · 106 cells/mL to 53.0 · 106 cells/mL, with a mean value of 39.0 · 106 cells/mL.the transplantation of BMCHA showed a higher expression for type II collagen and lower expression for type I collagen compared to the MSC-HA group. BMC-HA treatment showed lower positivity for MMP-13 and IL-1β compared to MSC-HA approach. | 2016 | [120] |
| Autologous adipose mesenchymal stem cells |  | hyaluronic acid | male small tail Han sheep | knee joints | IA injection of autologous ASC+HA may demonstrate better efficacy than autologous SVF+HA in blocking OA progression and promoting cartilage regeneration,and autologous high-dose ASCs (5×107 cells) combined with HA potentially survive for at least 18 weeks after IA injection.12 weeks after the second injection, the autologous ASCþHA and SVFþHA groups all exhibited a more glossy, continuous, and intact layer of articular cartilage than the HA alone and saline alone groups. In particular, effusion of the articular cavity and defects in the cartilage and subchondral bone were obvious in both the HA alone and saline alone groups, demonstrating the efficacy of autologous ASC+HA and SVF+HA therapy. MOCART scores, which were evaluated by three independent observers, demonstrated the significantly increased efficacy of the low-dose ASC+HA treatment compared with the SVF+HA, HA alone, and saline alone groups. | 2018 | [121] |
| Mesenchymal stem cells | Osteochondrogenic factors | A mPEG-block-poly(L-valine) thermogel set and a poly(lactide-co-glycolide)/hydroxyapatite porous scaffold |  | Knee joints | Gross evaluation, micro-computed tomography, and magnetic resonance imaging indicated that at 6 months after implantation, the scaffold encapsulating mesenchymal stem cells and osteochondrogenic factors had a better effect compared with other groups. More importantly, the composition of the new cartilage and bone was confirmed by western blotting, H&E staining, immunohistochemistry, and immunofluorescence. Taken together, this bionic composite scaffold with osteochondrogenic factors offers a promising option for the repair of full-thickness osteochondral defects. | 2022 | [122] |
| BMSCs |  | bilayered poly(lactide-co-glycolide) porous scaffolds | rabbits | knee joints | The bilayered PLGA porous scaffold can facilitate the repair of osteochondral defects and has potential for application in osteochondral tissue engineering.its effect can be further facilitated under the scaffold seeded with allogenic BMSCs. The neotissues in the cell-seeded bilayered scaffolds exhibited better resurfacing than those in the scaffolds without cells 24 weeks after implantation.the repair of the osteochondral defect tends to be stable 12 weeks after implantation.the formed cartilage layer was thicker than the native cartilage at 12 weeks. The thickness of cartilage was about 1.5 mm in the group of scaffold with cells and 1.0 mm in the scaffold group. The thickness of cartilage was almost or less than 0.5 mm at 24 weeks, while the thickness of the normal cartilage was almost 0.3-0.5 mm. After 24 weeks, although the neotissues regenerated by bilayered scaffolds with cells were better than those repaired by bilayered scaffolds without cells, the thickness of the chondral region was declined and closer to that of the native cartilage. | 2019 | [123] |
| Mesenchymal stem cells (MSCs) are found in synovial fluid (SF)----SF-MSCs | TGF-ß1+BMP-2 | Collagen sponges | 5-week-old nude male rats | Knee joints | SF-MSCs were strongly positive for CD73 and CD105 and also positive for CD90.TGF-ß1 + BMP-2 under normoxia and hypoxia, significantly increased SOX9 expression (2.7- vs 5.4-fold).Type II collagen, and particularly its IIB isoform, is specific of hyaline cartilage. Under normoxia, with TGF-ß1 and TGF-ß1 + BMP-2, a significant increase.collagen sponge is a porous biomaterial allowing extracellular matrix synthesis. TGF and BMP are prone to enhance chondrogenesis of SF-MSCs.Our results confirm the boosting effect of TGF-ß1 on some chondrogenic gene expression (ACAN, COL2A1, COMP, SOX9) and extracellular matrix production (GAG, type II collagen) in SF-MSCs seeded collagen sponges on D28. | 2018 | [124] |
| ligament-derived stem/progenitor cells (LSPCs) | Stromal cell-derived factor 1(SDF-1) | (CSF)collagen-silk | Female New Zealand white (NZW) rabbits | Knee joints | At 6 months post-treatment, the CSFL group displayed a more robust regenerated ligament with glossy white appearance and fully-filled structure, which was similar to a native ACL. Histological examination further demonstrated abundant ECM accumulation within the CSFL scaffold.In the CSFL group, the tissues with markedly greater bone density were observed within the defects. Quantification of micro-CT images provided further evidence that significantly more bone was formed with higher BS/TV , BMD, Tb.N, and Tb.Th in the CSFL group than in the CS group. | 2018 | [125] |
| bone marrow derived mesenchymal stem cells |  | collagen/chitosan scaffold | immunocompromised mice | the back of immunocompromised mice.on either side of the spine of the same mouse | Such ECM incorporated scaffolds have great potential in cartilage regenerative therapy. These scaffolds possess key pro-chondrogenic ECM components and growth factors. The scaffolds possess mechanical properties and diffusion characteristics important for cartilage tissue regeneration. In vivo implantation of the chondrogenic ECM scaffolds with bone marrow derived mesenchymal stem cells (MSCs) triggered chondrogenic differentiation of the MSCs without the need for external stimulus. the chondrogenic ECM scaffolds are stable and possess MR properties on par with native cartilage. | 2015 | [126] |
| MSCs | TGF-β1 | Methoxy poly (ethylene glycol)-block-poly(ε-caprolactone) having terminal groups of carboxylic acid.hydroxyapatite (HAp).amino groups of the Arg-Gly-Asp(RGD) peptide. hydrogel of glycidyl methacrylate hyaluronic acid. | Male New Zealand white rabbits | Knee joints | The bone and cartilage defects produced in the knees were fully healed 12 weeks after the implantation of the TGF-β1 loaded hydrogel and scaffolds, and regenerated cartilage was hyaline cartilage.HAp significantly increase the compressive strength of the porous scaffolds.the osteoblast-like cells and stem cells well spread on RGD-modified PEG-PCL film indicating a favorable surface for the proliferation of cells. | 2017 | [127] |
| Juvenile bovine mesenchymal stem cells (jbMSCs) | Stromal Cell-Derived Factor-1α (SDF-1α; SDF)/Transforming Growth Factor- β3 (TGF- β3; TGF) | Fibrous hyaluronic acid (HA) | Castrated male juvenile Yucatan minipigs | Knee joints | Cell infiltration was assessed by seeding MSCs onto scaffolds and measuring the depth of cell penetration. Growth fac- tor delivery noticeably increased cellular infiltration in SDF, TGF, and S + T groups. The greatest increase was from 77 + /- 20 μm in the Scaffold group to 269 + /- 90 μm in the TGF group.To assess the chondrogenic activity of scaffolds, MSC pellet s were cultured with either soluble-delivered or scaffold-delivered biofactors.Both groups with TGF- β3 (TGF, S + T) trended towards increased GAG and DNA content com- pared to groups without TGF- β3 (Media, Scaffold, SDF). Pellets co-cultured with S + T scaffold produced significantly more GAG than pellets co-cultured with SDF or factor-free scaffolds.TGF- β3 trended towards inducing more cell infiltration, proliferation, and chondrogenesis compared to SDF-1 α.TGF- β3 augmented cartilage repair. | 2021 | [128] |
| MSC | Anti-inflammatory cytokines | (polycaprolactone) PCL scaffolds |  |  | The ability of functional engineered cartilage to deliver tunable anti-inflammatory cytokines to the joint may enhance the long-term success of therapies for cartilage injuries or osteoarthritis.significantly higher levels of IL-1Ra than all other conditions for the duration of the experiment (~100-1000 ng/mL IL-1Ra).IL-1Ra-expressing constructs maintained rich GAG staining at 0.1 ng/mL IL-1 and reduced staining was observed at 1 ng/mL IL-1, corroborating the quantitative biochemical results that show IL-1Ra overexpression markedly improves GAG deposition in engineered cartilage constructs exposed to IL-1.obtain the equilibrium Young’s modulus (EY) of samples either 1 h following seeding, at the time of chondrogenic induction, or after 27 days of chondrogenesis with or without IL-1. EY averaged approximately 1 MPa in all groups and was not affected by tissue production during chondrogenesis or treatment with IL-1. | 2014 | [129] |
| Autologous chondrocytes |  | Hyaluronic acid/polycaprolactone material | Skeletally mature female adult sheep (Bergamasca– Massese) | Knee joints | Seeding of the scaffold with autologous chondrocytes increases its tissue regeneration capacity, providing a better fibrocartilaginous tissue formation. The study suggests the potential of the novel hyaluronic acid/polycaprolactone scaffold for total meniscal substitution, although this approach has to be further improved before being applied into clinical practice.The implant gross assessment showed significant differences between cell-seeded and cell-free groups.Joint gross assessment showed that sheep treated with scaffold implantation achieved a significant higher score than those underwent meniscectomy, and the Osteoarthritis Research Society International score showed that osteoarthritic changes were significantly less in the cell-seeded group than in the meniscectomy group.Osteoarthritis was evident histologically in all joints. This was most severe in the meniscectomy-alone group with an OARSI score of 12.0 – 3.0. In contrast, the OA changes were less severe in the cell-seeded group with an OARSI score of 8.7 – 2.3. The OA score of the cell-free group was also less being 9.7 – 3.5. | 2012 | [130] |
|  | Growth factor: connective tissue growth factor (CTGF) +transforming growth factor β3 (TGF-β3) | Poly-Ɛ-caprolactone (PCL) scaffolds. | Sheep | Knee joints | All experimental groups exhibited articular cartilage degeneration as compared with control (nonoperated). In terms of synovitis, there were no clear differences among groups, suggesting that growth factors did not increase inflammation and fibrosis. MRI revealed that meniscal extrusion was observed in most animals (82.7%). The combination of CTGF and TGF-β3 can stimulate mesenchymal stem cells into a fibrochondrocyte lineage and did not aggravate synovitis, suggesting no adverse response to the combination of 3D-printed PCL scaffold combined with CTGF and TGF-β3. Further work will be required to improve scaffold fixation to avoid meniscal extrusion.The 5-μg group had the best meniscal matrix organization,with proteoglycan-rich fibrocartilage-like tissue. To a lesser degree, the 10-μg group also yielded proteoglycan-rich regenerated meniscus at 6 months, and the 0-μg group contained only disorganized fibrous tissue, even at 12 months (matrix staining subcategory: 0 μg, 7.0 ± 2.1; 5 μg, 3.9 ± 1.6; 10 μg, 4.9 ± 2.0). | 2019 | [131] |
| Mesenchymal stem cell |  | Polyglycolic acid-hydroxyapatite biphasic scaffold | New Zealand White rabbits | Knee joints | The novel MSCs-seeded, PGA-HA biphasic graft facilitated both articular cartilage and subchondral bone regeneration in an animal model and might serve as a new approach for clinical applications.At 16 weeks postimplantation, significant integration of the newly formed tissue with surrounding normal cartilage and subchondral bone was observed when compared to the two control groups. At 32 weeks, no sign of progressive degeneration of the newly formed tissue was found.At a higher magnification, the thickness of the regenerated cartilage was observed to be about 2/3 of the surrounding normal cartilage. No residual PGA fiber was found in the implantation site, indicating that the bio-degradable scaffold was fully resorbed. | 2008 | [132] |
| Adipose tissue derived stem cells (ASCs) |  | Nanofiber polycaprolactone scaffold | Sheep | Knee joints | defects treated with ASCs-scaffold constructs were completely filled with cartilage-like tissue, while other groups revealed the formation of a thin layer of cartilage-like tissue in the defects. the increase in collagen type 2 mRNA levels, aggrecan and Sox9 in ASCs/PCL groups in comparison with the other groups.the protein expression of collagen type 2 and formation of round and polygonal clusters of chondrocytes in ASCS/PCL group.According to our results nanofiber polycaprolactone promoted the chondrogenesis of infrapatellar adipose tissue derived stem cells in vivo and could offer significant promise in the biological functionality of stem cell tissue engineering in clinical practice.This study confirmed positive markers found in CD44 (97.48%),CD90 (97.20%), while few cells expressed hematopoietic markers such as CD45 (4.71%), or the endothelial marker CD31 (.43%), which were confirmed to be negative markers. | 2019 | [133] |
| Bone marrow-derived mesenchymal stem cells (MSCs) / meniscal cells |  | Collagen-hyaluronan scaffolds | New Zealand White rabbits | Knee joints | The regenerative potential of the meniscus by an autologous cell-based tissue engineering approach was shown even in a challenging setting of early osteoarthritis. Autologous MSCs and meniscal cells were found to have improved meniscal healing in an animal model, thus demonstrating their feasibility in a clinical setting.meniscal punch defects treated with MSCs showed partial defect filling with incomplete tissue differentiation of the repair tissue after 6 weeks following repair. After 3 months of treatment, meniscal defects were completely filled with dense repair tissue with stable integration to the native meniscus. The scoring results for both groups were high, particularly after 3 months, indicating good meniscus regeneration with differentiated tissue. Scoring values between 12 and 16 points were observed. | 2017 | [134] |
| Bone marrow stem cells (bMSCs) in the chondral phase |  | A novel type I collagen (Col)/glycosaminoglycan (GAGs)-porous titanium biphasic scaffold (CGT) | Beagles | Knee joints | The defect areas were examined grossly, histologically and by micro-CT. The most satisfied cartilage repairing result was in the CGTM group, while CGT alone was better than the control group. Abundant subchondral bone formation was observed in the CGTM and CGT groups but not the control group. Our findings demonstrate that a composite based on a novel biphasic scaffold combined with bMSCs shows a high potential to repair large osteochondral defects in a canine model.In the CGT and control groups, fibrous tissue was observed on the surface areas. However, there was continuous subchondral bone in the CGT group but not in the control group.Large quantities of new bone were formed in the porous titanium in both the CGTM and CGT groups.The bone defects in the control group were filled with fibrous tissue, and there was no new bone formation. New formed bone was also seen between the titanium and repaired cartilage tissue and the border between regenerated cartilage and subchondral bone was clear in the CGTM and CGT groups. | 2013 | [135] |
| Mesenchymal Stem Cells |  | Polyurethane Meniscal Scaffolds | Human | Knee joints | In tibial t2 mapping, values for the MPS group increased slightly at 9 months but returned to initial values at 12 months. in the aPS group, a clear decrease from 3 months to 12 months was observed. this difference tended to be significantly lower in the aPS group compared with the MPS group at the final time point. in the femur, a slight increase in the MPS group (47.8 ± 3.4) compared with the aPS group (45.3 ± 4.9) was observed.Meniscal substitution with polyurethane scaffold maintains normal t2 mapping values in adjacent cartilage at 12 months. the addition of MSC did not show any advantage in the protection of articular cartilage over acellular scaffolds. | 2019 | [136] |
| Autologous chondrocytes |  | Hyaluronan (Hyalograft-C) | Human | Knee joints | Biodegradable scaffolds seeded with autologous chondrocytes can be a viable treatment for chondral lesions. The type of tissue repair achieved demonstrated histologic characteristics similar to normal articular cartilage.Objective preoperative data improved from 6/32 (18.8%) with International Knee Documentation Committee A or B to 29/32 (90.7%) at 24 months after transplantation. Mean subjective scores improved from 43.2 points preoperatively to 73.6 points 24 months after implantation. Magnetic resonance imaging studies at 24 months revealed 71% to have an almost normal cartilage with positive correlation to clinical outcomes. Second-look arthroscopies in 6 cases revealed the repaired surface to be nearly normal with biopsy samples characterized as hyaline-like in appearance. | 2006 | [137] |
| Adipose-derived mesenchymal stem cells |  | Polycaprolactone/silk fibroin/gelatin/ascorbic acid (PCL/SF/Gel/AA) | New Zealand White rabbits | Knee joints | The PCl/SF/gel/aa scaffolds exhibited suitable mechanical properties,also showed good biocompatibility and proliferation for chondrocytes.Furthermore, vitamin C rendered them the highest antioxidant capacity. the PCl/SF/gel/aa scaffolds seeded with allogeneic aSCs was engrafted in New Zealand rabbits who underwent unilateral punch defect in the medial meniscus of the right knee. after 2 months postimplantation, macroscopic and histologic studies for new meniscus cartilage were performed.Our results indicated that the PCl/SF/gel/aa composite scaffolds seeded with allogeneic aSCs could successfully improve meniscus healing in damaged rabbits. | 2021 | [138] |

**References**

[1] V. Bravatà, L. Minafra, G.I. Forte, F.P. Cammarata, M. Saporito, F. Boniforti, D. Lio, M.C. Gilardi, C. Messa, DVWA gene polymorphisms and osteoarthritis, BMC research notes 8 (2015) 30.

[2] Q. Ji, Y. Zheng, G. Zhang, Y. Hu, X. Fan, Y. Hou, L. Wen, L. Li, Y. Xu, Y. Wang, F. Tang, Single-cell RNA-seq analysis reveals the progression of human osteoarthritis, Annals of the rheumatic diseases 78(1) (2019) 100-110.

[3] X. Ren, B. Hu, M. Song, Z. Ding, Y. Dang, Z. Liu, W. Zhang, Q. Ji, R. Ren, J. Ding, Maintenance of nucleolar homeostasis by CBX4 alleviates senescence and osteoarthritis, Cell reports 26(13) (2019) 3643-3656.

[4] X. Du, Y. Chen, Q. Zhang, J. Lin, Y. Yu, Z. Pan, H. Sun, C. Yuan, D. Yu, H. Wu, Ezh2 ameliorates osteoarthritis by activating TNFSF13B, Journal of Bone and Mineral Research 35(5) (2020) 956-965.

[5] X. Li, Z. Liao, Z. Deng, N. Chen, L. Zhao, Combining bulk and single-cell RNA-sequencing data to reveal gene expression pattern of chondrocytes in the osteoarthritic knee, Bioengineered 12(1) (2021) 997-1007.

[6] K. Kania, F. Colella, A.H.K. Riemen, H. Wang, K.A. Howard, T. Aigner, F. Dell’Accio, T.D. Capellini, A.J. Roelofs, C. De Bari, Regulation of Gdf5 expression in joint remodelling, repair and osteoarthritis, Scientific reports 10(1) (2020) 157.

[7] H. Kizawa, I. Kou, A. Iida, A. Sudo, Y. Miyamoto, A. Fukuda, A. Mabuchi, A. Kotani, A. Kawakami, S. Yamamoto, An aspartic acid repeat polymorphism in asporin inhibits chondrogenesis and increases susceptibility to osteoarthritis, Nature genetics 37(2) (2005) 138-144.

[8] U. Styrkarsdottir, S.H. Lund, G. Thorleifsson, F. Zink, O.A. Stefansson, J.K. Sigurdsson, K. Juliusson, K. Bjarnadottir, S. Sigurbjornsdottir, S. Jonsson, Meta-analysis of Icelandic and UK data sets identifies missense variants in SMO, IL11, COL11A1 and 13 more new loci associated with osteoarthritis, Nature genetics 50(12) (2018) 1681-1687.

[9] C.G. Boer, K. Hatzikotoulas, L. Southam, L. Stefánsdóttir, Y. Zhang, R.C. de Almeida, T.T. Wu, J. Zheng, A. Hartley, M. Teder-Laving, Deciphering osteoarthritis genetics across 826,690 individuals from 9 populations, Cell 184(18) (2021) 4784-4818.

[10] C.G. Boer, M.S. Yau, S.J. Rice, R.C. de Almeida, K. Cheung, U. Styrkarsdottir, L. Southam, L. Broer, J.M. Wilkinson, A.G. Uitterlinden, Genome-wide association of phenotypes based on clustering patterns of hand osteoarthritis identify WNT9A as novel osteoarthritis gene, Annals of the rheumatic diseases 80(3) (2021) 367-375.

[11] J. Lin, G. Wu, Z. Zhao, Y. Huang, J. Chen, C. Fu, J. Ye, X. Liu, Bioinformatics analysis to identify key genes and pathways influencing synovial inflammation in osteoarthritis, Molecular medicine reports 18(6) (2018) 5594-5602.

[12] A. Ratneswaran, M. Kapoor, Osteoarthritis year in review: genetics, genomics, epigenetics, Osteoarthritis and cartilage 29(2) (2021) 151-160.

[13] C.H. Ma, Q. Lv, Y. Cao, Q. Wang, X.K. Zhou, B.W. Ye, C.Q. Yi, Genes relevant with osteoarthritis by comparison gene expression profiles of synovial membrane of osteoarthritis patients at different stages, European Review for Medical & Pharmacological Sciences 18(3) (2014).

[14] T. Ayanoglu, H. Atalar, E. Esen, M.B. Ataoğlu, S. Turanlı, K. Demircan, The role of ADAMTS genes in the end stage of hip osteoarthritis, Acta orthopaedica et traumatologica turcica 53(2) (2019) 140-144.

[15] Z. Zhang, Y. Mei, M. Feng, C. Wang, P. Yang, R. Tian, The relationship between common variants in the DPEP1 gene and the susceptibility and clinical severity of osteoarthritis, International journal of rheumatic diseases 24(9) (2021) 1192-1199.

[16] A.R. Upton, C.A. Holding, A.A. Dharmapatni, D.R. Haynes, The expression of RANKL and OPG in the various grades of osteoarthritic cartilage, Rheumatology international 32 (2012) 535-540.

[17] L. Zhong, X. Huang, M. Karperien, J.N. Post, Correlation between gene expression and osteoarthritis progression in human, International journal of molecular sciences 17(7) (2016) 1126.

[18] R. Zhang, H. Fang, Y. Chen, J. Shen, H. Lu, C. Zeng, J. Ren, H. Zeng, Z. Li, S. Chen, Gene expression analyses of subchondral bone in early experimental osteoarthritis by microarray, PloS one 7(2) (2012) e32356.

[19] C. Li, J. Luo, X. Xu, Z. Zhou, S. Ying, X. Liao, K. Wu, Single cell sequencing revealed the underlying pathogenesis of the development of osteoarthritis, Gene 757 (2020) 144939.

[20] S. Wang, H. Wang, W. Liu, B. Wei, Identification of key genes and pathways associated with sex differences in osteoarthritis based on bioinformatics analysis, BioMed research international 2019 (1)(2019)3482751.

[21] C. Shepherd, A.J. Skelton, M.D. Rushton, L.N. Reynard, J. Loughlin, Expression analysis of the osteoarthritis genetic susceptibility locus mapping to an intron of the MCF2L gene and marked by the polymorphism rs11842874, BMC medical genetics 16(1) (2015) 1-11.

[22] Z. Zhu, L. Zhong, R. Li, Y. Liu, X. Chen, Z. Li, L. Huang, Study of osteoarthritis-related hub genes based on bioinformatics analysis, BioMed research international 2020 (2020).

[23] P. Krzeski, C. Buckland-Wright, G. Bálint, G.A. Cline, K. Stoner, R. Lyon, J. Beary, W.S. Aronstein, T.D. Spector, Development of musculoskeletal toxicity without clear benefit after administration of PG-116800, a matrix metalloproteinase inhibitor, to patients with knee osteoarthritis: a randomized, 12-month, double-blind, placebo-controlled study, Arthritis research & therapy 9(5) (2007) 1-11.

[24] J.-Y. Reginster, N. Veronese, Highly purified chondroitin sulfate: a literature review on clinical efficacy and pharmacoeconomic aspects in osteoarthritis treatment, Aging Clinical and Experimental Research 33 (2021) 37-47.

[25] A. Vaquero-Picado, E.C. Rodríguez-Merchán, Intra-articular injections of corticosteroids and hyaluronic acid in knee osteoarthritis, Comprehensive Treatment of Knee Osteoarthritis: Recent Advances (2020) 25-29.

[26] C. Zhang, Q. Liao, J.-H. Ming, G.-L. Hu, Q. Chen, S.-Q. Liu, Y.-M. Li, The effects of chitosan oligosaccharides on OPG and RANKL expression in a rat osteoarthritis model, Acta Cirurgica Brasileira 32 (2017) 418-428.

[27] Y. Murahashi, F. Yano, R. Chijimatsu, H. Nakamoto, Y. Maenohara, M. Amakawa, Y. Miyake, H. Yamanaka, K. Iba, T. Yamashita, Oral administration of EP4-selective agonist KAG-308 suppresses mouse knee osteoarthritis development through reduction of chondrocyte hypertrophy and TNF secretion, Scientific reports 9(1) (2019) 20329.

[28] S. Pagani, M. Minguzzi, L. Sicuro, F. Veronesi, S. Santi, A. Scotto D’Abusco, M. Fini, R.M. Borzì, The N-acetyl phenylalanine glucosamine derivative attenuates the inflammatory/catabolic environment in a chondrocyte-synoviocyte co-culture system, Scientific reports 9(1) (2019) 13603.

[29] J.N. Campbell, R. Stevens, P. Hanson, J. Connolly, D.S. Meske, M.-K. Chung, B.D.X. Lascelles, Injectable capsaicin for the management of pain due to osteoarthritis, Molecules 26(4) (2021) 778.

[30] Y. Wei, L. Yan, L. Luo, T. Gui, B. Jang, A. Amirshaghaghi, T. You, A. Tsourkas, L. Qin, Z. Cheng, Phospholipase A2 inhibitor–loaded micellar nanoparticles attenuate inflammation and mitigate osteoarthritis progression, Science advances 7(15) (2021) eabe6374.

[31] T.K. Kvien, E. Fjeld, B. Slatkowsky-Christensen, M. Nichols, Y. Zhang, A. Prøven, K. Mikkelsen, Ø. Palm, A.A. Borisy, J. Lessem, Efficacy and safety of a novel synergistic drug candidate, CRx-102, in hand osteoarthritis, Annals of the rheumatic diseases 67(7) (2008) 942-948.

[32] M. Banning, Topical diclofenac: clinical effectiveness and current uses in osteoarthritis of the knee and soft tissue injuries, Expert opinion on pharmacotherapy 9(16) (2008) 2921-2929.

[33] J. Liu, A.R. Shikhman, M.K. Lotz, C.-H. Wong, Hexosaminidase inhibitors as new drug candidates for the therapy of osteoarthritis, Chemistry & biology 8(7) (2001) 701-711.

[34] J.-N. Gouze, E. Gouze, M.P. Popp, M.L. Bush, E.A. Dacanay, J.D. Kay, P.P. Levings, K.R. Patel, J.-P.S. Saran, R.S. Watson, Exogenous glucosamine globally protects chondrocytes from the arthritogenic effects of IL-1β, Arthritis research & therapy 8(6) (2006) 1-14.

[35] S. Yang, others. Effects of glucosamine and chondroitin on treating knee osteoarthritis: An analysis with marginal structural models, Arthritis & Rheumatism 22 (2014) S203.

[36] G. Han, G. Wang, X. Zhu, H. Shao, F. Liu, P. Yang, Y. Ying, F. Wang, P. Ling, Preparation of xanthan gum injection and its protective effect on articular cartilage in the development of osteoarthritis, Carbohydrate Polymers 87(2) (2012) 1837-1842.

[37] G. Han, Q. Chen, F. Liu, Z. Cui, H. Shao, F. Liu, A. Ma, J. Liao, B. Guo, Y. Guo, Low molecular weight xanthan gum for treating osteoarthritis, Carbohydrate polymers 164 (2017) 386-395.

[38] Y. Sun, A.M. Franklin, D.R. Mauerhan, E.N. Hanley, Biological effects of phosphocitrate on osteoarthritic articular chondrocytes, The Open Rheumatology Journal 11 (2017) 62.

[39] T.A. Rodrigues, A.O. Freire, B.F. Bonfim, M.S.S. Cartágenes, J.B.S. Garcia, Strontium ranelate as a possible disease-modifying osteoarthritis drug: a systematic review, Brazilian Journal of Medical and Biological Research 51 (2018).

[40] D. Agas, F. Laus, G. Lacava, A. Marchegiani, S. Deng, F. Magnoni, G.G. Silva, P. Di Martino, M.G. Sabbieti, R. Censi, Thermosensitive hybrid hyaluronan/p (HPMAm‐lac)‐PEG hydrogels enhance cartilage regeneration in a mouse model of osteoarthritis, Journal of cellular physiology 234(11) (2019) 20013-20027.

[41] G.-Y. Min, J.-M. Park, I.-H. Joo, D.-H. Kim, Inhibition effect of Caragana sinica root extracts on Osteoarthritis through MAPKs, NF-κB signaling pathway, International Journal of Medical Sciences 18(4) (2021) 861.

[42] N. Shivnath, S. Siddiqui, V. Rawat, M.S. Khan, M. Arshad, Solanum xanthocarpum fruit extract promotes chondrocyte proliferation in vitro and protects cartilage damage in collagenase induced osteoarthritic rats (article reference number: JEP 114028), Journal of Ethnopharmacology 274 (2021) 114028.

[43] N. Fujitsuka, M. Tamai, K. Tsuchiya, S. Iizuka, N. Tsuchiya, B. Makino, Boiogito a kampo medicine improves hydrarthrosis in a rat model of knee osteoarthritis (2015) BMC Complement Altern, 15(2015)1-8.

[44] Q. Liu, Z. Wu, D. Hu, L. Zhang, L. Wang, G. Liu, Low dose of indomethacin and Hedgehog signaling inhibitor administration synergistically attenuates cartilage damage in osteoarthritis by controlling chondrocytes pyroptosis, Gene 712 (2019) 143959.

[45] J.M. Alvaro-Gracia, Licofelone—clinical update on a novel LOX/COX inhibitor for the treatment of osteoarthritis, Rheumatology 43(suppl_1) (2004) i21-i25.

[46] H.S. Na, J.Y. Kwon, S.-Y. Lee, S.H. Lee, A.R. Lee, J.S. Woo, K. Jung, K.-H. Cho, J.-W. Choi, D.H. Lee, Metformin attenuates monosodium-iodoacetate-induced osteoarthritis via regulation of pain mediators and the autophagy–lysosomal pathway, Cells 10(3) (2021) 681.

[47] T. Ma, L. Lv, Y. Yu, L. Jia, X. Song, X. Xu, T. Li, X. Sheng, H. Wang, J. Zhang, Bilobalide exerts anti-inflammatory effects on chondrocytes through the AMPK/SIRT1/mTOR pathway to attenuate ACLT-induced post-traumatic osteoarthritis in rats, Frontiers in pharmacology 13 (2022) 783506.

[48] L. Yang, X. Zhao, J. Zhang, S. Ma, L. Jiang, Q. Wei, M. Cai, F. Zhou, Synthesis of charged chitosan nanoparticles as functional biolubricant, Colloids and Surfaces B: Biointerfaces 206 (2021) 111973.

[49] F. Yano, H. Hojo, S. Ohba, A. Fukai, Y. Hosaka, T. Ikeda, T. Saito, M. Hirata, H. Chikuda, T. Takato, A novel disease-modifying osteoarthritis drug candidate targeting Runx1, Annals of the rheumatic diseases 72(5) (2013) 748-753.

[50] Y. Min, D. Kim, G.G.D. Suminda, X. Zhao, M. Kim, Y. Zhao, Y.-O. Son, GSK5182, 4-hydroxytamoxifen analog, a new potential therapeutic drug for osteoarthritis, Pharmaceuticals 13(12) (2020) 429.

[51] H. Guo, W. Yin, Z. Zou, C. Zhang, M. Sun, L. Min, L. Yang, L. Kong, Quercitrin alleviates cartilage extracellular matrix degradation and delays ACLT rat osteoarthritis development: An in vivo and in vitro study, Journal of advanced research 28 (2021) 255-267.

[52] H. Wang, Z. Jiang, Z. Pang, G. Qi, B. Hua, Z. Yan, H. Yuan, Engeletin protects against TNF-α-induced apoptosis and reactive oxygen species generation in chondrocytes and alleviates osteoarthritis in vivo, Journal of Inflammation Research 14 (2021) 745.

[53] M.-C. Choi, W.H. Choi, Mithramycin A alleviates osteoarthritic cartilage destruction by inhibiting HIF-2α expression, International journal of molecular sciences 19(5) (2018) 1411.

[54] T. Izawa, I.R. Hutami, E. Tanaka, Potential role of rebamipide in osteoclast differentiation and mandibular condylar cartilage homeostasis, Current rheumatology reviews 14(1) (2018) 62-69.

[55] A. Haseeb, M.Y. Ansari, T.M. Haqqi, Harpagoside suppresses IL‐6 expression in primary human osteoarthritis chondrocytes, Journal of Orthopaedic Research 35(2) (2017) 311-320.

[56] L. Ma, X. Zhao, Y. Liu, J. Wu, X. Yang, Q. Jin, Dihydroartemisinin attenuates osteoarthritis by inhibiting abnormal bone remodeling and angiogenesis in subchondral bone, International Journal of Molecular Medicine 47(3) (2021) 1-1.

[57] J.M. Chun, A.Y. Lee, J.Y. Nam, M.Y. Lee, M.S. Choe, K.S. Lim, C. Kim, J.-S. Kim, Protective effects of Phlomis umbrosa extract on a monosodium iodoacetate–induced osteoarthritis model and prediction of molecular mechanisms using transcriptomics, Phytomedicine 81 (2021) 153429.

[58] B. Bai, Y. Li, Danshen prevents articular cartilage degeneration via antioxidation in rabbits with osteoarthritis, Osteoarthritis and Cartilage 24(3) (2016) 514-520.

[59] W. Fan, J. Li, L. Yuan, J. Chen, Z. Wang, Y. Wang, C. Guo, X. Mo, Z. Yan, Intra-articular injection of kartogenin-conjugated polyurethane nanoparticles attenuates the progression of osteoarthritis, Drug delivery 25(1) (2018) 1004-1012.

[60] H.B. Si, Y. Zeng, S.Y. Liu, Z.K. Zhou, Y.N. Chen, J.Q. Cheng, Y.R. Lu, B. Shen, Intra-articular injection of microRNA-140 (miRNA-140) alleviates osteoarthritis (OA) progression by modulating extracellular matrix (ECM) homeostasis in rats, Osteoarthritis and cartilage 25(10) (2017) 1698-1707.

[61] H. Yu, S. Yao, C. Zhou, F. Fu, H. Luo, W. Du, H. Jin, P. Tong, D. Chen, C. Wu, Morroniside attenuates apoptosis and pyroptosis of chondrocytes and ameliorates osteoarthritic development by inhibiting NF-κB signaling, Journal of Ethnopharmacology 266 (2021) 113447.

[62] P. Li, H. Li, X. Shu, M. Wu, J. Liu, T. Hao, H. Cui, L. Zheng, Intra-articular delivery of flurbiprofen sustained release thermogel: improved therapeutic outcome of collagenase II-induced rat knee osteoarthritis, Drug delivery 27(1) (2020) 1034-1043.

[63] C. Kim, O.H. Jeon, D.H. Kim, J.J. Chae, L. Shores, N. Bernstein, R. Bhattacharya, J.M. Coburn, K.J. Yarema, J.H. Elisseeff, Local delivery of a carbohydrate analog for reducing arthritic inflammation and rebuilding cartilage, Biomaterials 83 (2016) 93-101.

[64] W. Zheng, C. Chen, C. Zhang, L. Cai, H. Chen, The protective effect of phloretin in osteoarthritis: an in vitro and in vivo study, Food & function 9(1) (2018) 263-278.

[65] H.T. Philpott, M. O'Brien, J.J. McDougall, Attenuation of early phase inflammation by cannabidiol prevents pain and nerve damage in rat osteoarthritis, Pain 158(12) (2017) 2442.

[66] A. Moretti, M. Paoletta, S. Liguori, W. Ilardi, F. Snichelotto, G. Toro, F. Gimigliano, G. Iolascon, The rationale for the intra-articular administration of clodronate in osteoarthritis, International journal of molecular sciences 22(5) (2021) 2693.

[67] Z.H. Wan, Q. Zhao, Gypenoside inhibits interleukin‐1β‐induced inflammatory response in human osteoarthritis chondrocytes, Journal of biochemical and molecular toxicology 31(9) (2017) e21926.

[68] I.-C. Young, S.-T. Chuang, C.-H. Hsu, Y.-J. Sun, F.-H. Lin, C-phycocyanin alleviates osteoarthritic injury in chondrocytes stimulated with H2O2 and compressive stress, International journal of biological macromolecules 93 (2016) 852-859.

[69] H. Endisha, MicroRNA-34a-5p Promotes Joint Destruction During Obesity and Osteoarthritis, University of Toronto (Canada)2019.

[70] Y.-p. Zhao, B. Liu, Q.-y. Tian, J.-l. Wei, B. Richbourgh, C.-j. Liu, Progranulin protects against osteoarthritis through interacting with TNF-α and β-Catenin signalling, Annals of the rheumatic diseases 74(12) (2015) 2244-2253.

[71] J. Ouyang, H. Jiang, H. Fang, W. Cui, D. Cai, Isoimperatorin ameliorates osteoarthritis by downregulating the mammalian target of rapamycin C1 signaling pathway, Molecular medicine reports 16(6) (2017) 9636-9644.

[72] W. Lu, L. Wang, C. Wo, J. Yao, Ketamine attenuates osteoarthritis of the knee via modulation of inflammatory responses in a rabbit model, Molecular medicine reports 13(6) (2016) 5013-5020.

[73] Y. Sun, W. Liu, H. Zhang, H. Li, J. Liu, F. Zhang, T. Jiang, S. Jiang, Curcumin prevents osteoarthritis by inhibiting the activation of inflammasome NLRP3, Journal of Interferon & Cytokine Research 37(10) (2017) 449-455.

[74] D.-H. Chen, G. Zheng, X.-Y. Zhong, Z.-H. Lin, S.-W. Yang, H.-X. Liu, P. Shang, Oroxylin A attenuates osteoarthritis progression by dual inhibition of cell inflammation and hypertrophy, Food & function 12(1) (2021) 328-339.

[75] J. Chahal, A. Gómez-Aristizábal, K. Shestopaloff, S. Bhatt, A. Chaboureau, A. Fazio, J. Chisholm, A. Weston, J. Chiovitti, A. Keating, Bone marrow mesenchymal stromal cell treatment in patients with osteoarthritis results in overall improvement in pain and symptoms and reduces synovial inflammation, Stem cells translational medicine 8(8) (2019) 746-757.

[76] R. Bastos, M. Mathias, R. Andrade, R.J.F.C. Amaral, V. Schott, A. Balduino, R. Bastos, J. Miguel Oliveira, R.L. Reis, S. Rodeo, Intra-articular injection of culture-expanded mesenchymal stem cells with or without addition of platelet-rich plasma is effective in decreasing pain and symptoms in knee osteoarthritis: a controlled, double-blind clinical trial, Knee Surgery, Sports Traumatology, Arthroscopy 28 (2020) 1989-1999.

[77] M. Gutierres, M.A. Lopes, N.S. Hussain, A.F. Lemos, J.M.F. Ferreira, A. Afonso, A.T. Cabral, L. Almeida, J.D. Santos, Bone ingrowth in macroporous Bonelike® for orthopaedic applications, Acta Biomaterialia 4(2) (2008) 370-377.

[78] Y. Liu, G. Dzidotor, T.T. Le, T. Vinikoor, K. Morgan, E.J. Curry, R. Das, A. McClinton, E. Eisenberg, L.N. Apuzzo, Exercise-induced piezoelectric stimulation for cartilage regeneration in rabbits, Science Translational Medicine 14(627) (2022) eabi7282.

[79] A. Bhattacharjee, D.S. Katti, Sulfated carboxymethylcellulose-based scaffold mediated delivery of Timp3 alleviates osteoarthritis, International Journal of Biological Macromolecules 212 (2022) 54-66.

[80] G. Xu, Y. Zhao, Y. Geng, S. Cao, P. Pan, J. Wang, J. Chen, Nano-hybrid gradient scaffold for articular repair, Colloids and Surfaces B: Biointerfaces 208 (2021) 112116.

[81] J. Zhu, S. Yang, Y. Qi, Z. Gong, H. Zhang, K. Liang, P. Shen, Y.-Y. Huang, Z. Zhang, W. Ye, Stem cell–homing hydrogel-based miR-29b-5p delivery promotes cartilage regeneration by suppressing senescence in an osteoarthritis rat model, Science Advances 8(13) (2022) eabk0011.

[82] S. Li, J. Liu, S. Liu, W. Jiao, X. Wang, Chitosan oligosaccharides packaged into rat adipose mesenchymal stem cells-derived extracellular vesicles facilitating cartilage injury repair and alleviating osteoarthritis, Journal of nanobiotechnology 19 (2021) 1-19.

[83] E.E. Moore, A.M. Bendele, D.L. Thompson, A. Littau, K.S. Waggie, B. Reardon, J.L. Ellsworth, Fibroblast growth factor-18 stimulates chondrogenesis and cartilage repair in a rat model of injury-induced osteoarthritis, Osteoarthritis and cartilage 13(7) (2005) 623-631.

[84] T.A. Holland, E.W.H. Bodde, V. Cuijpers, L.S. Baggett, Y. Tabata, A.G. Mikos, J.A. Jansen, Degradable hydrogel scaffolds for in vivo delivery of single and dual growth factors in cartilage repair, Osteoarthritis and Cartilage 15(2) (2007) 187-197.

[85] P. Mainil-Varlet, F. Rieser, S. Grogan, W. Mueller, C. Saager, R.P. Jakob, Articular cartilage repair using a tissue-engineered cartilage-like implant: an animal study, Osteoarthritis and cartilage 9 (2001) S6-S15.

[86] M. Castilho, V. Mouser, M. Chen, J. Malda, K. Ito, Bi-layered micro-fibre reinforced hydrogels for articular cartilage regeneration, Acta biomaterialia 95 (2019) 297-306.

[87] L. Xu, A. Urita, T. Onodera, R. Hishimura, T. Nonoyama, M. Hamasaki, D. Liang, K. Homan, J.P. Gong, N. Iwasaki, Ultrapurified alginate gel containing bone marrow aspirate concentrate enhances cartilage and bone regeneration on osteochondral defects in a rabbit model, The American Journal of Sports Medicine 49(8) (2021) 2199-2210.

[88] A. Shokri, K. Ramezani, M.R. Jamalpour, C. Mohammadi, F. Vahdatinia, A.D. Irani, E. Sharifi, R. Haddadi, S. Jamshidi, L.M. Amirabad, In vivo efficacy of 3D‐printed elastin–gelatin–hyaluronic acid scaffolds for regeneration of nasal septal cartilage defects, Journal of Biomedical Materials Research Part B: Applied Biomaterials 110(3) (2022) 614-624.

[89] W. Changchen, W. Hongquan, Z. Bo, X. Leilei, J. Haiyue, P. Bo, The characterization, cytotoxicity, macrophage response and tissue regeneration of decellularized cartilage in costal cartilage defects, Acta Biomaterialia 136 (2021) 147-158.

[90] P. Das, R. Mishra, B. Devi, K. Rajesh, P. Basak, M. Roy, P. Roy, D. Lahiri, S.K. Nandi, Decellularized xenogenic cartilage extracellular matrix (ECM) scaffolds for the reconstruction of osteochondral defects in rabbits, Journal of Materials Chemistry B 9(24) (2021) 4873-4894.

[91] Y. Huang, H. Fan, X. Gong, L. Yang, F. Wang, Scaffold with natural calcified cartilage zone for osteochondral defect repair in minipigs, The American Journal of Sports Medicine 49(7) (2021) 1883-1891.

[92] T.A. Hassan, M.A. Maher, A.F. El Karmoty, Z.S.O. Ahmed, M.A. Ibrahim, H. Rizk, A.T. Reyad, Auricular cartilage regeneration using different types of mesenchymal stem cells in rabbits, Biological Research 55(1) (2022) 40.

[93] T. He, B. Li, T. Colombani, K. Joshi-Navare, S. Mehta, J. Kisiday, S.A. Bencherif, A.G. Bajpayee, Hyaluronic acid-based shape-memory cryogel scaffolds for focal cartilage defect repair, Tissue Engineering Part A 27(11-12) (2021) 748-760.

[94] R.K. Siu, J.N. Zara, Y. Hou, A.W. James, J. Kwak, X. Zhang, K. Ting, B.M. Wu, C. Soo, M. Lee, NELL-1 promotes cartilage regeneration in an in vivo rabbit model, Tissue Engineering Part A 18(3-4) (2012) 252-261.

[95] C.C. Wu, S.Y. Sheu, L.H. Hsu, K.C. Yang, C.C. Tseng, T.F. Kuo, Intra‐articular Injection of platelet‐rich fibrin releasates in combination with bone marrow‐derived mesenchymal stem cells in the treatment of articular cartilage defects: An in vivo study in rabbits, Journal of Biomedical Materials Research Part B: Applied Biomaterials 105(6) (2017) 1536-1543.

[96] R.A.V. Bolaños, S.M. Cokelaere, J.M.E. McDermott, K.E.M. Benders, U. Gbureck, S.G.M. Plomp, H. Weinans, J. Groll, P.R. Van Weeren, J. Malda, The use of a cartilage decellularized matrix scaffold for the repair of osteochondral defects: the importance of long-term studies in a large animal model, Osteoarthritis and cartilage 25(3) (2017) 413-420.

[97] H. Kang, J. Peng, S. Lu, S. Liu, L. Zhang, J. Huang, X. Sui, B. Zhao, A. Wang, W. Xu, In vivo cartilage repair using adipose‐derived stem cell‐loaded decellularized cartilage ECM scaffolds, Journal of tissue engineering and regenerative medicine 8(6) (2014) 442-453.

[98] H. Wang, T. Peng, H. Wu, J. Chen, M. Chen, L. Mei, F. Li, W. Wang, C. Wu, X. Pan, In situ biomimetic lyotropic liquid crystal gel for full-thickness cartilage defect regeneration, Journal of Controlled Release 338 (2021) 623-632.

[99] N.J. Chang, C.F. Lam, C.C. Lin, W.L. Chen, C.F. Li, Y.T. Lin, M.L. Yeh, Transplantation of autologous endothelial progenitor cells in porous PLGA scaffolds create a microenvironment for the regeneration of hyaline cartilage in rabbits, Osteoarthritis and cartilage 21(10) (2013) 1613-1622.

[100] Y.S. Kim, Y.J. Choi, D.S. Suh, D.B. Heo, Y.I. Kim, J.-S. Ryu, Y.G. Koh, Mesenchymal stem cell implantation in osteoarthritic knees: is fibrin glue effective as a scaffold?, The American journal of sports medicine 43(1) (2015) 176-185.

[101] K. Shimomura, Y. Yasui, K. Koizumi, R. Chijimatsu, D.A. Hart, Y. Yonetani, W. Ando, T. Nishii, T. Kanamoto, S. Horibe, First-in-human pilot study of implantation of a scaffold-free tissue-engineered construct generated from autologous synovial mesenchymal stem cells for repair of knee chondral lesions, The American journal of sports medicine 46(10) (2018) 2384-2393.

[102] A.P. Hollander, S.C. Dickinson, T.J. Sims, P. Brun, R. Cortivo, E. Kon, M. Marcacci, S. Zanasi, A. Borrione, C.D. Luca, Maturation of tissue engineered cartilage implanted in injured and osteoarthritic human knees, Tissue engineering 12(7) (2006) 1787-1798.

[103] P. Haghighi, A. Shamloo, Fabrication of a novel 3D scaffold for cartilage tissue repair: In-vitro and in-vivo study, Materials Science and Engineering: C 128 (2021) 112285.

[104] A.M. Haleem, A.A.E. Singergy, D. Sabry, H.M. Atta, L.A. Rashed, C.R. Chu, M.T.E. Shewy, A. Azzam, M.T.A. Aziz, The clinical use of human culture–expanded autologous bone marrow mesenchymal stem cells transplanted on platelet-rich fibrin glue in the treatment of articular cartilage defects: a pilot study and preliminary results, Cartilage 1(4) (2010) 253-261.

[105] W. Shi, M. Sun, X. Hu, B. Ren, J. Cheng, C. Li, X. Duan, X. Fu, J. Zhang, H. Chen, Structurally and functionally optimized silk‐fibroin–gelatin scaffold using 3D printing to repair cartilage injury in vitro and in vivo, Advanced materials 29(29) (2017) 1701089.

[106] B. Grigolo, G. Lisignoli, G. Desando, C. Cavallo, E. Marconi, M. Tschon, G. Giavaresi, M. Fini, R. Giardino, A. Facchini, Osteoarthritis treated with mesenchymal stem cells on hyaluronan-based scaffold in rabbit, Tissue Engineering Part C: Methods 15(4) (2009) 647-658.

[107] J. Deng, R. She, W. Huang, Z. Dong, G. Mo, B. Liu, A silk fibroin/chitosan scaffold in combination with bone marrow-derived mesenchymal stem cells to repair cartilage defects in the rabbit knee, Journal of Materials Science: Materials in Medicine 24 (2013) 2037-2046.

[108] J. Xie, Z. Han, M. Naito, A. Maeyama, S.H. Kim, Y.H. Kim, T. Matsuda, Articular cartilage tissue engineering based on a mechano‐active scaffold made of poly (l‐lactide‐co‐ε‐caprolactone): In vivo performance in adult rabbits, Journal of Biomedical Materials Research Part B: Applied Biomaterials 94(1) (2010) 80-88.

[109] L. Cui, Y. Wu, L. Cen, H. Zhou, S. Yin, G. Liu, W. Liu, Y. Cao, Repair of articular cartilage defect in non-weight bearing areas using adipose derived stem cells loaded polyglycolic acid mesh, Biomaterials 30(14) (2009) 2683-2693.

[110] W. Zhang, J. Chen, J. Tao, Y. Jiang, C. Hu, L. Huang, J. Ji, H.W. Ouyang, The use of type 1 collagen scaffold containing stromal cell-derived factor-1 to create a matrix environment conducive to partial-thickness cartilage defects repair, Biomaterials 34(3) (2013) 713-723.

[111] A. Sukegawa, N. Iwasaki, Y. Kasahara, T. Onodera, T. Igarashi, A. Minami, Repair of rabbit osteochondral defects by an acellular technique with an ultrapurified alginate gel containing stromal cell-derived factor-1, Tissue Engineering Part A 18(9-10) (2012) 934-945.

[112] X. Guo, C. Wang, Y. Zhang, R. Xia, M. Hu, C. Duan, Q. Zhao, L. Dong, J. Lu, Y. Qing Song, Repair of large articular cartilage defects with implants of autologous mesenchymal stem cells seeded into β-tricalcium phosphate in a sheep model, Tissue engineering 10(11-12) (2004) 1818-1829.

[113] W. Yu, B. Hu, K.O. Boakye-Yiadom, W. Ho, Q. Chen, X. Xu, X.-Q. Zhang, Injectable hydrogel mediated delivery of gene-engineered adipose-derived stem cells for enhanced osteoarthritis treatment, Biomaterials Science 9(22) (2021) 7603-7616.

[114] M. Bhattacharjee, J.L. Escobar Ivirico, H.-M. Kan, S. Shah, T. Otsuka, R. Bordett, M. Barajaa, N. Nagiah, R. Pandey, L.S. Nair, Injectable amnion hydrogel-mediated delivery of adipose-derived stem cells for osteoarthritis treatment, Proceedings of the National Academy of Sciences 119(4) (2022) e2120968119.

[115] L. Mei, B. Shen, J. Xue, S. Liu, A. Ma, F. Liu, H. Shao, J. Chen, Q. Chen, F. Liu, Adipose tissue–derived stem cells in combination with xanthan gum attenuate osteoarthritis progression in an experimental rat model, Biochemical and biophysical research communications 494(1-2) (2017) 285-291.

[116] B.B. Christensen, C.B. Foldager, O.M. Hansen, A.A. Kristiansen, D.Q.S. Le, A.D. Nielsen, J.V. Nygaard, C.E. Bünger, M. Lind, A novel nano-structured porous polycaprolactone scaffold improves hyaline cartilage repair in a rabbit model compared to a collagen type I/III scaffold: in vitro and in vivo studies, Knee Surgery, Sports Traumatology, Arthroscopy 20 (2012) 1192-1204.

[117] S. Been, J. Choi, H. Cho, G. Jeon, J.E. Song, A. Bucciarelli, G. Khang, Preparation and characterization of a soluble eggshell membrane/agarose composite scaffold with possible applications in cartilage regeneration, Journal of Tissue Engineering and Regenerative Medicine 15(4) (2021) 375-387.

[118] S. Tangyuenyong, P. Kongdang, N. Sirikaew, S. Ongchai, First study on the effect of transforming growth factor beta 1 and insulin-like growth factor 1 on the chondrogenesis of elephant articular chondrocytes in a scaffold-based 3D culture model, Veterinary World 15(7) (2022) 1869.

[119] J. Wang, Y. Wang, X. Sun, D. Liu, C. Huang, J. Wu, C. Yang, Q. Zhang, Biomimetic cartilage scaffold with orientated porous structure of two factors for cartilage repair of knee osteoarthritis, Artificial cells, nanomedicine, and biotechnology 47(1) (2019) 1710-1721.

[120] G. Desando, G. Giavaresi, C. Cavallo, I. Bartolotti, F. Sartoni, N. Nicoli Aldini, L. Martini, A. Parrilli, E. Mariani, M. Fini, Autologous bone marrow concentrate in a sheep model of osteoarthritis: new perspectives for cartilage and meniscus repair, Tissue Engineering Part C: Methods 22(6) (2016) 608-619.

[121] X. Lv, J. He, X. Zhang, X. Luo, N. He, Z. Sun, H. Xia, V. Liu, L. Zhang, X. Lin, Comparative efficacy of autologous stromal vascular fraction and autologous adipose-derived mesenchymal stem cells combined with hyaluronic acid for the treatment of sheep osteoarthritis, Cell transplantation 27(7) (2018) 1111-1125.

[122] Y. Zhang, Y. Han, Y. Peng, J. Lei, F. Chang, Bionic biphasic composite scaffolds with osteochondrogenic factors for regeneration of full-thickness osteochondral defects, Biomaterials Science 10(7) (2022) 1713-1723.

[123] P. Duan, Z. Pan, L. Cao, J. Gao, H. Yao, X. Liu, R. Guo, X. Liang, J. Dong, J. Ding, Restoration of osteochondral defects by implanting bilayered poly (lactide-co-glycolide) porous scaffolds in rabbit joints for 12 and 24 weeks, Journal of Orthopaedic Translation 19 (2019) 68-80.

[124] P. Neybecker, C. Henrionnet, E. Pape, D. Mainard, L. Galois, D. Loeuille, P. Gillet, A. Pinzano, In vitro and in vivo potentialities for cartilage repair from human advanced knee osteoarthritis synovial fluid-derived mesenchymal stem cells, Stem cell research & therapy 9 (2018) 1-15.

[125] Y. Hu, J. Ran, Z. Zheng, Z. Jin, X. Chen, Z. Yin, C. Tang, Y. Chen, J. Huang, H. Le, Exogenous stromal derived factor-1 releasing silk scaffold combined with intra-articular injection of progenitor cells promotes bone-ligament-bone regeneration, Acta biomaterialia 71 (2018) 168-183.

[126] S. Ravindran, M. Kotecha, C.-C. Huang, A. Ye, P. Pothirajan, Z. Yin, R. Magin, A. George, Biological and MRI characterization of biomimetic ECM scaffolds for cartilage tissue regeneration, Biomaterials 71 (2015) 58-70.

[127] Y.-H. Hsieh, M.-F. Hsieh, C.-H. Fang, C.-P. Jiang, B. Lin, H.-M. Lee, Osteochondral regeneration induced by TGF-β loaded photo cross-linked hyaluronic acid hydrogel infiltrated in fused deposition-manufactured composite scaffold of hydroxyapatite and poly (ethylene glycol)-block-poly (ε-caprolactone), Polymers 9(5) (2017) 182.

[128] A.R. Martin, J.M. Patel, R.C. Locke, M.R. Eby, K.S. Saleh, M.D. Davidson, M.L. Sennett, H.M. Zlotnick, A.H. Chang, J.L. Carey, Nanofibrous hyaluronic acid scaffolds delivering TGF-β3 and SDF-1α for articular cartilage repair in a large animal model, Acta biomaterialia 126 (2021) 170-182.

[129] K.A. Glass, J.M. Link, J.M. Brunger, F.T. Moutos, C.A. Gersbach, F. Guilak, Tissue-engineered cartilage with inducible and tunable immunomodulatory properties, Biomaterials 35(22) (2014) 5921-5931.

[130] E. Kon, G. Filardo, M. Tschon, M. Fini, G. Giavaresi, L.M. Reggiani, C. Chiari, S. Nehrer, I. Martin, D.M. Salter, Tissue engineering for total meniscal substitution: animal study in sheep model—results at 12 months, Tissue Engineering Part A 18(15-16) (2012) 1573-1582.

[131] Y. Nakagawa, L.A. Fortier, J.J. Mao, C.H. Lee, M.B. Goodale, M.F. Koff, T.J. Uppstrom, B. Croen, S. Wada, C.B. Carballo, Long-term evaluation of meniscal tissue formation in 3-dimensional–printed scaffolds with sequential release of connective tissue growth factor and TGF-β3 in an Ovine model, The American journal of sports medicine 47(11) (2019) 2596-2607.

[132] X.Z. Zhou, V.Y. Leung, Q.R. Dong, K.M. Cheung, D. Chan, W.W. Lu, Mesenchymal stem cell-based repair of articular cartilage with polyglycolic acid-hydroxyapatite biphasic scaffold, The International journal of artificial organs 31(6) (2008) 480-489.

[133] P. Vahedi, S. Jarolmasjed, H. Shafaei, L. Roshangar, J.S. Rad, E. Ahmadian, In vivo articular cartilage regeneration through infrapatellar adipose tissue derived stem cell in nanofiber polycaprolactone scaffold, Tissue and Cell 57 (2019) 49-56.

[134] J. Zellner, G. Pattappa, M. Koch, S. Lang, J. Weber, C.G. Pfeifer, M.B. Mueller, R. Kujat, M. Nerlich, P. Angele, Autologous mesenchymal stem cells or meniscal cells: what is the best cell source for regenerative meniscus treatment in an early osteoarthritis situation?, Stem cell research & therapy 8(1) (2017) 1-12.

[135] X. Duan, X. Zhu, X. Dong, J. Yang, F. Huang, S. Cen, F. Leung, H. Fan, Z. Xiang, Repair of large osteochondral defects in a beagle model with a novel type I collagen/glycosaminoglycan-porous titanium biphasic scaffold, Materials Science and Engineering: C 33(7) (2013) 3951-3957.

[136] A. Olivos-Meza, F.J. Pérez Jiménez, J. Granados-Montiel, C. Landa-Solís, S. Cortés González, C.A. Jiménez Aroche, M. Valdez Chavez, S. Renán León, R. Gomez-Garcia, V. Martínez-López, First clinical application of polyurethane meniscal scaffolds with mesenchymal stem cells and assessment of cartilage quality with T2 mapping at 12 months, Cartilage 13(1_suppl) (2021) 197S-207S.

[137] A. Gobbi, E. Kon, M. Berruto, R. Francisco, G. Filardo, M. Marcacci, Patellofemoral full-thickness chondral defects treated with Hyalograft-C: a clinical, arthroscopic, and histologic review, The American journal of sports medicine 34(11) (2006) 1763-1773.

[138] Z. Abpeikar, L. Moradi, M. Javdani, S. Kargozar, M. Soleimannejad, E. Hasanzadeh, S.A. Mirzaei, S. Asadpour, Characterization of macroporous polycaprolactone/silk fibroin/gelatin/ascorbic acid composite scaffolds and in vivo results in a rabbit model for meniscus cartilage repair, Cartilage 13(2_suppl) (2021) 1583S-1601S.
